# Record Dissymmetry in Vibrational Optical Activity: Combining Vibronic Coupling and Aggregation in Cobalt(II) Camphorate Complexes

**DOI:** 10.1002/anie.5800931

**Published:** 2026-08-20

**Authors:** Gianmarco Papi, Massimo Guelfi, Stefano Zacchini, Fabio Marchetti, Gennaro Pescitelli

**Affiliations:** ^1^ Dipartimento di Chimica e Chimica Industriale, Università di Pisa Pisa Italy; ^2^ Department of Industrial Chemistry “Toso Montanari” University of Bologna Bologna Italy

**Keywords:** β‐diketonate ligands, circular dichroism, dissymmetry factor, low‐lying electronic states, trifluoroacetylcamphor

## Abstract

A family of cobalt(II) bis‐trifluoroacetylcamphorate complexes (**1–3**), along with the diamagnetic zinc(II) analog **4**, has been synthesized and structurally characterized. The cobalt complexes exhibit solvent‐driven interconversion between *cis*‐ and *trans*‐octahedral geometries, coupled to labile solvent coordination. Their chiroptical response spans the UV, visible, NIR, and IR ranges and is highly sensitive to coordination geometry and solvent. In dilute solutions, vibrational circular dichroism (VCD) dissymmetry factors up to |*g*| ≈4 × 10^−^^3^ reflect vibronic coupling with low‐lying electronic states, a hallmark of high‐spin Co^II^ centers. At concentrations above 30 mM in acetonitrile, spontaneous microcrystallization yields suspensions with an exceptional *g* value of approximately 0.2 at 1600 cm^−^^1^, that is the largest dissymmetry factor ever reported by VCD. This record value arises from the unprecedented concurrence of vibronic and aggregation‐induced enhancement in the same species, with aggregation providing the dominant contribution, as supported by crystallographic, spectroscopic, and computational evidence.

## Graphical abstract

The cobalt(II) bis(trifluoroacetylcamphorate) scaffold exhibits versatile coordination chemistry with solvent‐driven ligand exchange and geometrical rearrangements, associated with unique chiroptical properties. Microcrystalline suspension of [Co(tfc)_2_(CH_3_CN)_2_] revealed the largest VCD dissymmetry factor ever reported (*g* ≈ 0.2 at 1600 cm^−^^1^), arising from an unprecedented combination of vibronic coupling with low‐lying electronic excited states and aggregation‐induced enhancement.

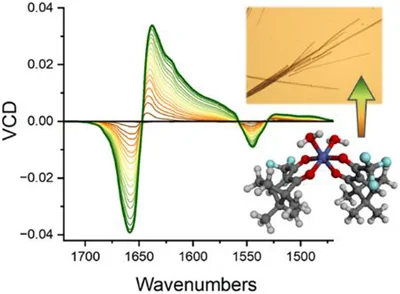

## 1 Introduction

Chiral transition‐metal (TM) complexes play important roles in asymmetric catalysis [1, 2, 3], and they have also emerged as functional materials [4] owing to their modular structures and unique electronic, magnetic, and chiroptical properties [5, 6]. Cobalt‐containing compounds are particularly attractive as sources of chiral‐induced spin selectivity [7], single‐molecule magnets [8], chiral plasmonic materials [9], and chiroptical materials. The chiroptical properties of many cobalt(II) systems are rivalled only by selected lanthanide complexes [10, 11, 12, 13]. The high‐spin *d* ^7^ configuration allows for multiple intraconfigurational transitions in the visible (vis), near infrared (NIR), and, depending on the coordination geometry, even in the IR range (wavenumbers < 4000 cm^–^^1^). The parity‐forbidden nature of *d–d* transitions is allied with magnetic‐allowed and electric‐forbidden character, resulting in electronic circular dichroism (ECD) signals exhibiting large dissymmetry values (*g*‐factors):

(1) $$g=\frac{\Delta A}{A}=\frac{\Delta \varepsilon }{\varepsilon }=4\frac{\mu_{0i}\cdot m_{i0}}{{\left|\mu_{0i}\right|}^{2}+{\left|m_{i0}\right|}^{2}}$$

where *A* and Δ*A* are the absorbance and differential absorbance of left and right circularly polarized light, *ε* and Δ*ε* the corresponding molar quantities, ***μ***_0_ *_i_* and ***m*** *_i_*_0_ the electric and magnetic transition dipole moments. Moreover, *d–d* transitions in the NIR and IR regions give rise to a unique coupling phenomenon with molecular vibrations [14], generating vibrational CD (VCD) [15, 16] signals with exceptional intensity [17, 18]. While discrete organic molecules and diamagnetic TM complexes typically exhibit VCD *g*‐factors (Equation 1) around or below 10^–^^4^ [15], selected cobalt(II) compounds can surpass 10^–^^2^ [19], due to coupling between ligand vibrations and low‐lying electronic states (LLES), providing the strongest VCD enhancements reported for TM systems to date [17]. Another possible source of VCD enhancement is the coupling of multiple vibrational modes in ordered supramolecular assemblies such as fibrils, gels, and crystals [17, 20]. This phenomenon has important implications because of the pathological relevance of amyloid fibril formation, which can be detected by VCD in its early stages [21, 22]. Although the two sources of enhanced VCD, namely vibronic coupling and aggregation, do not exclude each other, to the best of our knowledge their coexistence has never been demonstrated for the same species. This is surprising in view of the variety of supramolecular chiral architectures which can be manifested by TM complexes [23, 24, 25, 26, 27, 28].

β‐Diketonate ligands (dkt, Scheme 1) are versatile platforms for TM and lanthanide complexes exhibiting enhanced chiroptical responses [29, 30, 31, 32, 33, 34, 35, 36, 37]. These include a family of heteroleptic cobalt(II) complexes with two achiral dkt and one bis(oxazoline) (BOX) ligand, exhibiting exceptionally enhanced VCD signals (Scheme 1), that we recently discovered [19]. Various complexes bearing 3‐(trifluoroacetyl)camphorato (tfc) ligands (Scheme 1) have been reported, particularly for lanthanides [38, 39] and some d‐block metal centers [40, 41, 42, 43, 44, 45]. Especially relevant are chiral lanthanide(III) tris‐camphorates discussed by Kaizaki and Sato (Scheme 1) [46, 47], showing enhanced VCD spectra. In contrast, cobalt(II)‐tfc derivatives have appeared only sporadically, often under the generic formulation “Co(tfc)_2_”, and with limited structural characterization and inconsistent synthetic and physical descriptions [48, 49, 50].

**SCHEME 1:**
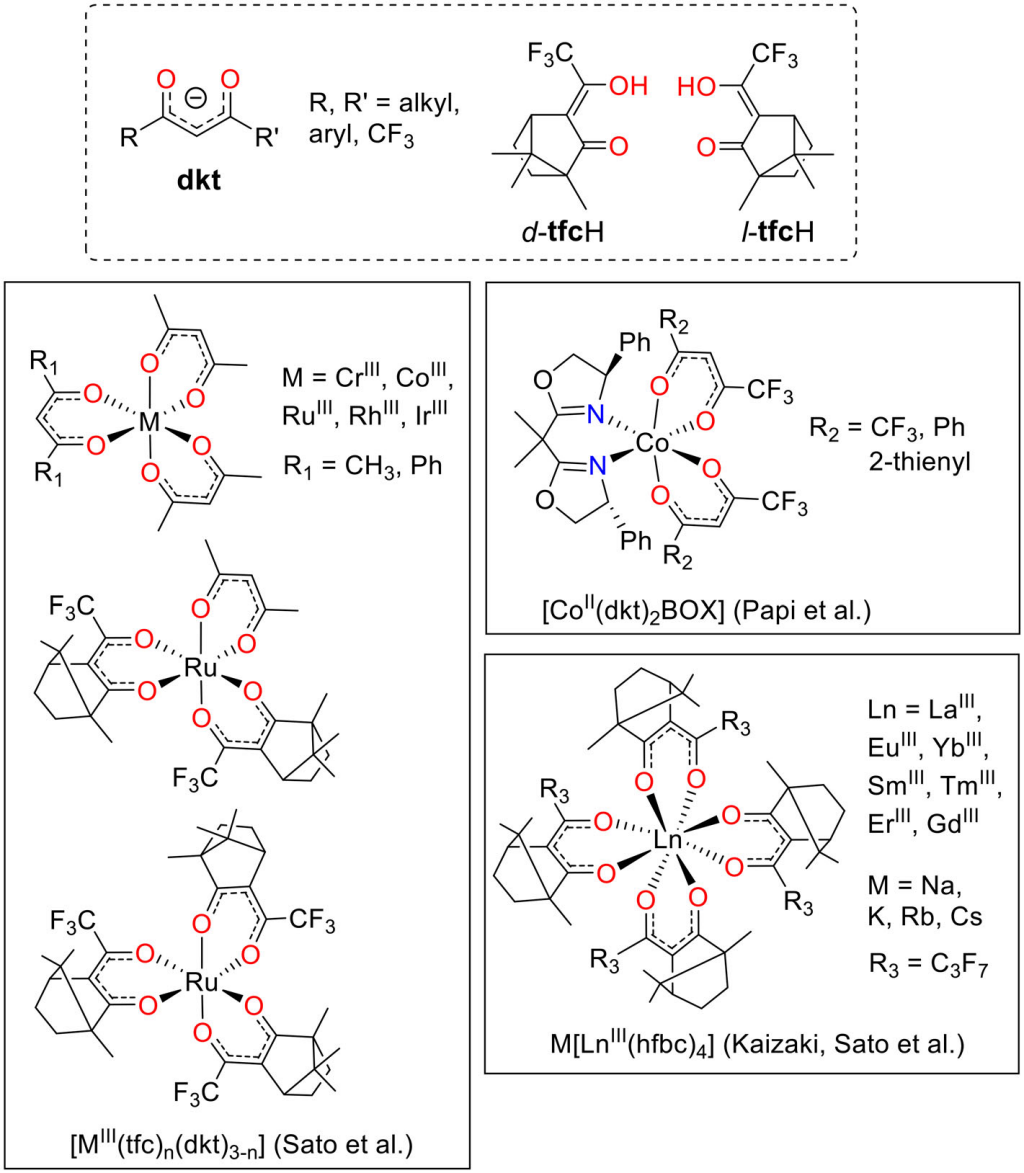
Structures of a generic β‐diketonate ligand (dkt), enantiomeric forms of 3‐(trifluoroacetyl)camphor (tfcH), and selected TM and lanthanide‐dkt complexes characterized by VCD.

Herein, we report a family of cobalt(II)‐tfc complexes **1–3** (Scheme 2) displaying remarkable chiroptical response across the ultraviolet (UV), visible (vis), NIR, and IR regions. Under specific conditions, the bis‐aqua adduct **1** exhibited an unprecedented combination of LLES‐ and aggregation‐enhanced VCD, yielding VCD signals with *g* ≈ 0.2 around 1600 cm^−1^, setting the absolute record among TM complexes and any type of aggregated species.

**SCHEME 2:**
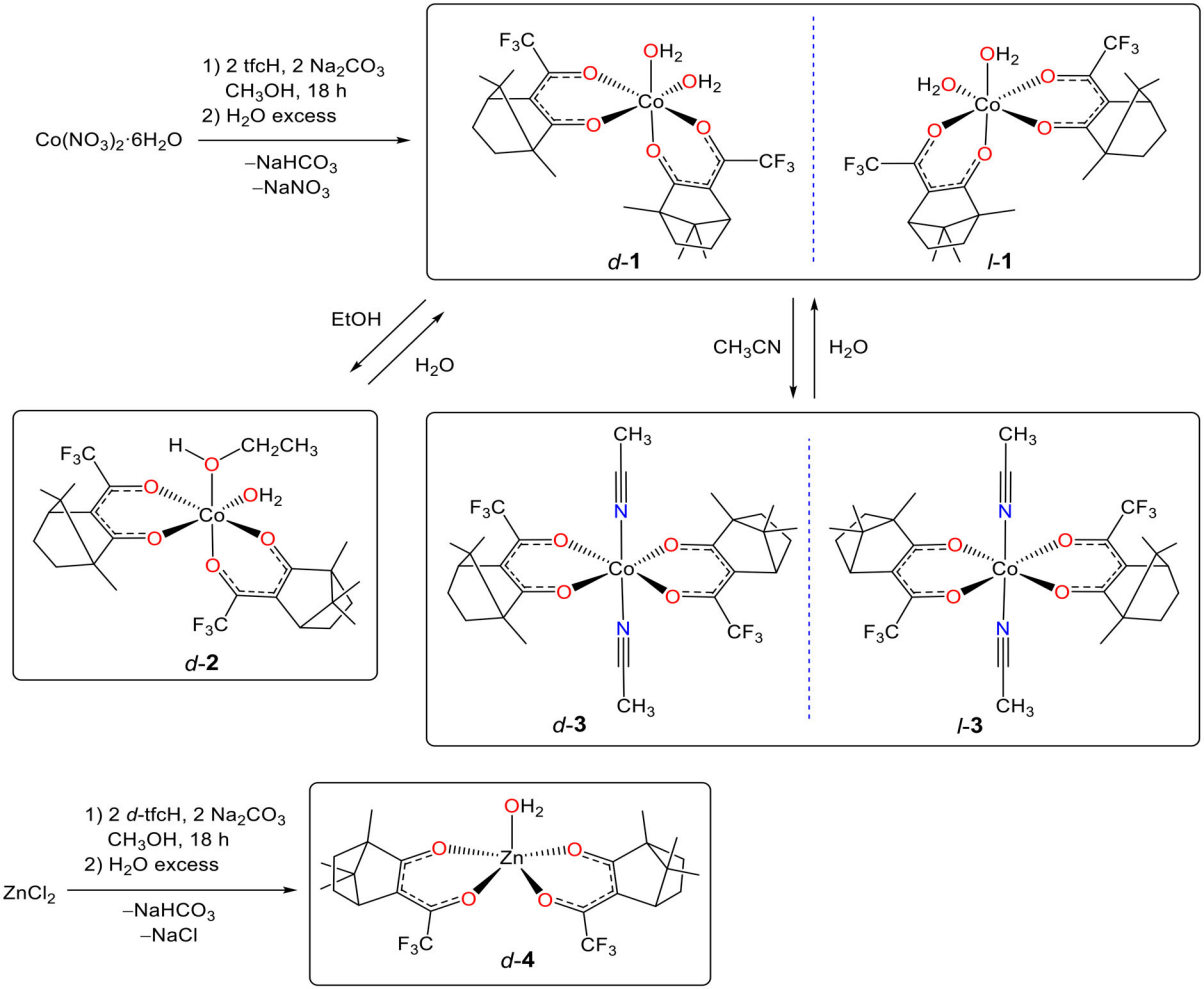
Synthesis of cobalt(II) and zinc(II) 3‐(trifluoroacetyl)camphorate complexes **1–4** and solvent‐controlled interconversion of the cobalt species. The stereochemistry shown corresponds to the solid‐state structures; for **1**, only one diastereomer is represented, for example, Δ‐*l*‐**1** and Λ‐*d*‐**1**.

## 2 Results and Discussion

### 2.1 Synthesis and Structural Characterization

All investigated compounds are shown in Scheme 2 and were found to be stable to oxidation in air for at least several weeks. The enantiomeric ligands derived from (+)‐(1*R*,4*R*) and (–)‐(1*S*,4*S*)‐3‐(trifluoroacetyl)camphor are denoted as *d*‐tfc and *l*‐tfc, respectively (Scheme 1). This shorthand notation refers to the ligand chirality and should not be confused with Δ/Λ referring to chirality at metal. The bis‐aqua complexes *l*‐**1** and *d*‐**1** served as precursors to the remaining chiral cobalt(II) species **2–3**. The zinc(II) complex **4** was prepared as a diamagnetic reference for **1**.

The 1:2 molar reaction of cobalt(II) nitrate hydrate with either enantiomer of tfcH in methanol, in the presence of two equivalents of sodium carbonate, proceeded smoothly to afford a turbid red solution. Addition of excess water induced immediate precipitation of a pink solid, corresponding to *d*/*l*‐**1**, which was isolated in good yields (Scheme 2). The same pink material was reproducibly obtained under these controlled conditions, in contrast to the divergent physical descriptions previously reported for “Co(tfc)_2_”.

Complex *l*‐**1** was characterized by single‐crystal X‐ray diffraction (SC‐XRD) (Figure 1a and Supporting Information). The compound crystallizes in the chiral space group *C*2 with two independent molecules in the asymmetric unit. Both molecules contain the same enantiopure *l*‐tfc ligands adopting an *anti*‐arrangement (i.e., with the CF_3_‐substituted carbonyl groups in mutually *trans* positions) but display opposite chirality at metal (Δ or Λ). Inter‐molecular hydrogen bonds are observed, with the coordinated water molecules acting as donors and the oxygen atoms of the *l*‐tfc ligands as acceptors. These interactions generate infinite chains parallel to the *c*‐axis (Figure S1).

**FIGURE 1:**
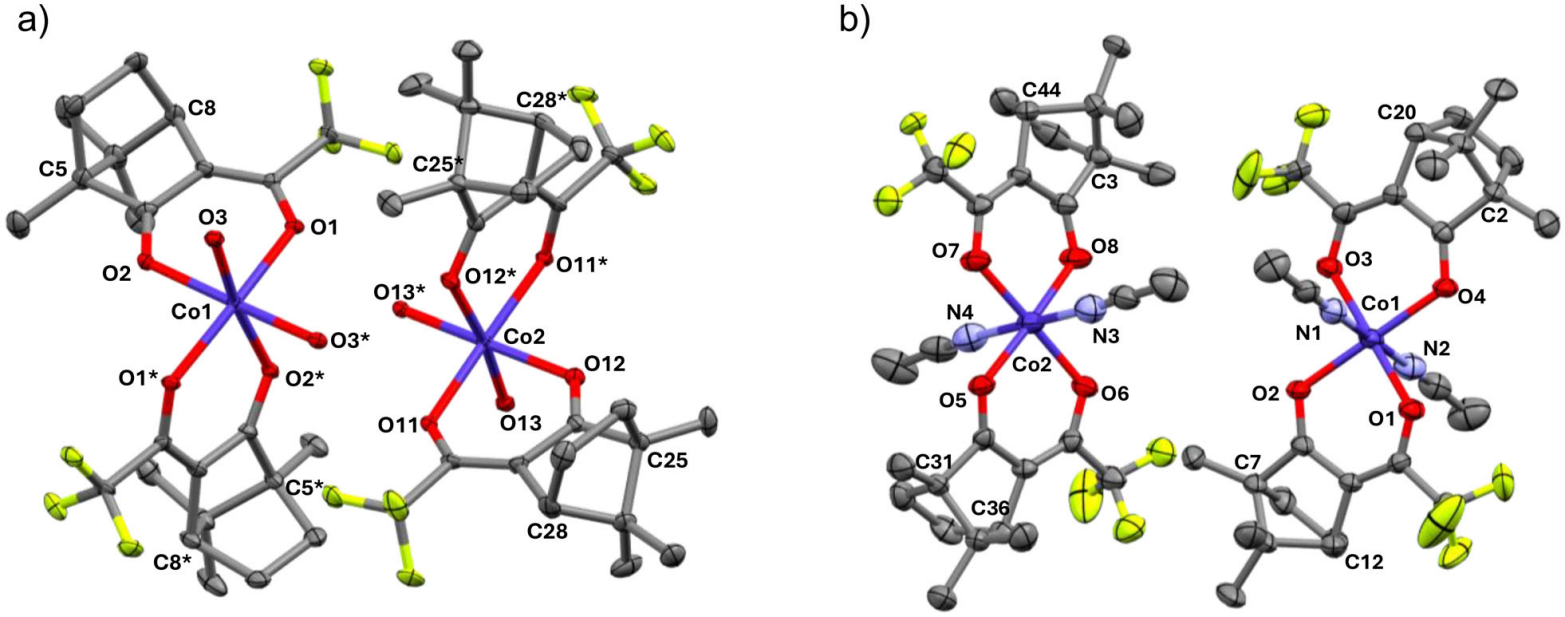
Molecular structure of (a) *l*‐**1** and (b) *l*‐**3** with key atoms labelled, showing the two crystallographically independent molecules in each unit cell. In (a), Co1 and Co2 display opposite metal‐centered configurations (Δ and Λ, respectively); atoms marked with * are generated by a twofold axis passing through the Co center. Displacement ellipsoids are drawn at the 30% probability level, and hydrogen atoms are omitted for clarity. Color code: Co (blue), O (red), C (gray), F (yellow–green), N (light blue).

Cobalt(II) bis‐aqua complexes analogous to **1** incorporating other bis‐diketonates typically exhibit *trans* arrangement of the two aqua ligands [51, 52, 53, 54, 55, 56], although a few *cis* configurations have also been reported [57, 58].

The ^19^F NMR spectrum of *d*‐**1**, in CDCl_3_ at room temperature, displayed two well‐resolved signals around –70 ppm. Based on a previous variable‐temperature ^19^F NMR study on closely related octahedral cobalt(II) bis‐diketonate complexes [59], the observed pattern is indicative of a mixture of *cis*‐type species. This interpretation is consistent with the identification of two diastereomers in the solid state and computational results (vide infra). The pink solid **1** is quantitatively recovered upon evaporation of orange‐red solutions in diethyl ether or dichloromethane. Conversely, evaporation of an ethanol solution afforded a red solid material displaying slightly different elemental analytical values and IR features. Slow evaporation of an ethanol solution of *d*‐**1** yielded single crystals suitable for SC‐XRD, identified as the aqua–ethanol adduct *d*‐**2** (Scheme 2). The formation of *d*‐**2** arises from H_2_O‐EtOH mono‐substitution in *d*‐**1**, while retaining the *cis*‐configuration of the monodentate ligands. Accordingly, the ^19^F NMR spectrum of *d*‐**1** in CD_3_OD differs markedly from that recorded in CDCl_3_, displaying a single sharp resonance at 13.1 ppm, thereby suggesting water‐methanol ligand replacement. However, the formation of the alcohol adducts from **1** appears to be fully reversible, as complex **1** was cleanly recovered by precipitation upon addition of excess water to the alcoholic solutions, in agreement with the synthetic procedure used for its preparation.

Dissolution of **1** in non‐anhydrous acetonitrile afforded a light‐yellow solution corresponding to the bis‐acetonitrile adduct **3** (Scheme 2). Consistently, the ^19^F NMR spectrum of *d*‐**1** in CD_3_CN exhibits a single sharp resonance at ≈33.4 ppm. Upon concentration, **3** slowly precipitates from acetonitrile as yellow crystals, characterized by SC‐XRD (triclinic *P*1 space group, Figure 1b). The two independent molecules in the asymmetric unit feature the CH_3_CN ligands in a mutually *trans* disposition, as in the only other reported [Co(dkt)_2_(CH_3_CN)_2_] complex [56]. This arrangement generates an approximately C_2h_ symmetric coordination environment lacking metal‐centered chirality, in contrast to **1**. Crystal packing is governed by weak O···H and F···H interactions, affording a three‐dimensional supramolecular framework (see Supporting Information for details).

The ^19^F NMR spectrum of *d*‐**3**, in CD_3_CN, is almost identical to that obtained from *d*‐**1** in the same solvent, and shows a single sharp resonance at 33.6 ppm. This suggests that the *trans* coordination geometry observed for *d*‐**3** in the solid state is largely retained in acetonitrile solution. In contrast, in CDCl_3_ *d*‐**3** gives rise to a broad envelope of signals around 10–20 ppm, consistent with interconversion phenomena between two or more isomeric species.

The yellow color of acetonitrile solutions of **3** was persistent; however, once isolated in the solid state, the resulting yellow solid residue proved highly moisture‐sensitive and progressively turned red‐pink due to back‐conversion to **1** (completed within 10–12 h, Figure S36). The final pink solid, dried under vacuum, was unambiguously identified as **1** by IR spectroscopy and elemental analysis. To isolate pure **3**, analytical‐grade acetonitrile was employed under strictly anhydrous conditions, using oven‐dried glassware and N_2_ atmosphere. The solid was handled and stored under N_2_ for several months without detectable degradation.

The environment‐driven ligand switching revealed by the interconversion between **1** and **3** is uncommon in coordination chemistry. Interestingly, the hexafluoroacetylacetonate (hfac) analog of **1**, namely *cis*‐[Co(hfac)_2_(H_2_O)_2_] [52], has been reported to react with acetonitrile in air to give *cis*‐[Co(hfac)_2_(CH_3_CN)_2_], which remains stable toward water during work‐up without undergoing CH_3_CN‐H_2_O exchange [56]. This comparison suggests that the Co(tfc)_2_ platform plays a key role in stabilizing the *cis* aqua‐complex and enabling the observed H_2_O to CH_3_CN exchange. Overall, ^19^F NMR and crystallographic data indicate that **1** is a readily tunable system undergoing ligand exchange in coordinating solvents and solvent‐dependent *cis*/*trans* interconversions.

To support ECD studies (vide infra) and provide a diamagnetic reference, the synthetic procedure used for **1** was extended to zinc. Reaction of zinc dichloride with *d*‐tfcH afforded the bis(β‐diketonate) mono‐aqua complex *d*‐**4** (Scheme 2). The NMR spectra in CDCl_3_ exhibit a single set of resonances, indicating a single species in solution. Crystals suitable for SC‐XRD were collected from a concentrated pentane solution (Figure S8, triclinic space group *P*1). Complex **4** features a locally mirror‐symmetric (C_s_) coordination environment reflecting an achiral zinc(II) center with distorted square pyramidal geometry, where the β‐diketonate ligands occupy the basal plane and the water molecule the apical position. Similar zinc bis(β‐diketonate) mono‐aqua complexes have been previously reported [60, 61, 62].

### 2.2 Chiroptical Spectra

Figure 2 reports the ECD spectra (190‐1600 nm) of *l*‐**1** and *d*‐**1** in acetonitrile and dichloromethane solutions (spectra in methanol are reported in Figure S21). As for other chiral cobalt(II) complexes [11, 19, 63], one may recognize a continuous “superspectrum” of bands spanning from the UV to the vis and NIR regions. The major UV absorption band is allied with π–π* transitions localized on the tfc ligands. The absence of a corresponding ECD exciton couplet [64] suggests either a non‐skewed arrangement of the chromophores or a fluxional situation in solution, where the sense of twist between the two chromophores is not fixed. In principle, this condition is consistent with the coexistence of species with opposite metal‐centered chirality, as observed in the solid state. A clear‐cut exciton couplet between 250 and 400 nm was found in our recently reported [Co(dkt)_2_BOX] complexes (Scheme 1) [19], where the *cis* arrangement between dkt moieties is dictated by the BOX ligand. ECD and, to a minor extent, absorption spectra of *l*‐**1** and *d*‐**1** are solvent‐dependent, particularly in the vis and NIR regions dominated by metal‐centered *d–d* transitions. In a *d* ^7^ high‐spin octahedral field, three transitions are expected, of ^4^ *T*_1g_(*P*)←^4^ *T*_1g_(*F*), ^4^ *A*_2g_←^4^ *T*_1g_(*F*) and ^4^ *T*_2g_←^4^ *T*_1g_(*F*) types, respectively, moving from high to low energy [65, 66]. Upon symmetry lowering, triple degeneracy is removed, and these transitions split into a manifold of bands sensitive to the coordination geometry. The apparent changes in the ECD spectra from CH_2_Cl_2_ to CH_3_CN and CH_3_OH may result from exchange of one/two water ligands with solvent molecules and/or geometrical rearrangements around cobalt. The maximum *g*‐values recorded for **1** are approximately 10^−3^ in the UV, 2 × 10^−3^ in the vis, and 2 × 10^−2^ in the NIR regions, comparable to other discrete chiral cobalt(II) complexes [11, 19, 63].

**FIGURE 2:**
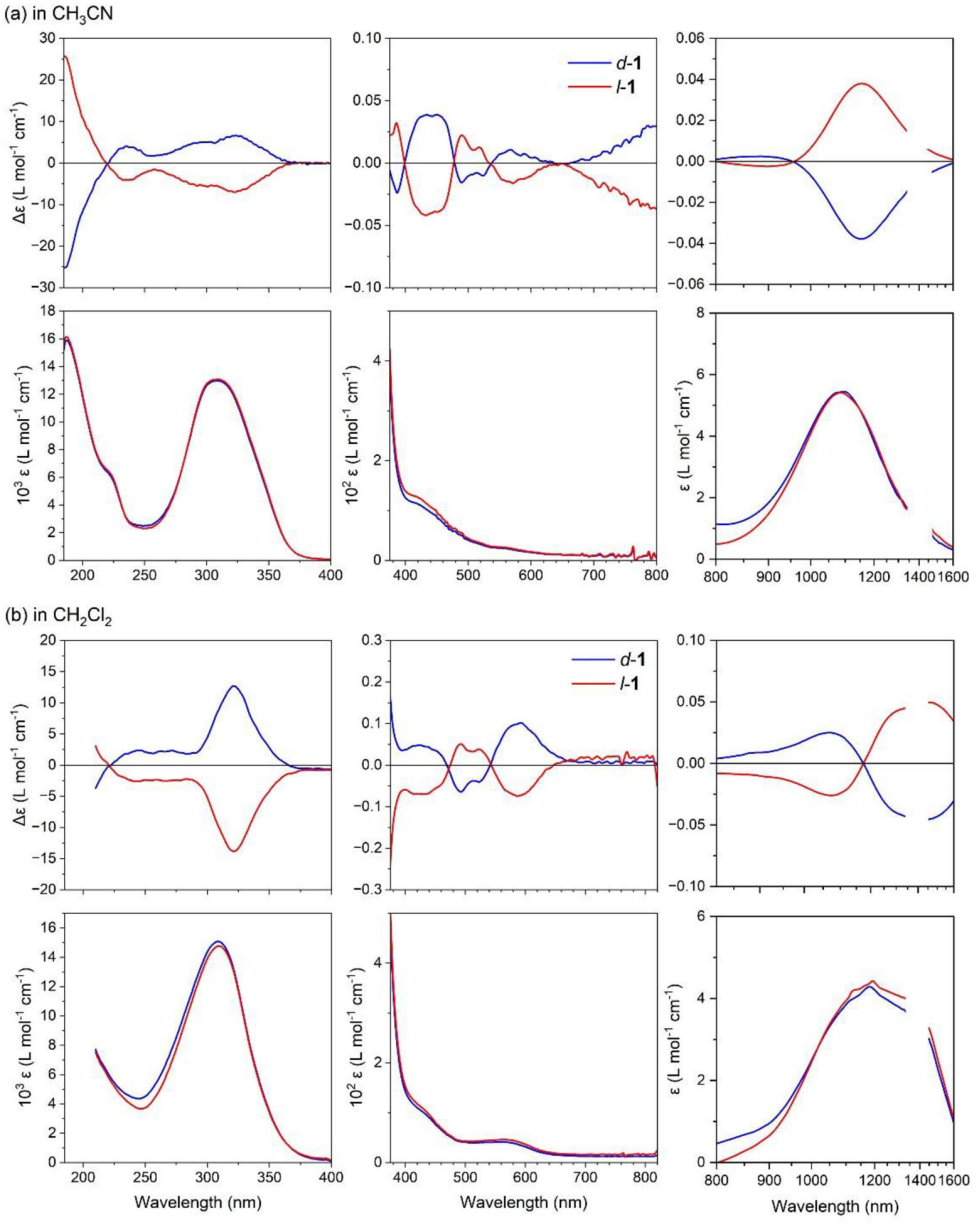
Optical (bottom) and chiroptical (top) superspectra of *l*‐**1** (red) and *d*‐**1** (blue) in the UV, vis and NIR regions (left to right), in CH_3_CN (a) and CH_2_Cl_2_ (b). Deleted portions in the NIR region correspond to spectral areas masked by solvent absorption.

To clarify the solution structure and chiroptical properties of **1**, complexes **2** and **3** were also characterized. To probe the water‐to‐acetonitrile ligand exchange, samples of [Co^II^(tfc)_2_(CH_3_CN)_2_] (**3**) were prepared and measured under strictly anhydrous conditions. The ECD spectra of *l*‐**1**/*d*‐**1** and *l*‐**3**/*d*‐**3** in 3 mM CH_3_CN and CH_2_Cl_2_ solutions were similar (Figure 2 and S22), indicating a consistent dominant species. The absence of an exciton couplet supports a dominant *trans* chelate geometry for *l*‐**3**/*d*‐**3**, in accord with NMR and X‐ray data. Conversely, [Co^II^(*d*‐tfc)_2_(H_2_O)(EtOH)] (*d*‐**2**) displayed substantially different ECD spectra in CH_2_Cl_2_ (Figure S24), including an asymmetric exciton couplet between 250 and 340 nm. This hints at a dominant *cis* chelate geometry with negative sense of twist between π–π* transitions of tfc ligands, in accord with the X‐ray structure. Overall, ECD data suggest that **3** preserves its solid‐state *trans*‐chelate geometry in CH_3_CN solution, whereas **1** undergoes water‐to‐acetonitrile ligand exchange accompanied by geometrical rearrangement toward the same *trans*‐chelate geometry as in **3**. Additionally, [Zn^II^(*d*‐tfc)_2_(H_2_O)] (*d*‐**4**), featuring a filled *d* shell, exhibited absorption and ECD bands only in the UV region (Figure S25) as expected, with no evidence of exciton coupling, in keeping with the pyramidal geometry revealed by X‐ray diffraction.

The most relevant results for cobalt(II) species came from VCD measurements. Figure 3 shows the IR and VCD spectra of *l*‐**1** and *d*‐**1** between 1800 and 1000 cm^–^^1^ in 15 mM CD_3_CN and CDCl_3_ solutions (see Figure S26 for *l*‐**3** and *d*‐**3**). The apparent discrepancies in both IR and VCD profiles between the two solvents witness once again distinct ligand environments and coordination geometries. In the fingerprint region, VCD bands reach maximum intensity around |Δε| ≈ 0.5 M^−1^ cm^−1^, corresponding to maximum |*g*| ≈ 4 × 10^−3^ at 1495 cm^−1^ in CD_3_CN and 3.6 × 10^−3^ at 1262 cm^−1^ in CDCl_3_. Such dissymmetry values are ≈1 order of magnitude larger than those commonly observed for organic compounds and diamagnetic metal complexes [15]. For comparison, *d*‐**4** exhibits a maximum |*g*| ≈ 10^−3^ at 1360 cm^−1^ (Figure S27); however, as already noticed, **1** and **4** are not isostructural. As discussed in the Introduction, enhanced VCD signals are often encountered for high‐spin *d* ^7^ cobalt(II) complexes due to the occurrence of LLES [14, 17]. Compared to our previously reported [Co^II^(dkt)_2_BOX] series (Scheme 1) [19], **1** features a substantially weaker enhancement, by approximately 1 order of magnitude in terms of absolute VCD intensity and *g*‐factor. This observation correlates with the absence of electronic *d–d* transitions in the IR range, unlike [Co^II^(dkt)_2_BOX] complexes. For the latter, electronic transitions were observed between 1700 and 2500 cm^–^^1^ which allowed for strong vibrational‐electronic coupling yielding extraordinarily enhanced VCD [19]. No ECD bands were instead detected for *l*‐**1** and *d*‐**1** beyond the NIR range (Figure 3 and Figure S28). Similar results were observed for *l*‐**3** and *d*‐**3** (Figure S26). These findings indicate that the Co^II^(tfc)_2_(L)_2_ scaffold (L = ancillary ligand such as H_2_O or CH_3_CN) does not support very low‐lying metal‐centered excited states, contrary to the Co^II^(dkt)_2_BOX system.

**FIGURE 3:**
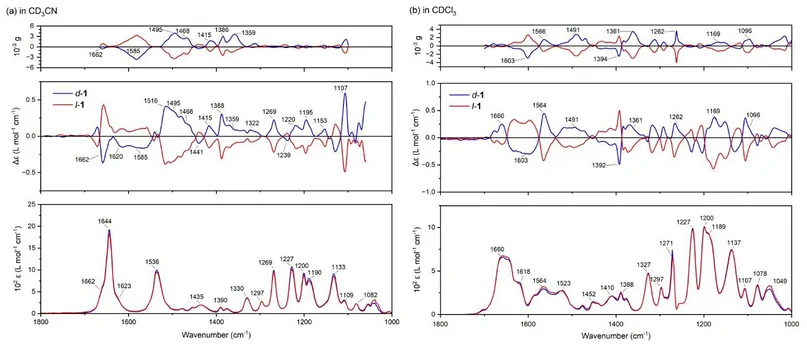
IR (bottom), VCD (middle) and *g*‐value traces (top) of *l*‐**1** (red) and *d*‐**1** (blue), measured on 15 mM solutions in CD_3_CN (a) and CDCl_3_ (b); cell path‐length 200 µm.

VCD measurements of *l*‐**1** and *d*‐**1** in more concentrated (40 mM) CD_3_CN solutions held, however, a surprise. In contrast to dilute solutions, whose spectra were stable over time, the VCD signal gradually grew in intensity, yielding after 30–40 min a stationary profile of exceptional intensity (Figure 4), while the corresponding IR signals remained unchanged. Because both IR and VCD scale linearly with concentration under the Beer‐Lambert law, a purely concentration‐driven increase would leave the *g*‐factor unaffected. The observed VCD growth at constant IR absorbance therefore rules out a simple concentration effect and points to a supramolecular, aggregation‐driven origin. The progressive VCD increase was evident throughout the fingerprint region and rather spectacular for a pair of bands between 1590 and 1700 cm^–^^1^, which can be described as a VCD couplet [67] with positive sign for *d*‐**1** and negative sign for *l*‐**1** (Figure S29). Here, the corresponding *g*‐factor increases from ≈ 7 × 10^–^^4^ at 1660 cm^−1^ (15–20 mM solution) to ≈ +5 × 10^−2^ for the positive band at 1636 cm^−1^ and ≈ –5 × 10^−2^ for the negative band at 1658 cm^−1^ (40 mM solution after 45 min). Even larger |*g*| values are observed on the shoulders of the couplet, reaching a maximum *g* ≈ +0.2 at 1600 cm^−1^ (Figure 4). On a merely experimental ground, this value represents by far the largest dissymmetry factor ever measured by VCD, to the best of our knowledge [17]. The previous record among transition metal complexes was *g* = 5.4 × 10^−2^ at 1320 cm^–^^1^ for a member of the [Co^II^(dkt)_2_BOX] family [19], while guanosine crystals reached the highest value reported for any system so far, namely *g* = (7.3 ± 2) × 10^−2^ [68]. Thanks to their exceptional intensity, VCD spectra of microcrystal suspensions of **1** can be acquired with a minimal number of scans: even a single scan afforded a perfectly defined VCD spectrum with S/N > 40 around 1650 cm^−1^ (Figure S33).

**FIGURE 4:**
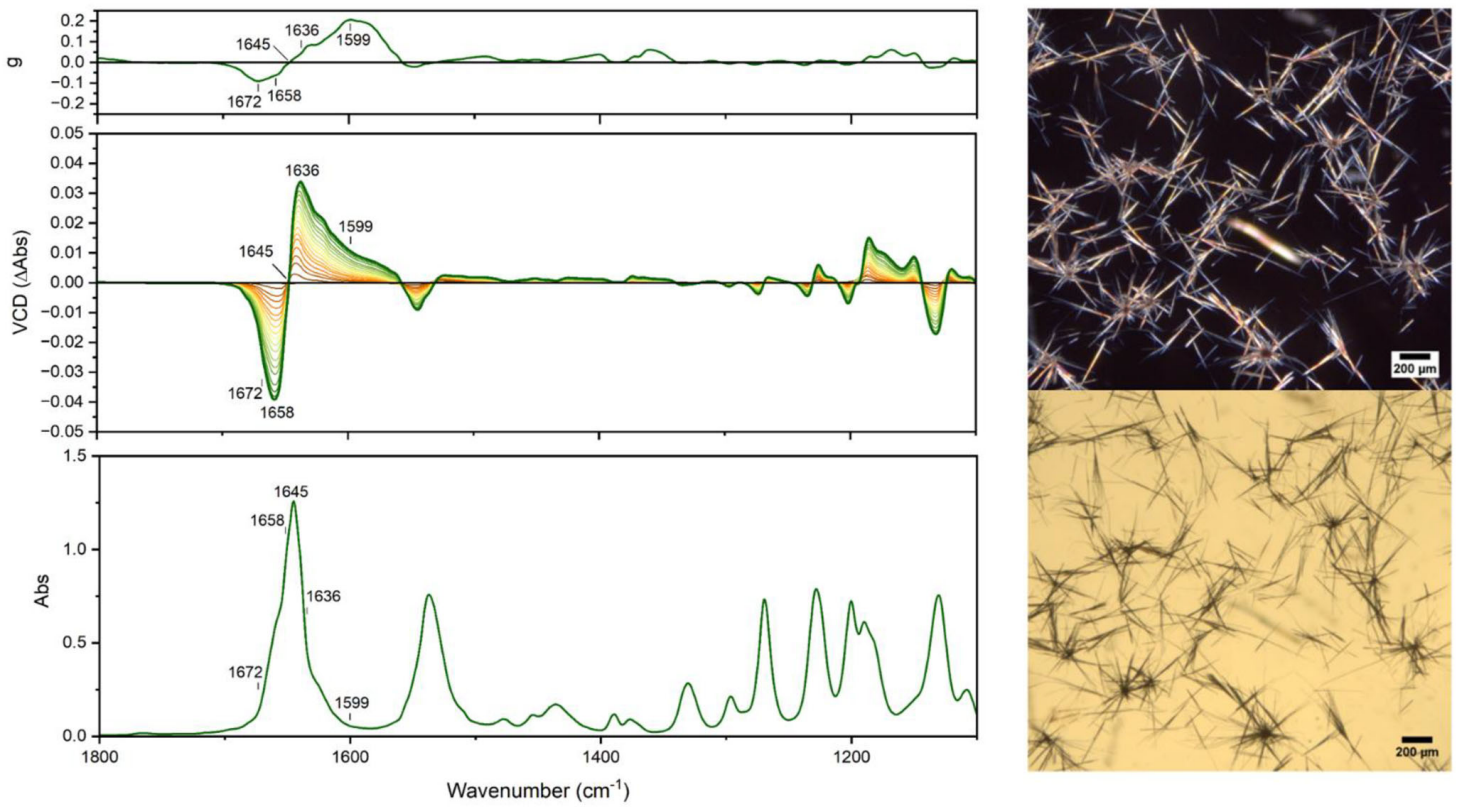
Left: time evolution of IR (bottom) and VCD spectra (middle) of *d*‐**1** in 40 mM CD_3_CN over 45 min, with a 200 µm path‐length cell. The top graph shows the *g*‐value trace of the stationary spectrum, while in the bottom graph all IR traces are coincident. Right: optical microscopy images of microcrystals in bright field (bottom) and under cross‐polarized filters (top)

In the above conditions (> 30 mM yellow CD_3_CN solution), microcrystallization occurs during the time necessary to achieve stationary and strongly enhanced VCD spectra. Yellow needle‐type microcrystals form inside the VCD cell with an average thickness of 5–10 µm and lengths up to a few mm (Figure 4 and Figures S37–S38). This process is fully reproducible across distinct batches, and the stationary VCD profile is consistently obtained, although with some variation in the final intensity (within 20%–30%). After solvent evaporation, the VCD spectrum of microcrystals deposited on the (demounted) cell walls remains similar to that of the microcrystal dispersion. However, prolonged exposure to air causes a color change from yellow to pink (characteristic of the starting material). The VCD spectrum measured on this pink solid is sizably different from that of the original yellow microcrystalline phase (Figure S30). A similar micro‐crystallization phenomenon was also observed in CDCl_3_ (> 30 mM), yielding enhanced VCD spectra with different shape than in CD_3_CN and slightly smaller dissymmetry values (maximum |*g*| ≈ 9 × 10^−2^ at 1555 cm^−1^, Figure S31).

The yellow microcrystals formed in the VCD cell were too small for direct SC‐XRD analysis. However, when a 15 mM solution of **1** in CH_3_CN was left to stand at –4°C for several weeks, larger crystals were obtained whose X‐ray characterization enabled the identification of [Co^II^(tfc)_2_(CH_3_CN)_2_] (**3**). Not surprisingly then, VCD spectra of *l*‐**3** and *d*‐**3** measured in CD_3_CN closely match those of *l*‐**1** and *d*‐**1** in the same solvent, also in agreement with NMR data. Furthermore, suspensions obtained by partial dissolution of **3** in CD_3_CN (nominal concentration 40 mM) yielded enhanced VCD spectra with the same shape as in Figure 4 for **1**. In this case, however, no time evolution occurs because crystals or microcrystals are already preformed, and the VCD intensity is lower (Figure S32). VCD data provide clear evidence that the aqua complex **1** in CH_3_CN undergoes ligand exchange to form the bis‐acetonitrile adduct **3**, which at concentrations > 30 mM forms a microcrystalline suspension exhibiting extraordinarily large VCD signals.

Summarizing, the enhanced VCD profile in Figure 4 is the consequence of two consecutive processes: (1) the conversion of the aqua complex **1** into the acetonitrile complex **3**; (2) the growth of microcrystals of **3** within the cuvette. The first process is immediate and occurs during sample preparation; the second proceeds more slowly and is complete within 45 min. The aggregation phenomenon produces a VCD spectrum with progressively increased intensity and *g*‐factor at constant IR signal, reaching a maximum |*g*| ≈ +0.2 at 1600 cm^−1^. The VCD spectrum of non‐aggregated **3** (Figure S26) is qualitatively different and retains only the enhancement due to the vibronic coupling (maximum |g| ≈ 4 × 10^−^^3^ at 1495 cm^−1^).

**FIGURE 5:**
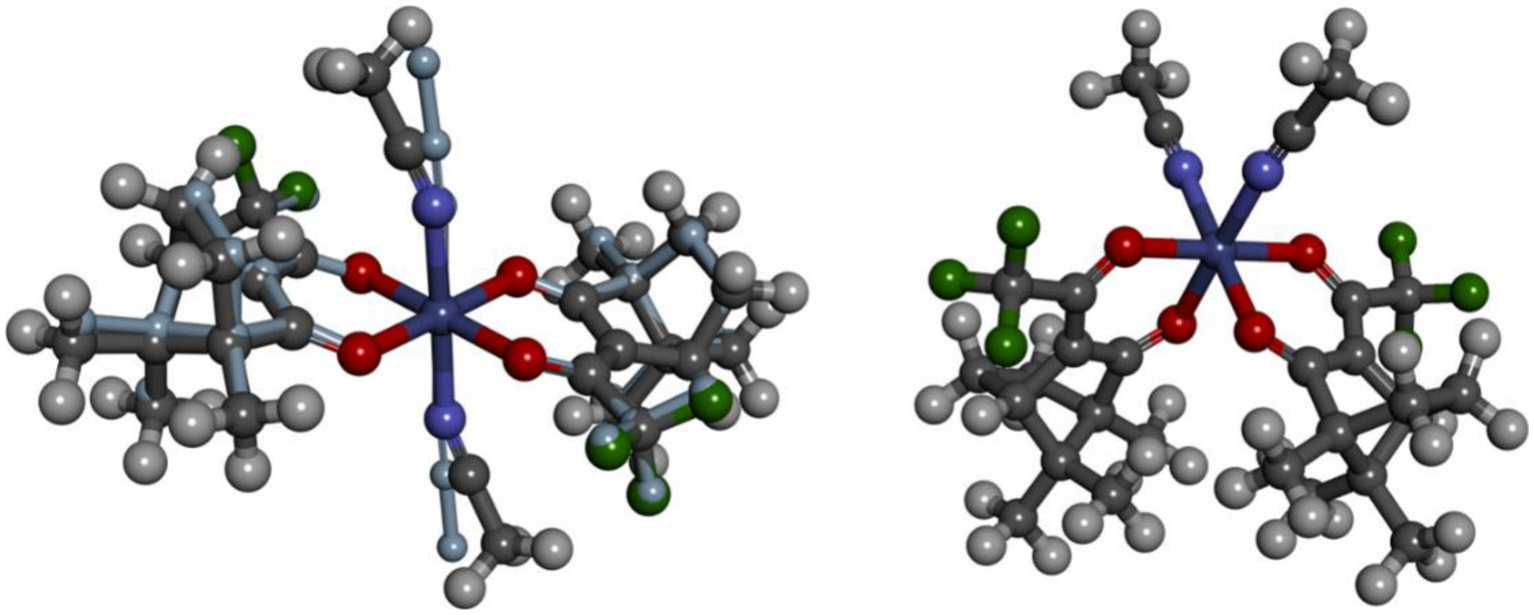
DFT‐optimized structures of *l*‐**3**, B3LYP‐D3BJ/def2‐SVP/PCM level. Left: *trans* chelate isomer obtained by optimization of the X‐ray geometry (superimposed in light blue with hydrogens removed). Right: *cis* chelate isomer (lowest‐energy calculated structure).

### 2.3 Calculations

A computational analysis was run on **1** and **3** with density functional theory (DFT) and time‐dependent DFT (TD‐DFT). Geometry optimizations were performed on *l*‐**1** and *l*‐**3** at B3LYP‐D3BJ/def2‐SVP level, both in vacuo and including a continuum solvent model (IEF‐PCM) for CH_2_Cl_2_ and CH_3_CN. Direct optimization of the X‐ray geometry of *l*‐**1** produced only minor structural changes. Several isomers were found differing in metal‐centered chirality (Λ / Δ), chelate coordination geometry (*trans*/*cis*) and tfc arrangement (*anti*/*syn*) (Table S10). In accord with the ^19^F NMR spectrum of *d*‐**1** in CDCl_3_ and with ECD spectra indicating coexistence of species with opposite chirality at metal, at least 4 distinct *cis* isomers displayed calculated population > 10% at 300 K (2 pairs of complexes with Λ and Δ metal configurations each). Optimization of the X‐ray geometry of *l*‐**3** afforded Co–O and Co–N distances close to the experimental values. The major difference was the increased inclination of the acetonitrile N–C–C axis relative to the *z*‐axis (Figure 5). The dihedral angle between the O···O directions of opposite tfc ligands was < 1°, implying negligible exciton‐coupled ECD. Alternative isomers are also possible for **3** (Table S11). In terms of enthalpy and free energy, *cis* complexes prevail over *trans* ones, partly due to the much larger dipole moment (7.5–14 D vs. 0.5–4 D) stabilized by the PCM solvent field, and partly because of attractive dispersion forces between tfc hydrogen atoms in the *cis* structures (Figure 5). These effects are not compensated by the implicit solvent model, which fails to capture the specific solute‐solvent interactions that stabilize the *trans* isomer in solution. The use of a different solvation model (SMD), functional (M06‐L), and larger basis set (def2‐TZVP, SDD with ECP for Co) did not revert the stability order between *trans* and *cis* isomers. Therefore, DFT geometry optimizations fail to reproduce the structural picture provided by ECD and NMR data for **3**.

In light of this discrepancy, the simulation of chiroptical properties was performed on two representative structures of *l*‐**3** (Figure 5), one with *trans* chelate geometry corresponding to the DFT‐optimized X‐ray geometry, and one with *cis* chelate geometry corresponding to the lowest‐energy conformer with Λ chirality at metal (40% population at 300 K based on free energies). TD‐DFT calculations were run with different functionals (B3LYP, CAM‐B3LYP and M06‐L) and basis sets (def2‐TZVP or def2‐SVP for all atoms except Co, SDD or LanL2DZ with the respective ECP for Co), including the IEF‐PCM model for CH_3_CN. The first electronic transition of **3** was predicted at 1400–1460 nm at M06‐L/def2‐TZVP/LanL2DZ level. The same combination of functional and basis set yielded 5625 nm for [Co(dkt)_2_BOX] [19]. Thus, calculations agree with the experimental observation that the first electronic transition occurs in the IR for [Co(dkt)_2_BOX] and in the NIR for **3**, and with the very different degrees of LLES‐related VCD enhancement. Moreover, the ECD spectra calculated for the two representative isomers of *l*‐**3** differ substantially; the *trans* isomer provides better agreement than the *cis* one with the experimental ECD spectra of *l*‐**1** and *l*‐**3** in CH_3_CN, supporting predominance of the *trans* form in solution despite its higher relative DFT energy. Consistent with previous reports on Co^II^ complexes [11, 19, 63, 69] and other open‐shell TM complexes [70], TD‐DFT calculations on the present high‐spin *d* ^7^ complex were complicated by the large mixing between ligand‐ and metal‐centered transitions and the high number of roots needed to reach the UV region (up to 120). Among the tested functionals, M06‐L showed the best performance, B3LYP performed reasonably well, whereas CAM‐B3LYP performed poorly. Figure 6 reports the ECD spectrum calculated at M06‐L/def2‐TZVP/LanL2DZ level for the *trans* geometry of *l‐* **3**, showing fair agreement with Figure 2a and Figure S22. Notably, NIR‐CD bands are well reproduced in sign, relative intensity, and position, although the absolute energies are underestimated by ≈0.18 eV at this level.

**FIGURE 6:**
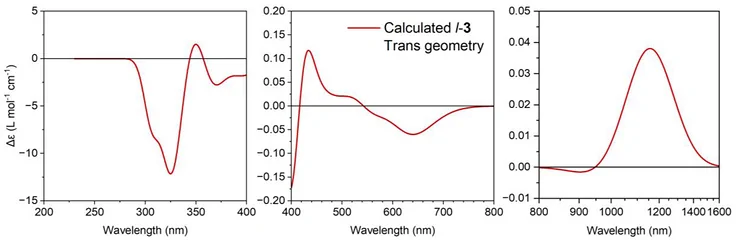
ECD spectra calculated for the *trans* isomer of *l*‐**3** at M06‐L/def2‐TZVP/LanL2DZ(Co) level, including PCM for CH_3_CN. The spectra are red‐shifted by 50 nm in the UV and vis regions and blue‐shifted by 250 nm in the NIR region. See Supporting Information for details.

To better evaluate the exciton coupling between π–π* transitions of tfc ligands, avoiding complications associated with open‐shell species, we replaced Co^II^ by Zn^II^ in the two representative structures of *l*‐**3**. As expected, the *trans* isomer of the resulting hypothetical [Zn(*l*‐tfc)_2_(CH_3_CN)_2_] species displays a calculated ECD spectrum with a single negative π–π* band at 280 nm (Figure S34), consistent in sign and intensity with the experimental spectra of *l*‐**1** and *l*‐**3** in CH_3_CN. Conversely, the *cis* isomer of [Zn(*l*‐tfc)_2_(CH_3_CN)_2_] shows an intense positive couplet in the π–π* region, which has no experimental counterpart from either *l*‐**1** or *l*‐**3** (Figure S34). This outcome further supports the predominance of *trans*‐**3** over *cis*‐**3** in acetonitrile solution.

Frequency calculations were run on *l*‐**3** at the same B3LYP‐D3BJ/def2‐SVP/PCM level employed for geometry optimizations. While the calculated IR spectra reproduce well the experimental spectra of *l*‐**1** and *l*‐**3** in diluted CD_3_CN solutions (Figure 3), the calculated VCD spectra share no similarity with the experimental ones (Figure 7). The IR band observed at 1644 cm^–^^1^ in solution is assigned to the symmetric C═O stretching of the two carbonyl groups within each tfc ligand, involving anti‐phase coupling between the two *trans*‐oriented tfc moieties (Figure 7, right). On the high wavenumber side, the in‐phase coupling normal mode is predicted with lower intensity. These two normal modes are associated with the most strongly enhanced VCD bands observed in the aggregated‐state spectrum (Figure 4). The calculated VCD profile differs markedly from the experimental one in sign and intensity of bands. This result indirectly supports the occurrence of coupling between ligand‐centered vibrations and metal‐centered LLES [14, 15]. In fact, explicit electronic‐vibrational coupling terms are not included in the molecular Hamiltonian in the magnetic field perturbation (MFP) theory for VCD [71] used by Gaussian 16 [72], therefore LLES‐related effects cannot be captured [10, 11, 19, 29, 31]. Two recent approaches hold promise for the treatment of LLES‐related VCD enhancements in discrete species. Tomeček and Bouř used a Herzberg–Teller expansion of the electronic wave function and incorporated non‐adiabatic interaction terms (Born‐Oppenheimer corrections) directly into a model Hamiltonian [73]. More recently, Sapova et al. applied Nafie's vibronic coupling theory [14] using a Sum‐Over‐States (SOS) formalism to calculate specific corrections for the Atomic Polar Tensors (APT) and Atomic Axial Tensors (AAT), using empirical excitation energies [74]. Both approaches successfully reproduced the VCD spectrum of [Co^II^(sp)Cl_2_] (sp = κ^2^ *N*‐sparteine), the first example of VCD enhancement in a TM complex [75, 76]. Nevertheless, Bouř’s approach could not reproduce the peculiar monosignate appearance of a Co^II^ salicylaldiminato reported by one of us in 2018 [11], whereas Sapova et al. did not test their methodology beyond [Co^II^(sp)Cl_2_] and [Ni^II^(sp)Cl_2_]. Therefore, reproducing the solution spectrum of *l*‐**1** or *l*‐**3** in diluted CD_3_CN is still not viable based on the current implementations of VCD theory. The situation is even more complicated for the spectrum in concentrated CD_3_CN solution, which is of supramolecular origin, as discussed below.

**FIGURE 7:**
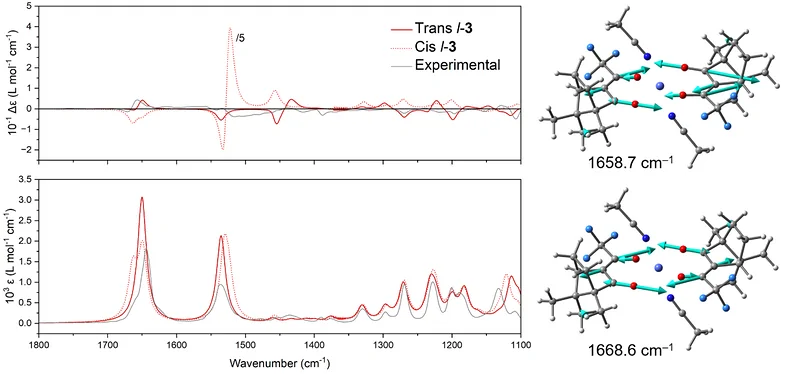
Left: calculated IR (bottom) and VCD spectra (top) for the *trans* and *cis* isomers of *l*‐**3** compared with the experimental spectra of *l*‐**3** in 15 mM CD_3_CN. Calculation level B3LYP‐D3BJ/def2‐SVP/PCM(CH_3_CN), frequency scaling factors 0.965 (> 1400 cm^–^^1^) and 0.98, Lorentzian bandwidth = 8 cm^−1^. Right: displacement vectors for the normal modes contributing to the IR and VCD bands above 1650 cm^−1^ for *trans*‐*l*‐**3**.

### 2.4 Source of VCD Enhancement

With reference to the system providing the strongest VCD intensity, namely *d*‐/*l*‐**1** in CD_3_CN, we measured maximum |*g*| ≈ 4 × 10^−3^ at 1495 cm^−1^ in diluted solutions and |*g*| ≈ 0.2 at 1600 cm^−1^ as microcrystal suspension. Assuming a threshold of |*g*| = 10^−4^ to define the occurrence of a VCD enhancement phenomenon [17], the enhancement factor is about 40 for *d*‐/*l*‐**1** in dilute solution and as large as 2000 in microcrystal suspension. The first type of enhancement is due to the presence of LLES typical of high‐spin cobalt(II) *d* ^7^ systems and is characteristic of discrete species in solution. The second type must instead be related to aggregation. Enhanced VCD signals have been reported in the literature for several aggregated systems [17], including protein and polypeptide fibrils [20, 21, 22], oligopeptide hydrogels [77, 78], organogels [79, 80, 81], and crystals [68, 82, 83]. Table S12 summarizes the largest *g*‐values observed for each family. Several theoretical and/or computational studies have demonstrated that enhanced VCD is a collective property of protein fibrils [84, 85, 86, 87, 88, 89], gel phases [80, 90] and molecular crystals [68, 91, 92, 93] associated with long‐range couplings between vibrational oscillators made possible by the regular fibril, gel or crystal structure.

The large VCD enhancement observed for microcrystals of **1** and **3** can be rationalized along similar lines. The long‐range nature of the observed enhancement is evidenced by the different time evolution of the IR and VCD spectra in Figure 4. While the IR spectra remain unchanged during microcrystal growth, the VCD intensity increases by >1 order of magnitude over 30–40 min. This indicates that the IR spectra reach their final profile at the early stages of microcrystal formation, reflecting the first levels of supramolecular organization. In contrast, VCD is dominated by long‐range couplings arising at the nanoscale, possibly extending to the microscale, and continues to evolve until microcrystal growth is complete. It is well established that chiroptical spectroscopies respond more sensitively to intermediate aggregation stages (nucleation) than their achiral counterparts, which are instead affected especially in early stages (pre‐nuclei formation) [94, 95, 96].

A rigorous theoretical treatment of the present system would require periodic quantum calculations with nuclear velocity perturbation theory (NVPT) coupled to a sampling procedure based on ab initio molecular dynamics (AIMD) [91, 97], which is beyond the scope of this work. Moreover, such a procedure would not account for the LLES‐related enhancement, whose contribution might not be merely additive but act synergistically with the aggregation‐related enhancement. Qualitatively, the most strongly enhanced normal modes are associated with symmetric C═O stretching vibrations of the two carbonyl bonds. Beyond the in‐phase and anti‐phase intra‐molecular couplings occurring within each [Co(tfc)_2_(CH_3_CN)_2_] unit, these modes couple inter‐molecularly between molecules packed in the crystal, favored by the distortion of each independent molecule (molA and molB), and by their reciprocal tilt in the unit cell. Thus, the solid‐state geometry allows VCD‐active intramolecular and intermolecular vibrational exciton couplings between C═O stretching vibrations. This is demonstrated by frequency calculations on model systems composed of [Zn(tfc)_2_(CH_3_CN)_2_] units fixed in the crystallographic position of **3** within one unit cell (“dimer”) or two adjacent unit cells (“tetramers”). A similar minimal approach has been employed by Rode et al. to simulate solid‐state VCD spectra of a chiral thiophene sulfonamide [98]. To preserve the solid‐state arrangement, all Zn (replacing Co), O, and N atoms were kept frozen; therefore, some low‐energy imaginary frequencies were detected without compromising the emerging picture. Figure 8 shows that, in the symmetric C═O stretching region, a negative VCD couplet with increasing intensity is obtained when passing from the DFT‐optimized structure to single molecules in their distorted solid‐state geometry, then to the “dimer” within the unit cell and finally to the “tetramers” in two neighboring unit cells. The couplet sign is always negative, in agreement with the experiment for dispersed microcrystals of *l*‐**3**. More interestingly, the calculated *g*‐factor increases almost linearly with the number of molecules. In the absence of significant intermolecular couplings, the *g*‐factor (Equation 1) would remain roughly independent of the number of molecules included in the calculation. These findings confirm that intermolecular couplings may be responsible for strong VCD couplets above 1600 cm^−1^. However, closest‐neighbor interactions alone cannot account for the exceptional aggregation‐induced VCD enhancement, whose origin should be sought in long‐range effects, possibly acting synergistically with LLES‐related enhancement.

**FIGURE 8:**
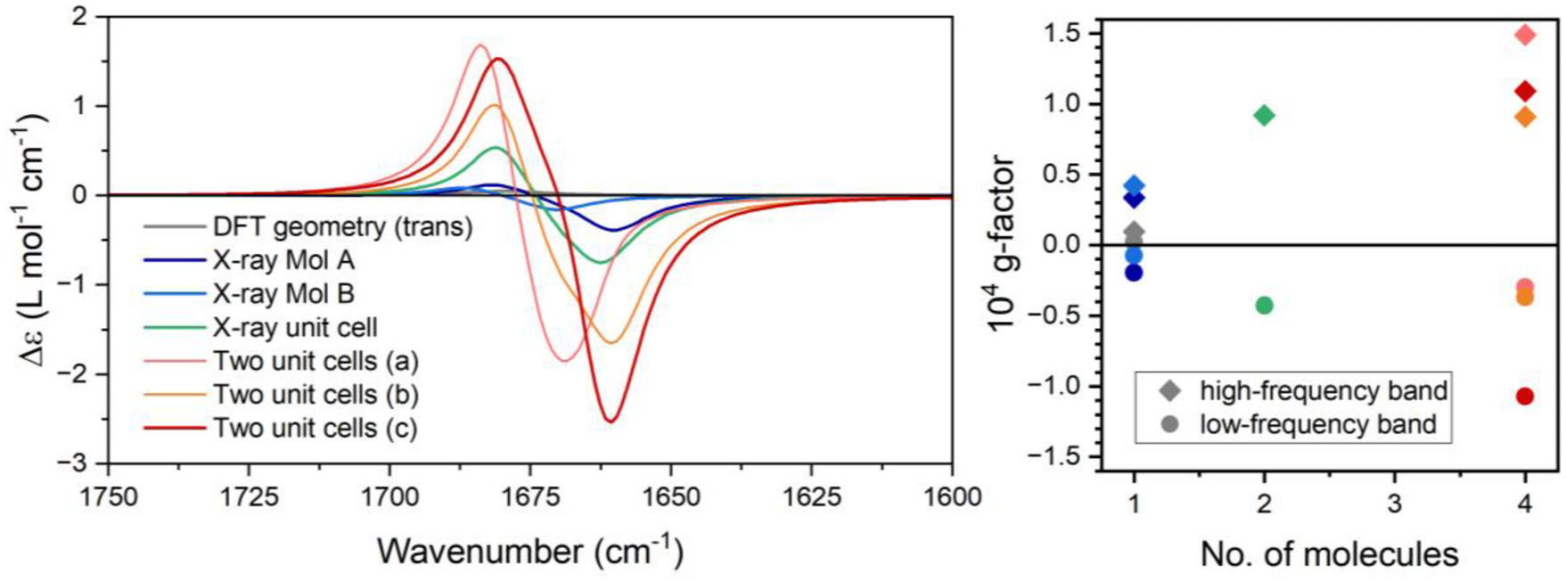
Left: calculated VCD spectra in the symmetric C═O stretching region for the Zn model of *trans*‐*l*‐**3** (fully optimized by DFT) and partially optimized structures extracted from the X‐ray geometry of *l*‐**3**, with Co replaced by Zn in all cases. “Two unit cells (a)” refers to an X‐ray cell duplicate along the (1,0,0) axis, and so on. Calculation level B3LYP(‐D3BJ)/def2‐SVP/PCM, frequency scaling factor 0.97, Lorentzian bandwidth = 8 cm^−1^. Right: calculated *g*‐factors as a function of the number of molecules. Symbol colors correspond to the spectra on the left.

## 3 Conclusions

While previous studies reported “Co(tfc)_2_” species without accounting for ancillary ligands, we demonstrate that cobalt(II) bis(trifluoroacetylcamphorato) complexes are readily accessible as bis‐aqua adducts. However, coordinated water molecules are labile and undergo reversible displacement in coordinating solvents such as acetonitrile and alcohols. The resulting interconversion between different species represents an uncommon case of solvent‐driven ligand switching accompanied by geometry rearrangement. Notably, this pronounced structural tunability appears to be a distinctive feature of tfc ligands, as related cobalt(II) diketonate complexes in the literature do not undergo reversible water‐solvent ligand exchange.

The chiroptical response of complexes **1–3**, spanning the UV, vis, NIR, and IR ranges, is highly sensitive to these environmental effects, as demonstrated by ECD and VCD measurements across multiple solvents and concentration regimes. VCD data reveal two distinct sources of enhancement. In dilute solution, dissymmetry factors up to |*g*| ≈ 4 × 10^−^^3^ are observed, consistent with vibronic coupling between ligand‐centered vibrations and low‐lying electronic states (LLES) typical of high‐spin Co^II^. Above 30 mM in CD_3_CN, microcrystallization of **3** occurs spontaneously, and the resulting suspensions exhibit a dramatic VCD enhancement, reaching *g* ≈ 0.2 at 1600 cm^−^^1^. This value represents the largest dissymmetry factor ever reported by VCD, surpassing previous records for both transition metal complexes and aggregated organic systems. The record value originates mainly from aggregation of **3** into microcrystals, which amplifies its intrinsic signal by nearly two orders of magnitude. The structural identity of the microcrystalline phase responsible for this exceptional enhancement is firmly established by the convergence of VCD profiles, the reversibility of the **1** → **3** conversion, and the crystallographic characterization from analogous concentrated CH_3_CN solutions.

To the best of our knowledge, this work provides the first documented example in which vibronic coupling and aggregation‐induced enhancement concur for the same chemical species. The two contributions may act synergistically rather than additively, as qualitatively supported by calculations, thus yielding dissymmetry factors of unprecedented magnitude. These findings establish Co(tfc)_2_‐based complexes as a versatile platform for chiroptical spectroscopy and open the way to broader exploration of supramolecular architectures based on chiral cobalt(II) diketonate scaffolds as sources of exceptionally intense VCD signals. A potentially wide range of ancillary ligands may provide access to new systems with unique chiroptical properties. Finally, the extraordinary signal intensity enables acquisition of meaningful VCD spectra in a single scan, opening direct prospects for real‐time chiroptical monitoring of dynamic processes.

## Author Contributions

**Gianmarco Papi**: investigation, writing – review and editing, visualization, formal analysis, data curation. **Massimo Guelfi**: investigation, writing – review and editing, formal analysis. **Stefano Zacchini**: investigation, writing – review and editing, visualization, formal analysis. **Fabio Marchetti**: investigation, writing – original draft, writing – review and editing, visualization, data curation, conceptualization. **Gennaro Pescitelli**: investigation, writing – original draft, writing – review and editing, visualization, data curation, conceptualization.

## Conflicts of Interest

The authors declare no conflicts of interest.

## Supporting information



Experimental and computational sections; synthetic details; characterization data including IR and NMR spectra; additional ECD and VCD spectra; additional computational results; additional photographs (PDF). Crystallographic structures (cif) and checkcif file (PDF) of *l*‐**1**, *d*‐**2**, *l*‐**3**, and *d‐* **4**. The authors have cited additional references within the Supporting Information [99, 100, 101, 102].**Supporting File 1**: anie74254‐sup‐0001‐SuppMat.docx.

## Data Availability

The data that supports the findings of this study are available in the supplementary material of this article.
