# Supplementary material for: Record Dissymmetry in Vibrational Optical Activity: Combining Vibronic Coupling and Aggregation in Cobalt(II) Camphorate Complexes
Source: Angew Chem Int Ed Engl. 2026 Aug 20;65(42):e5800931. doi: 10.1002/anie.5800931 (PMC13653589; doi:10.1002/anie.5800931)
Supplement: Supplementary file 1 [file ANIE-65-e5800931-s001.docx]

**Record Dissymmetry in Vibrational Optical Activity: Combining Vibronic Coupling and Aggregation in Cobalt(II) Camphorate Complexes**

Gianmarco Papi,^a^ Massimo Guelfi,^a^ Stefano Zacchini,^b^ Fabio Marchetti,^a,^* and Gennaro Pescitelli^a,^*

^a^ Department of Chemistry and Industrial Chemistry, University of Pisa, Via Giuseppe Moruzzi 13, I-56124 Pisa, Italy

^b^ Department of Industrial Chemistry “Toso Montanari”, University of Bologna, Via Piero Gobetti 85, I-40129 Bologna, Italy

**Contents**

1. Materials and methods	S2
2. Synthesis and characterization of metal complexes	S3
3. X-ray crystallography	S8
4. IR spectra	S20
5. NMR spectra	S22
6. Chiroptical spectra	S27
7. Calculations	S39
8. Photographs	S43
9. Literature data	S46
10. Cartesian coordinates	S47
11. References	S83

**Experimental**

**1. Materials and methods.**

Reagents and solvents were obtained from Merck and TCI Chemicals and were of the highest purity available. All operations were conducted in air at room temperature unless otherwise stated, and all cobalt complexes (except **3**) were stored in air after isolation. For handling moisture sensitive complexes *d*-**3** and *l*-**3**, CH_3_CN was distilled over CaH_2_, CH_2_Cl_2_ was purified using an mBraun MB SPS5 solvent purification system, while CD_3_CN and CDCl_3_ were stored in Rotaflo tubes under N_2_ over molecular sieves as received. For each pair of enantiomers, only one synthetic procedure is reported, as both enantiomers were obtained using an identical method. IR spectra of solid samples were recorded on an Agilent Cary 630 FT-IR spectrometer. The position of the peaks is given in cm^–1^, while relative intensities are expressed with the abbreviations w (weak), m (medium), s (strong) and v (very). IR spectra were processed using Spectragryph software. NMR spectra were recorded at room temperature on a Jeol JNM-ECZ500R instrument equipped with a Royal HFX Broadband probe. Chemical shifts (ppm) are referenced to the residual solvent peaks (^1^H, ^13^C) or to an external standard (^19^F to CFCl_3_).^[1-2]^ The following abbreviations are used to describe NMR resonances: s (singlet), d (doublet), t (triplet), m (multiplet), br (broad). Elemental (CHNS) analyses were performed on a Vario MICRO cube instrument (Elementar). Melting/decomposition temperatures were determined using a Stuart^TM^ SMP10 melting point apparatus (0–300 °C range, ±1 °C). Magnetic susceptibilities (reported per mole of complex) were measured at 298 K on solid samples using a Magway MSB Mk1 magnetic susceptibility balance (Sherwood Scientific Ltd.). Diamagnetic corrections were applied according to König.^[3]^ ECD, NIR-CD and VCD spectra were recorded on Jasco J-1500 (CD) and Jasco FVS-6000 (VCD) spectropolarimeters under the following conditions unless otherwise stated: ECD, 3 mM solutions in dichloromethane, methanol and acetonitrile, using 0.01 cm pathlength cells (180-400 nm) and 1 cm cells (400-820 nm); NIR-CD, 15 mM solutions in dichloromethane and acetonitrile using 1 cm pathlength cells (800-1600 nm); VCD, 15 and 40 mM solutions in CDCl_3_ and CD_3_CN using 200 µm BaF_2_ cells (3200–850 cm^-1^). The use of deuterated solvents is compulsory in IR/VCD measurements to avoid saturation of signals due to solvent C-H bending modes in the diagnostic region. The integrity of the cobalt complexes after the spectroscopic measurements was confirmed by IR spectroscopy and/or elemental analysis of the solid residue obtained after solvent removal under vacuum.

**2.** **Synthesis and characterization of metal complexes**

**2.1. [Co(*d*-tfc)_2_(H_2_O)_2_], *d*-1 (Chart S1)**

**Chart S1**. Structure of *d*-**1**

A pink suspension of Co(NO_3_)_2_·6H_2_O^[1]^ (100 mg, 0.344 mmol) in methanol (10 mL) was prepared in a 100 mL round-bottom flask and treated with *d*-3-(trifluoroacetyl)camphor (*d*-tfcH; 146 µL, 0.689 mmol), followed by Na_2_CO_3_ (73 mg, 0.69 mmol). A turbid orange-red solution formed, which was stirred for an additional 18 h at room temperature. The resulting orange solution was treated with H_2_O (≈ 50 mL), leading to precipitation of a pink powder. The solid was separated and redissolved in MeOH (5 mL), followed by addition of water (≈ 50 mL). A pink solid was isolated, washed with pentane (2 x 10 mL), dried in an oven at 50 - 60 °C, and then under vacuum using a mechanical pump. Yield 135 mg (67%). Anal. calcd. for C_24_H_32_CoF_6_O_6_: C, 48.90; H, 5.47. Found: C, 48.54; H, 5.31. Melting point = 139 ± 1 °C (decomposition). Magnetic measurement: µ*_eff_* = 4.78 BM. IR (solid state): ῦ/cm^-1^ = 3394s (br), 2960m, 2872w, 1636s, 1525s, 1420w, 1326m, 1266m, 1222s, 1223s, 1182s, 1139vs, 1056s, 920m, 806s, 714m. ^19^F NMR (CDCl_3_): δ/ppm: −68.7, −70.0. ^19^F NMR (CD_3_OD): δ/ppm: 12.8. ^19^F NMR (CD_3_CN): δ/ppm = 33.4. Soluble in methanol, ethanol, acetone, acetonitrile, dichloromethane and diethyl ether; insoluble in pentane, hexane, toluene and water. Crystals of *l*-**1** suitable for X-ray analysis were obtained by slow diffusion of pentane into a diethyl solution stored at -30 °C.

**2.2. [Co(*l*-tfc)_2_(H_2_O)_2_], *l*-1 (Chart S2)**

**Chart S2**. Structure of *l*-**1**

From Co(NO_3_)_2_⋅6H_2_O (60 mg, 0.21 mmol), *l*-3-(trifluoroacetyl)camphor (90 µL, 0.42 mmol) and Na_2_CO_3_ (44 mg, 0.42 mmol). Pink solid, yield 78 mg (63%). Anal. calcd. for C_24_H_32_CoF_6_O_6_: C, 48.90; H, 5.47. Found: C, 48.45; H, 5.32. Melting point = 140 ± 1 °C (decomposition).

**2.3. [Co(*d*-tfc)_2_(H_2_O)(OHEt)], *d*-2 (Chart S3)**

**Chart S3**. Structure of *d*-**2**

A solution of *d*-**1** (150 mg, 0.25 mmol) in ethanol (10 mL) was stirred at room temperature overnight. The final red solution was dried under vacuum, and the residue washed with pentane (2 x 10 mL) to afford a dark-red powder. Yield 110 mg (71%). Anal. calcd. for C_26_H_36_CoF_6_O_6_: C, 50.57; H, 5.88. Found: C, 50.03; H, 5.83. Melting point = 136 ± 1 °C (decomposition). Magnetic measurement: µ*_eff_* = 4.15 BM. IR (solid state): ῦ/cm^-1^ = 3246(br), 2961m, 1646s, 1128s, 921m, 807s, 687m. Soluble in ethanol, acetone, dichloromethane; insoluble in pentane, hexane, toluene. Crystals of *d*-**2** suitable for X-ray analysis were obtained by slow evaporation of the solvent from an ethanol solution in air.

**2.4. [Co(*d*-tfc)_2_(CH_3_CN)_2_], *d*-3 (Chart S4)**

**Chart S4**. Structure of *d*-**3**

Complex *d*-**1** (64 mg, 0.11 mmol) was introduced into a Schlenk tube under an N_2_ atmosphere and treated with distilled acetonitrile (ca. 10 mL). The resulting yellow suspension was stirred overnight, undergoing progressive dissolution of the solid to afford a light-yellow solution. The solution was dried under vacuum, and the resulting ochre powder was stored in sealed ampoules under N_2_. Yield 65 mg (92%). Anal. calcd. for C_28_H_34_CoF_6_N_2_O_4_: C, 52.92; H, 5.39; N, 4.41. Found: C, 52.61; H, 5.47; N, 4.16. Melting point = 125 ± 1 °C (decomposition). Magnetic measurement: µ*_eff_* = 4.70 BM. IR (solid state): ῦ/cm^-1^ = 2959w, 2874w, 2277w, 1647s, 1533m, 1269s, 1126s, 921w, 807m, 688w. 2986sh, 2960m-br, 2934m-br, 2874br, 2310vw, 2278w, 1644s, 1623sh, 1565m, 1532s, 1474w, 1453w, 1430br, 1388w, 1376w, 1328m, 1295w, 1267s, 1230sh, 1221s, 1199s, 1188sh, 1178vs, 1125vs, 1105m-s, 1078m-s, 1052m, 1006w-m, 967vw, 941vw, 921m, 892w, 871vw, 849w, 806s, 744sh, 736m, 715m, 687m. ^19^F NMR (CD_3_CN): δ/ppm = 33.6. ^19^F NMR (CDCl_3_): δ/ppm = 11 − 22 (br). X-ray diffraction analysis was performed on light-yellow crystals grown from a concentrated acetonitrile solution (≈ 5 mg/mL) at room temperature. When left in air, *d*-**3** gradually converted into *d*-**1**, as evidenced by a color change from ochre to pink. IR and elemental analyses on a solid sample of *d*-**3** left in air for 3 days confirmed complete conversion into *d*-**1**.

**2.5. [Co(*l*-tfc)_2_(CH_3_CN)_2_], *l*-3 (Chart S5)**

**Chart S5**. Structure of *l*-**3**

From *l*-**1** (31 mg, 0.053 mmol) and acetonitrile (ca. 5 mL). Ochre solid, yield 30 mg (89%). Anal. calcd. for C_28_H_34_CoF_6_N_2_O_4_: C, 52.92; H, 5.39; N, 4.41. Found: C, 52.40; H, 5.47; N, 4.20. Melting point = 125 ± 1 °C (decomposition).

**2.6. [Zn(*d*-tfc)_2_(H_2_O)], *d*-4 (Chart S6)**

**Chart S6**. Structure of *d*-**4**

The title compound was prepared following a procedure similar to that described for *d*-**1**, using ZnCl_2_ (55 mg, 0.40 mmol),^[2]^ *d*-3-(trifluoroacetyl)camphor (*d*-tfcH, 193 μL, 0.910 mmol) and Na_2_CO_3_ (101 mg, 0.953 mmol). The colorless solid obtained was washed with pentane (20 mL) for 10 min and dried under vacuum. Yield 180 mg (78%). Soluble in methanol, ethanol, acetonitrile, acetone, dichloromethane, chloroform, diethyl ether, toluene and hexane; slightly soluble in pentane; insoluble in water. Anal. calcd. for C_24_H_30_F_6_O_5_Zn: C, 49.88; H, 5.23. Found: C, 49.39; H, 5.15. Melting point = 125 ± 1 °C. IR (solid state): ῦ/cm^-1^ = 3300w (br, H_2_O), 2979s, 2967s, 2930sh, 2884m, 2738vw, 2358vw (br), 1654s, 1558s, 1476w, 1452w, 1409m, 1391m,1380m, 1328s, 1294m, 1267s, 1221s, 1197s, 1179s, 1130vs, 1105s, 1078s, 1058s-m, 1007m, 922m, 890w, 873w, 851w, 808s, 745sh, 736m, 712m, 688m. ^1^H NMR (CDCl_3_): δ/ppm = 2.86 (m, 1H, CH); 2.04 (m, 1H, CH_2_); 1.75 (m, 1H, CH_2_); 1.61 (s-br, 2H, H_2_O);1.44 (m, 2H, CH_2_); 1.00 (s, 3H, CH_3_); 0.92 (s, 3 H, CH_3_); 0.87 (s, 3 H, CH_3_). ^13^C NMR (CDCl_3_): δ/ppm = 215.8 (CO), 163.1 (CO); 120.6 (q, ^1^*J*_CF_ = 282 Hz, CF_3_), 114.0 (C), 59.4 (C), 50.6 (C), 48.2 (CH), 30.7 (CH_2_), 27.2 (CH_2_), 20.4 (CH_3_), 19.0 (CH_3_), 9.1 (CH_3_). ^19^F NMR (CDCl_3_): δ/ppm = −70.9. Crystals suitable for X-ray analysis were obtained from a concentrated pentane solution settled aside at –30 °C for seven days.

**3. X-ray Crystallography**

Single-crystal X-ray diffraction (SC-XRD) measurements for compounds *d*-**2**, *l*-**3**, and **4** were performed using a Bruker D8 Venture diffractometer equipped with a microfocus Mo X-ray source (Kα radiation, λ = 0.71073 Å) and a Photon III 2D detector. Data for compound *l*-**1** were recorded on a Bruker APEX II diffractometer equipped with a PHOTON2 detector using Mo–Kα radiation. Crystallographic and data collection details for compounds *l*-**1**, *d*-**2**, *l*-**3**, and *d-***4** are summarized in Table S1. Unit cell parameters were determined and initially refined using APEX4.^[4]^ Data integration and reduction were carried out with SAINT^[5]^ and XPREP,^[6]^ while absorption corrections were applied using SADABS.^[7]^ All structures were solved and refined by full-matrix least-squares methods on F^2^ using SHELXL-2019/1.^[8]^ Hydrogen atoms were placed in calculated positions and refined using a riding model, except for H1A, H1B, H1D, H1E and H1C, H1F belonging respectively to the water and ethanol molecules coordinated to Co1 and Co2 in *d*-**2**, and for H1G and H1H belonging to the lattice ethanol molecules. These hydrogen atoms were located from difference Fourier maps and freely refined with respect to the corresponding oxygen atoms. The same procedure was applied to hydrogen atoms HwD and HwB belonging to the two coordinated water molecules in complex *d-***4**. All non-hydrogen atoms were refined with anisotropic displacement parameters. Significant positional disorder was observed for the fluorine atoms of the CF_3_ group of the *tfc* ligand in complexes *d*-**2** and *l*-**3**, and the electron density associated with these atoms was therefore modelled over multiple positions. The occupancies of all disordered atoms were freely refined using FVAR instructions. To avoid non-physical anisotropic displacement parameters, EADP constraints were applied to selected carbon and fluorine atoms. In addition, DFIX and DANG restraints were employed to optimize the geometry of the coordinated water molecules. Crystallographic data for compounds *l*-**1**, *d*-**2**, *l*-**3** and *d-***4** have been deposited with the Cambridge Crystallographic Data Centre (CCDC) under deposition numbers 2546771 (*l*-**1**), 2546772 (*d*-**2**), 2546773 (*l*-**3**) and 2546774 (*d-***4**).

**Table S1.** Crystal data, collection parameters and structure refinement for complexes *l*-**1**, *d*-**2,** *l*-**3**, and *d*-**4**

|  | ***l-*1** | ***d*-2** | ***l*-3** | ***d*-4** |
| --- | --- | --- | --- | --- |
| CCDC ID | 2546771 | 2546772 | 2546773 | 2546774 |
| Formula | C_24_H_32_CoF_6_O_6_ | C_28_H_42_CoF_6_O_7_ | C_28_H_34_CoF_6_N_2_O_4_ | C_24_H_30_F_6_O_5_Zn |
| FW, g mol^-1^ | 589.42 | 663.55 | 635.50 | 577.85 |
| T, K | 100(2) | 296(2) | 298(2) | 100(2) |
| *λ*, Å | 0.71073 | 0.71073 | 0.71073 | 0.71073 |
| Crystal system | Monoclinic | triclinic | triclinic | triclinic |
| Space group | *C*2 | *P1* | *P1* | *P1* |
| *a*, Å | 23.8320(19) | 10.0989(6) | 7.4043(6) | 9.4950(2) |
| *b,* Å | 12.5499(11) | 13.6205(9) | 14.9202(10) | 10.9283(3) |
| *c*, Å | 9.4291(8) | 13.6587(9) | 15.6088(11) | 12.9517(4) |
| *α, °* | 90 | 61.303(2) | 63.347(2) | 77.493(1) |
| *β,°* | 102.885(3) | 81.386(2) | 88.040(3) | 79.733(1) |
| *γ, °* | 90 | 85.896(2) | 88.908(3) | 80.453(1) |
| Cell Volume, Å^3^ | 2749.1(4) | 1629.42(18) | 1540.2(2) | 1279.64(6) |
| Z | 4 | 2 | 2 | 2 |
| *D_c_*, g∙cm^-3^ | 1.424 | 1.352 | 1.370 | 1.500 |
| μ, mm^−1^ | 0.700 | 0.601 | 0.627 | 1.036 |
| F(000) | 1220 | 694 | 658 | 596 |
| Crystal size, mm | 0.15x0.12x0.09 | 0.42x0.33x0.08 | 0.30x0.06x0.03 | 0.17x0.08x0.02 |
| θ limits,° | 1.75 to 25.20 | 2.49 to 28.28 | 2.54 to 23.01 | 1.93 to 27.14 |
| Reflections collected | 10473 | 82613 | 33158 | 75244 |
| Independent reflections | 4943 [R(int) = 0.0419] | 15903 [R(int) = 0.0431] | 8578 [R(int) = 0.0922] | 11202 [R(int) = 0.0481] |
| Data / restraints  / parameters | 4943 / 5 / 354 | 15903/8/838 | 8578/3/830 | 11202/7/672 |
| Goodness on fit on F^2 a^ | 1.033 | 1.031 | 1.010 | 1.057 |
| *R*_1_ (*I* > 2σ(*I*)) ^b^ | 0.0358 | 0.0365 | 0.0454 | 0.0265 |
| Absolute structure  parameter | 0.03(3) | 0.048(12) | 0.034(16) | 0.017(8) |
| *wR*_2_ (all data) ^c^ | 0.0880 | 0.0962 | 0.0938 | 0.0625 |
| Largest diff. peak and hole, e Å^-3^ | 0.486 and  -0.299 | 0.401 and  -0.295 | 0.176 and  -0.200 | 0.437 and  -0.292 |

**3.1 Single-crystal X-ray structure of [Co(tfc)_2_(H_2_O)_2_] (*l*-1)**

**Table S2**. Bond length and angles around the metal center for complexes *l*-**1**

| Co1-O1 | 2.069(3) | Co1-O2 | 2.099(4) | Co1-O3 | 2.090(5) |
| --- | --- | --- | --- | --- | --- |
| Co2-O11 | 2.085(3) | Co2-O12 | 2.090(5) | Co2-O13 | 2.075(4) |
| O1-Co1-O2 | 89.17(15) | O1-Co1-O3 | 91.12(16) | O1-Co1-O2* | 86.66(16) |
| O1-Co1-O3* | 93.68(16) | O2-Co1-O3 | 87.73(16) | O2-Co1-O2* | 100.0(3) |
| O3-Co1-O3* | 84.6(2) |  |  |  |  |
| O11-Co2-O12 | 89.37(15) | O11-Co2-O13 | 94.02(16) | O11-Co2-O12* | 86.40(16) |
| O11-Co2-O13* | 90.70(16) | O12-Co2-O13 | 91.41(16) | O12-Co2-O12* | 95.1(3) |
| O13-Co2-O13* | 82.2(2) |  |  |  |  |

**Table S3.** Hydrogen bonds for *l*-**1** [Å and °]

| **D-H···A** | **d(D-H)** | **d(H···A)** | **d(D···A)** | **<(DHA)** |
| --- | --- | --- | --- | --- |
| O(3)-H(1)**···**O(11)#1 | 0.83(3) | 2.18(5) | 2.792(6) | 131(5) |
| O(3)-H(1)**···**F(13)#1 | 0.83(3) | 2.41(5) | 3.020(5) | 131(5) |
| O(3)-H(2)**···**O(12)#2 | 0.83(3) | 2.09(4) | 2.791(6) | 143(6) |
| O(13)-H(21)**···**O(1) | 0.86(3) | 1.90(3) | 2.714(6) | 158(5) |
| O(13)-H(21)**···**F(3) | 0.86(3) | 2.40(5) | 3.008(5) | 128(5) |
| O(13)-H(22)**···**O(2)#1 | 0.86(3) | 1.98(4) | 2.776(6) | 154(6) |

Symmetry transformations used to generate equivalent atoms: #1 -x+1, y, -z; #2 x, y, z-1.

**Figure S1.** View of the infinite chain parallel to the crystallographic *c* axis of H-bonded *l*-**1** complexes within its solid-state structure. H-bonds are represented as blue dashed lines.

**3.2 Single-crystal X-ray structure of [Co(tfc)_2_(H_2_O)(EtOH)]·EtOH (*d*-2·EtOH)**

**Figure S2.** Molecular structure of *d*-**2** with key atoms labelled, showing the two independent molecules within the unit cell with opposite chirality at metal. Displacement ellipsoids are drawn at the 30% probability level. Hydrogen atoms are omitted for clarity. The asymmetric unit contains two crystallographically independent complexes and two lattice ethanol molecules, consistent with the triclinic *P*1 space group. Both molecules exhibit opposite metal-centered helicities and contain enantiopure *d*-tfc ligands with absolute configurations *R* for C10, C11, C36 and C54, and *S* for C13, C18, C28 and C44. Color code: Co (blue), O (red), C (grey), F (yellow-green).

**Table S4**. Bond length and angles around the metal center for complexes *d*-**2**

| Co1-O1 | 2.039(3) | Co1-O2 | 2.073(3) | Co1-O3 | 2.046(3) | Co1-O4 | 2.082(3) |
| --- | --- | --- | --- | --- | --- | --- | --- |
| Co1-O5 | 2.184(3) | Co1-O6 | 2.050(4) | Co2-O7 | 2.041(3) | Co2-O8 | 2.079(3) |
| Co2-O9 | 2.050(3) | Co2-O10 | 2.083(3) | Co2-O11 | 2.168(3) | Co2-O12 | 2.075(3) |
| O1-Co1-O2 | 90.74(12) | O1-Co1-O3 | 92.30(13) | O3-Co1-O2 | 95.32(14) | O3-Co1-O4 | 88.18(12) |
| O2-Co1-O4 | 85.56(13) | O1-Co1-O5 | 86.25(12) | O1-Co1-O6 | 94.66(15) | O6-Co1-O2 | 90.54(16) |
| O3-Co1-O5 | 84.44(12) | O6-Co1-O4 | 85.26(14) | O4-Co1-O5 | 97.46(12) | O6-Co1-O5 | 90.08(15) |
| O7-Co2-O8 | 91.41(12) | O7-Co2-O9 | 91.45(13) | O9-Co2-O8 | 90.10(13) | O9-Co2-O10 | 89.08(12) |
| O7-Co2-O11 | 85.93(13) | O7-Co2-O12 | 92.51(15) | O12-Co2-O8 | 93.71(15) | O12-Co2-O10 | 86.98(14) |
| O8-Co2-O10 | 88.39(13) | O9-Co2-O11 | 86.41(12) | O10-Co2-O11 | 94.31(13) | O12-Co2-O11 | 89.98(14) |

**Table S5**. Hydrogen bonds for *d*-**2** [Å and °]. The interactions are classified as hydrogen bonds between coordinated water molecules and tfc ligands (Wcoord–tfc), between lattice ethanol and coordinated water (EtOHret–Wcoord), and between coordinated and lattice ethanol molecules (EtOHret–EtOHcoord).

| Interaction type | D-H**···**A | d(D-H) | d(H**···**A) | d(D**···**A) | <(DHA) |
| --- | --- | --- | --- | --- | --- |
| W_coord_-tfc | O5-H1A**···**O9 | 0.910 | 1.837 | 2.741 | 171.59 |
|  | O5-H1B**···**O7 | 0.712 | 2.335 | 2.896 | 136.83 |
| EtOH_ret_-W_coord_ | O14-H1H**···**O5 | 0.891 | 2.080 | 2.880 | 148.82 |
| EtOH_ret_-EtOH_coord_ | O12-H1F**···**O14 | 0.947 | 1.772 | 2.665 | 155.88 |
| W_coord_-tfc | O11-H1D**···**O1 | 0.866 | 2.010 | 2.836 | 159.02 |
|  | O11-H1E**···**O3 | 0.807 | 2.085 | 2.779 | 143.92 |
| EtOH_ret_-W_coord_ | O13-H1G**···**O11 | 0.781 | 2.080 | 2.852 | 170.34 |
| EtOH_ret_-EtOH_coord_ | O6-H1C**···**O13 | 0.716 | 1.988 | 2.684 | 163.98 |

**3.3 Single-crystal X-ray structure of [Co(tfc)_2_(CH_3_CN)_2_] (*d*-3)**

**Table S6**. Bond length and angles around the metal center for complexes *l*-**3**

| Co1-O1 | 2.015(6) | Co1-O2 | 2.063(5) | Co1-O3 | 2.028(6) | Co1-O4 | 2.058(5) |
| --- | --- | --- | --- | --- | --- | --- | --- |
| Co1-N1 | 2.181(12) | Co1-N2 | 2.190(13) | Co2-O5 | 2.056(6) | Co2-O6 | 2.028(7) |
| Co2-O7 | 2.029(7) | Co2-O8 | 2.069(7) | Co2-N3 | 2.161(11) | Co2-N4 | 2.183(11) |
| O1-Co1-O2 | 90.0(2) | O1-Co1-O4 | 90.5(2) | O3-Co1-O4 | 90.1(2) | O3-Co1-O2 | 89.5(2) |
| O1-Co1-N1 | 92.9(3) | O1-Co1-N2 | 86.9(4) | O3-Co1-N1 | 86.3(3) | O3-Co1-N2 | 93.9(3) |
| O2-Co1-N1 | 91.5(3) | O4-Co1-N1 | 91.3(3) | O2-Co1-N2 | 88.4(3) | O4-Co1-N2 | 88.8(3) |
| O6-Co2-O5 | 90.7(3) | O7-Co2-O5 | 87.5(3) | O6-Co2-O8 | 91.2(3) | O7-Co2-O8 | 90.6(3) |
| O6-Co2-N3 | 90.2(3) | O7-Co2-N3 | 89.2(3) | O5-Co2-N3 | 93.5(3) | O8-Co2-N3 | 88.8(3) |
| O6-Co2-N4 | 89.6(3) | O5-Co2-N4 | 86.3(3) | O8-Co2-N4 | 91.4(4) | O7-Co2-N4 | 91.0(3) |

**Table S7**. Intermolecular O···H and F···H contacts identified for the two independent units (Co1 and Co2) in the asymmetric unit of *l*-**3**. Contacts are grouped according to the functional group involved and the cobalt center (Co1 or Co2). Distances were calculated using Mercury and include only intermolecular interactions shorter than the sum of the van der Waals radii (up to 2.80 Å below the VdW limit) [Å and °].

| Interaction type | | D-H**···**A | d(D-H) | d(H**···**A) | d(D**···**A) | <(DHA) |
| --- | --- | --- | --- | --- | --- | --- |
| O···H | | | | | | |
| Co1···Co1 | CH_3_CN**···**tfc | C53-H53A**···**O1 | 0.961 | 2.443 | 3.397 | 172.06 |
|  | CH_3_CN**···**tfc | C23-H23C**···**O2 | 0.960 | 2.611 | 3.562 | 170.60 |
| Co2···Co2 | CH_3_CN**···**tfc | C47-H47B**···**O6 | 0.961 | 2.693 | 3.575 | 152.97 |
|  | CH_3_CN**···**tfc | C55-H55A**···**O7 | 0.960 | 2.494 | 3.450 | 173.81 |
| F···H | | | | | | |
| Co1···Co2 | tfc**···**tfc | C16-H16B**···**F8 | 0.960 | 2.613 | 3.461 | 147.37 |
| Co1···Co1 | CH_3_CN**···**tfc | C23-H23C**···**F4B | 0.960 | 2.596 | 3.460 | 149.93 |
| Co2···Co1 | CH_3_CN**···**tfc | C47-H47C**···**F5B | 0.960 | 2.265 | 3.204 | 165.66 |
| Co2···Co1 | tfc**···**tfc | C38-H38A**···**F1B | 0.970 | 2.652 | 3.257 | 120.78 |

**Figure S3.** Intermolecular O···H contacts involving the Co1 unit in *l*-**3**. Interactions are shown as green dashed lines; neighboring molecules are highlighted in yellow. Displacement ellipsoids are drawn at the 30% probability level and hydrogen atoms are omitted for clarity.

**Figure S4.** Intermolecular O···H contacts involving the Co2 unit in *l*-**3**. Interactions are shown as green dashed lines; neighboring molecules are highlighted in yellow. Displacement ellipsoids are drawn at the 30% probability level and hydrogen atoms are omitted for clarity.

**Figure S5.** Intermolecular H···F contacts in *l*-**3**, shown as green dashed lines. Neighboring molecules are highlighted in pink (Co1) and blue (Co2) for clarity. Displacement ellipsoids are drawn at the 30% probability level and hydrogen atoms are omitted for clarity.

**Figure S6.** Crystal packing of *l*-**3** viewed along the crystallographic *a*-axis. The arrangement highlights the regular distribution of the molecular units within the lattice. Displacement ellipsoids are drawn at the 30% probability level, and hydrogen atoms are omitted for clarity.

**Figure S7.** Crystal packing of *l*-**3** viewed along the crystallographic *b*-axis. The arrangement highlights the regular distribution of the molecular units within the lattice. Displacement ellipsoids are drawn at the 30% probability level, and hydrogen atoms are omitted for clarity.

**Table S8.** Quantification of the degree of planarity of each six-membered ring defining the coordination of the *tfc* ligand in *l*-**3** expressed by the MPP parameter.

| Mean plane | Distances *d_i_* (Å) | MPP  $\sqrt{\frac{1}{N}\sum_{i} d_{i}^{2}}$ |
| --- | --- | --- |
|  |  | 0.126 |
|  |  | 0.116 |
|  |  | 0.095 |
|  |  | 0.018 |

**3.4 Single-crystal X-ray structure of [Zn(tfc)_2_(H_2_O)] (*d*-4)**

**Figure S8**. Molecular structure of *d-***4** with key atoms labelled, showing the two independent molecules within the unit cell. Displacement ellipsoids are drawn at the 30% probability level. Hydrogen atoms are omitted for clarity. The compound contains enantiopure *d*-tfc ligands with absolute configurations *R* for C7 and C18, and *S* for C1 and C20. Color code: Zn (light blue), O (red), C (grey), F (yellow-green).

**Table S9**. Bond length and angles around the metal center for *d*-**4**

| Zn1-O1 | 2.015(3) | Zn1-O2 | 2.040(3) | Zn1-O3 | 2.052(3) | Zn1-O4 | 2.026(3) |
| --- | --- | --- | --- | --- | --- | --- | --- |
| Zn1-O5 | 2.005(3) | Zn2-O6 | 2.037(3) | Zn2-O7 | 2.016(3) | Zn2-O8 | 2.020(3) |
| Zn2-O9 | 2.028(3) | Zn2-O10 | 2.012(3) |  |  |  |  |
| O1-Zn1-O2 | 90.91(11) | O1-Zn1-O3 | 87.35(11) | O1-Zn1-O4 | 147.42(11) | O5-Zn1-O1 | 102.51(13) |
| O2-Zn1-O3 | 166.28(10) | O4-Zn1-O2 | 84.61(11) | O4-Zn1-O3 | 89.54(10) | O5-Zn1-O2 | 98.39(12) |
| O5-Zn1-O3 | 95.27(12) | O5-Zn1-O4 | 110.07(13) | O7-Zn2-O6 | 90.26(11) | O7-Zn2-O8 | 154.51(11) |
| O7-Zn2-O9 | 82.53(11) | O8-Zn2-O6 | 88.88(11) | O9-Zn2-O6 | 159.53(10) | O10-Zn2-O9 | 104.28(11) |
| O10-Zn2-O7 | 106.55(12) | O10-Zn2-O8 | 98.87(12) | O10-Zn2-O6 | 96.12(11) | O8-Zn2-O9 | 89.51(11) |

**4. IR spectra**

**Figure S9**. Solid-state IR spectrum (4000-650 cm^-1^) of *d*-**1**

**Figure S10**. Solid-state IR spectrum (4000-650 cm^-1^) of *d*-**2**

**Figure S11**. Solid-state IR spectrum (4000-650 cm^-1^) of *d*-**3**

**Figure S12**. Solid-state IR spectrum (4000-650 cm^-1^) of *d-***4**

**5. NMR spectra**

**Figure S13**. ^19^F NMR spectrum (376 MHz, CDCl_3_) of *d*-**1**

**Figure S14**. ^19^F NMR spectrum (376 MHz, CD_3_OD) of *d*-**1**

**Figure S15**. ^19^F NMR spectrum (376 MHz, CD_3_CN) of *d*-**1**

**Figure S16**. ^19^F NMR spectrum (376 MHz, CD_3_CN) of *l*-**3**

**Figure S17**. ^19^F NMR spectrum (376 MHz, CDCl_3_) of *d-***3**

**Figure S18**. ^1^H NMR spectrum (400 MHz, CDCl_3_) of *d-***4**

**Figure S19**. ^13^C{^1^H} NMR spectrum (101 MHz, CDCl_3_) of *d-***4**

**Figure S20**. ^19^F NMR spectrum (376 MHz, CDCl_3_) of *d-***4**

**6. Chiroptical spectra**

**6.1 Absorption and ECD spectra in the UV, vis and NIR**

**Figure S21**. Optical (bottom) and chiroptical (top) spectra of *l*-**1** (red curves) and *d*-**1** (blue curves) in methanol.

**Figure S22**. Optical (bottom) and chiroptical (top) superspectrum of *l*-**3** (red curves) and *d*-**3** (blue curves) in the UV, vis and NIR regions (from left to right), in CH_3_CN solutions. The deleted portions in the NIR region correspond to spectral areas masked by solvent absorption.

**Figure S23**. Optical (bottom) and chiroptical (top) superspectrum of *l*-**3** (red curves) and *d*-**3** (blue curves) in the UV, vis and NIR regions (from left to right), in CH_2_Cl_2_ solutions. The deleted portions in the NIR region correspond to spectral areas masked by solvent absorption.

**Figure S24**. Optical (bottom) and chiroptical (top) spectra of *d*-**2** in the UV and vis regions (from left to right), in CH_2_Cl_2_ solutions.

**Figure S25**. Optical (bottom) and chiroptical (top) spectra of *d*-**4** in CH_2_Cl_2_ solutions.

**6.2 IR and VCD spectra in solution**

**Figure S26**. IR (bottom) and VCD (top) of *l*-**3** (red curves) and *d*-**3** (blue curves), measured on 20 mM solutions in CD_3_CN (a) and CDCl_3_ (b), cell path-length 200 μm.

**Figure S27**. IR (bottom), VCD (middle) and *g*-value traces (top) of *d*-**4**, measured on 15 mM solutions in CD_3_CN, cell path-length 200 μm.

**Figure S28**. IR (bottom) and VCD spectra (top) of *d*-**1** in the 3000-4000 cm^–1^ region in 30 mM CDCl_3_; cell path-length 200 μm.

**Figure S29**. IR (bottom) and VCD spectra (top) of *d*-**1** (blue traces) and *l*-**1** (red traces) in 40 mM CD_3_CN, 45’ after sample preparation; cell path-length 200 μm.

**Figure S30**. Time evolution of IR (bottom) and VCD spectra (top) of *d*-**1** as solid sample deposited on VCD cell walls recorded in open air. The cell originally contained a 40 mM CD_3_CN solution (“sol”) appearing as a microcrystal suspension.

**Figure S31**. IR (bottom), VCD spectra (middle) and *g*-value trace (top) of *d*-**1** as microcrystal suspension in 40 mM CDCl_3_, cell path-length 200 μm.

**Figure S32**. IR (bottom), VCD spectra (middle) and *g*-value trace (top) of *d*-**3** as microcrystal suspension in 40 mM CD_3_CN, cell path-length 200 μm.

**Figure S33**. VCD spectra of *d*-**1** as suspended microcrystals obtained from 40 mM solution in CD_3_CN, cell path-length 200 μm, with a variable number of accumulations ranging from 1 to 1,000. The noise trace is offset to Y = 0.05 and shown in green.

**7. Calculations**

**7.1 Computational procedure**

All calculations were run with Gaussian 16 program,^[9]^ using default grids and convergence criteria. The X-ray structures of *l*-**1** and *l*-**3** were used as starting geometries. First, the position of H atoms was refined by DFT geometry optimizations run at B3LYP-D3/6-31G(d) level. These partially optimized structures were manipulated to generate other starting structures with different Δ/Λ chirality at metal, *cis*/*trans* coordination geometry and *syn*/*anti* ligand orientation. All structures were then optimized at B3LYP-D3BJ/def2-SVP levels of calculation, either in vacuo or including IEF-PCM solvent model for acetonitrile, dichloromethane and chloroform, and frequency calculations were run at the same levels. TD-DFT calculations were run using B3LYP, CAM-B3LYP and M06-L functionals together with def2-SVP basis set on all atoms or def2-TZVP basis sets on all atoms except Co plus LanL2DZ on Co with the relative ECP. Up to 120 excited states (roots) were included. TD-DFT calculations were run in vacuo or including IEF-PCM solvent model for acetonitrile. Zn models of **3** were built for geometry-optimized Co complexes by replacing Co with Zn without re-optimization. Zn models aimed to reproduce the crystal packing of *l*-**3** were built by replacing Co with Zn and reoptimizing H atoms B3LYP-D3BJ/def2-SVP level. All calculations were on neutral species with multiplicity 4 (3 unpaired electrons, quartet) for Co complexes and multiplicity 1 (0 unpaired electrons) for Zn complexes. The use of multiplicity 2 for Co complexes led to non-stable wavefunction. All spectra were plotted using the software SpecDis. ^[10]^ For the plot in Figure 8 (main text) the following parameters were employed: UV range: exponential band width σ 0.2 eV, wavelength shift Δλ +50 nm, scaling factor sf 1; vis: σ 0.2, Δλ +50 nm, sf 10; NIR: σ 0.1, Δλ -250 nm, sf 5.

**7.2 Calculation results**

**Table S10**. DFT-optimized structures of *l*-**1** with different Δ/Λ chirality at metal, *cis*/*trans* coordination geometry and *syn*/*anti* ligand orientation. Level of calculation B3LYP-D3BJ/def2-SVP, either in gas phase or including IEF-PCM solvent model for acetonitrile and dichloromethane. Structures are shown below for the most populated isomers.

|  |  |  |  |  | Relative internal energy, kcal/mol (population at 300K, %) | | |
| --- | --- | --- | --- | --- | --- | --- | --- |
| ID | Chirality  at metal | Coordination geometry | Ligand orientation | Dipole moment (Debye) | Gas | CH_3_CN | CH_2_Cl_2_ |
| 1 | Λ | *Cis* | *Anti* | 3.22 | 0 (52) | 0.46 (25) | 0.15 (33) |
| 2 | Δ | *Cis* | *Syn* | 4.15 | 0.70 (16) | 0 (54) | 0 (42) |
| 3 | Δ | *Cis* | *Anti* | 3.42 | 0.86 (12) | 1.12 (8) | 0.86 (10) |
| 4 | Λ | *Cis* | *Syn* | 3.99 | 0.81 (13) | 1.24 (7) | 0.93 (9) |
| 5 | Λ | *Cis* | *Syn* | 5.44 | 1.81 (2) | 1.40 (5) | 1.26 (5) |
| 6 | Λ | *Cis* | *Anti* | 1.92 | 1.74 (3) | 2.06 (2) | 1.79(2) |
| 7 |  | *Trans* | *Syn* | 4.77 | 8.26 (0) | 4.64 (0) | 4.98 (0) |
| 8 |  | *Trans* | *Anti* | 1.58 | 8.79 (0) | 4.77 (0) | 5.20 (0) |

|  |  |
| --- | --- |
| 1 | 2 |
|  |  |
| 3 | 4 |

**Table S11**. DFT-optimized structures of *l*-**3** with different Δ/Λ chirality at metal, *cis*/*trans* coordination geometry and *syn*/*anti* ligand orientation. Level of calculation B3LYP-D3BJ/def2-SVP, including IEF-PCM solvent model for acetonitrile. Structures are shown below for the most populated isomers.

| ID | Chirality  at metal | Coordination geometry | Ligand orientation | Dipole moment, D | Relative free energies, kcal/mol (population at 300K, %) |
| --- | --- | --- | --- | --- | --- |
| 1 | Λ | *Cis* | *Anti* | 7.87 | 0 (40) |
| 2 | Λ | *Cis* | *Syn* | 12.4 | 0.36 (22) |
| 3 | Λ | *Cis* | *Anti* | 14.0 | 0.66 (13) |
| 4 | Δ | *Cis* | *Syn* | 12.5 | 1.05 (7) |
| 5 | Δ | *Cis* | *Anti* | 7.50 | 1.20 (5) |
| 6 | Δ | *Cis* | *Anti* | 14.3 | 1.20 (5) |
| 7 | Λ | *Cis* | *Syn* | 12.4 | 1.26 (5) |
| 8 |  | *Trans* | *Syn* | 4.19 | 4.07 (0) |
| 9 |  | *Trans* | *Anti* | 0.54 | 4.70 (0) |

|  |  |
| --- | --- |
| 1 | 2 |
|  |  |
| 3 | 4 |

**Figure S34**. ECD spectra calculated for the *trans* and *cis* isomer of Zn models of *l*-**3** at (a) CAM-B3LYP/def2-TZVP and (b) B3LYP/def2-TZVP level, including PCM for CH_3_CN. For the structures, see Figure 7 in the main text (with Zn atoms replacing Co atoms). Gaussian exponential bandwidth 0.2 eV, no shift or scaling applied.

**Figure S35.** Displacement vectors for the normal modes contributing to the IR and VCD bands ≈ 1520-1540 ^−1^ for the *trans* isomer of *l*-**3**.

**8. Photographs**

**Figure S36.** Photographs of solid samples of **1** (top) and **3** (bottom), kept under inter atmosphere.

**Figure S37.** Optical microscopy images of microcrystals of **1** in bright field (left), under linearly polarized light (middle) and under cross-polarized filters (right).

**Figure S38.** Optical microscopy images of different portions of microcrystals of **1** in bright field.

**9. Literature data**

**Table S12**. Selected literature data on aggregated systems with large VCD enhancement.

| Family | System | Maximum *g*-value | Reference |
| --- | --- | --- | --- |
| Peptide/protein fibrils | Lys(Glu)_10_Lys | 4×10^–2^ | ^[11]^ |
| Oligopeptide hydrogels | GlyAlaGly | 7×10^–3^ | ^[12]^ |
| Crystals | l-guanosine | (7.3 ± 2)×10^−2^ | ^[13]^ |
| Organogels | *N*,*N*’-diperfluoroheptanoyl- 1,2-diaminocyclohexane | 5×10^–2^ | ^[14]^ |

**10. Cartesian coordinates**

```
l-1_ID1
Gibbs energy -3365.23547100 h, no imaginary frequency
Symbol	       X	             Y	             Z
Co 	     -0.025708	      1.912055	     -0.570232
O  	      0.371882	      3.393674	     -2.057334
O  	     -0.309413	      3.747170	      0.707508
O  	      0.229505	      1.093482	      1.295324
O  	      2.064904	      1.778506	     -0.837591
O  	     -0.147438	      0.189499	     -1.629125
O  	     -2.039895	      1.944178	     -0.202383
C  	      1.094834	      0.175503	      1.491205
C  	      2.230215	     -0.054749	      0.739885
C  	      2.632009	      0.765776	     -0.366209
C  	      0.809559	     -0.753196	      2.684740
C  	      3.898355	      0.143491	     -0.934553
C  	      4.559600	     -0.416257	      0.375732
C  	      3.262605	     -1.169196	      0.815012
H  	      3.327735	     -1.691075	      1.776750
C  	      2.973986	     -2.083625	     -0.404989
H  	      1.923936	     -2.399763	     -0.418077
H  	      3.591996	     -2.992991	     -0.381057
C  	      3.342148	     -1.165866	     -1.603365
H  	      2.477029	     -0.942398	     -2.241655
H  	      4.121605	     -1.605766	     -2.243151
C  	      4.990060	      0.676918	      1.360757
H  	      5.322828	      0.225159	      2.308874
H  	      4.178544	      1.379613	      1.597039
H  	      5.835003	      1.256548	      0.956339
C  	      5.763701	     -1.329048	      0.124961
H  	      6.629304	     -0.739247	     -0.215752
H  	      5.578959	     -2.107673	     -0.625708
H  	      6.056506	     -1.830969	      1.061369
C  	      4.703639	      1.041515	     -1.847826
H  	      4.092622	      1.364211	     -2.704889
H  	      5.587166	      0.514946	     -2.239790
H  	      5.044945	      1.947731	     -1.323911
C  	     -1.060304	     -0.653606	     -1.376964
C  	     -2.231077	     -0.432233	     -0.661563
C  	     -2.623492	      0.829434	     -0.125902
C  	     -0.808408	     -2.073289	     -1.921310
C  	     -3.904513	      0.598339	      0.661249
C  	     -4.572524	     -0.532586	     -0.199661
C  	     -3.288217	     -1.420648	     -0.185366
H  	     -3.358483	     -2.352701	     -0.757436
C  	     -3.022710	     -1.606286	      1.333099
H  	     -3.657934	     -2.396206	      1.759790
H  	     -1.978568	     -1.887882	      1.517467
C  	     -3.379844	     -0.206571	      1.905297
H  	     -2.514082	      0.295451	      2.358318
H  	     -4.170343	     -0.255711	      2.668944
C  	     -4.696375	      1.845615	      0.988383
H  	     -4.088850	      2.543794	      1.584946
H  	     -5.596560	      1.598039	      1.571430
H  	     -5.008636	      2.376360	      0.076022
C  	     -4.979092	     -0.075314	     -1.605349
H  	     -5.321555	     -0.936812	     -2.200520
H  	     -4.153003	      0.398035	     -2.154137
H  	     -5.811910	      0.644029	     -1.556234
C  	     -5.794491	     -1.178855	      0.460137
H  	     -6.650857	     -0.486305	      0.445329
H  	     -5.629005	     -1.476702	      1.503195
H  	     -6.091644	     -2.079719	     -0.100767
F  	      0.619449	     -2.021231	      2.264709
F  	     -0.283374	     -0.382822	      3.355665
F  	      1.834279	     -0.771345	      3.557744
F  	      0.303146	     -2.138323	     -2.654682
F  	     -0.676952	     -2.951202	     -0.903886
F  	     -1.827726	     -2.500348	     -2.688763
H  	      1.306293	      3.141621	     -2.174300
H  	      0.399084	      4.200512	     -1.518295
H  	      0.039416	      3.303870	      1.499737
H  	     -1.261173	      3.530282	      0.733702

l-1_ID2
Gibbs energy -3365.23518300 h, no imaginary frequency
Symbol	       X	             Y	             Z
Co 	     -0.033569	     -1.603845	     -1.119544
O  	      0.252975	     -2.467434	     -3.061998
O  	     -0.131989	     -3.802771	     -0.590289
O  	      0.298076	     -1.357423	      0.885903
O  	      2.019569	     -1.333196	     -1.449232
O  	     -1.983597	     -1.980420	     -0.708233
O  	     -0.392752	      0.385157	     -1.465289
C  	      1.218555	     -0.608188	      1.338591
C  	      2.340783	     -0.172799	      0.652751
C  	      2.673682	     -0.598802	     -0.670608
C  	      1.041679	     -0.164316	      2.803655
C  	      4.046380	     -0.015820	     -0.990414
C  	      5.017060	     -0.814857	     -0.055608
C  	      3.513285	      0.681745	      1.111214
H  	      3.299170	      1.371627	      1.935814
C  	      5.242685	      2.147785	     -0.160565
H  	      5.121164	      3.011959	      0.512381
H  	      6.095573	      1.561382	      0.204321
C  	      3.932151	      1.359100	     -0.233508
C  	      4.685881	     -0.300814	      1.373556
H  	      5.546224	      0.208945	      1.831313
H  	      4.376297	     -1.101633	      2.057742
C  	      4.409845	     -0.019100	     -2.459578
H  	      3.678585	      0.550844	     -3.052787
H  	      5.406216	      0.419900	     -2.621136
H  	      4.423517	     -1.047680	     -2.852258
C  	     -2.698140	     -1.077300	     -0.158292
C  	     -2.478086	      0.285129	     -0.213964
C  	     -1.383733	      0.908384	     -0.901629
C  	     -3.935424	     -1.592875	      0.594580
C  	     -1.574605	      2.415361	     -0.753293
C  	     -2.380940	      1.996020	      1.537153
C  	     -3.272333	      1.428304	      0.397463
H  	     -4.298749	      1.185850	      0.695614
C  	     -3.810973	      2.115741	     -2.037139
H  	     -4.902860	      2.049682	     -1.905932
H  	     -3.616050	      2.870978	     -2.814802
C  	     -3.141833	      2.513749	     -0.716105
C  	     -0.814996	      3.267632	     -1.747085
H  	     -1.105182	      3.030911	     -2.781998
H  	     -1.006126	      4.337159	     -1.569249
H  	      0.266851	      3.096061	     -1.661156
C  	     -1.205980	      2.645990	      0.753017
H  	     -1.125017	      3.726138	      0.945442
H  	     -0.229885	      2.205372	      0.996097
F  	      1.014428	      1.182746	      2.904788
F  	     -0.090048	     -0.628272	      3.329084
F  	      2.063245	     -0.596026	      3.568710
F  	     -3.942972	     -2.925574	      0.669422
F  	     -5.071603	     -1.208580	     -0.018004
F  	     -3.976689	     -1.113081	      1.851163
H  	      1.186067	     -2.198641	     -3.138652
H  	      0.287776	     -3.407096	     -2.818649
H  	      0.278000	     -3.766644	      0.287867
H  	     -1.080082	     -3.628039	     -0.411945
H  	      5.510777	      2.541227	     -1.153643
H  	      4.869166	     -1.899044	     -0.170706
H  	      6.057832	     -0.602423	     -0.341901
C  	      2.850076	      2.278982	     -0.812659
H  	      1.889360	      1.767138	     -0.958634
H  	      3.165725	      2.684800	     -1.786887
H  	      2.677169	      3.134532	     -0.140192
H  	     -2.922769	      2.734367	      2.145984
H  	     -2.047684	      1.192977	      2.207462
H  	     -3.464781	      1.143688	     -2.415123
C  	     -3.676006	      3.891624	     -0.312697
H  	     -4.765688	      3.838992	     -0.157369
H  	     -3.494247	      4.625699	     -1.113482
H  	     -3.229591	      4.288511	      0.607777

l-1_ID3
Gibbs energy -3365.23541500 h, no imaginary frequency
Symbol	       X	             Y	             Z
Co 	     -0.027719	     -0.851284	     -1.721458
O  	      0.348929	     -0.804779	     -3.828513
O  	     -0.314285	     -3.048965	     -2.192535
O  	     -0.172679	      1.093736	     -1.184195
O  	     -2.050357	     -1.144974	     -1.570941
O  	      0.246063	     -1.648707	      0.143792
O  	      2.072655	     -0.612021	     -1.850062
C  	     -1.149890	      1.517597	     -0.494958
C  	     -2.326906	      0.838043	     -0.204249
C  	     -2.693431	     -0.417374	     -0.767522
C  	     -0.962566	      2.925939	      0.104055
C  	     -4.083795	     -0.755693	     -0.243161
C  	     -3.969820	     -0.150002	      1.204511
C  	     -3.503674	      1.244182	      0.672886
H  	     -3.277157	      1.985690	      1.448006
C  	     -4.651378	      1.641496	     -0.293012
H  	     -4.307568	      2.391614	     -1.016851
H  	     -5.505697	      2.073448	      0.248348
C  	     -5.017023	      0.282825	     -0.953437
H  	     -4.858115	      0.273923	     -2.041969
H  	     -6.067931	      0.006963	     -0.780384
C  	     -2.921205	     -0.857965	      2.072377
H  	     -2.696733	     -0.260944	      2.970416
H  	     -1.974630	     -1.033830	      1.546852
H  	     -3.295634	     -1.836373	      2.412365
C  	     -5.289754	     -0.115390	      1.980030
H  	     -5.604114	     -1.135760	      2.250183
H  	     -6.117471	      0.352706	      1.432042
C  	     -4.484374	     -2.206536	     -0.402895
H  	     -4.496166	     -2.491221	     -1.466514
H  	     -5.489539	     -2.385589	      0.008295
H  	     -3.776749	     -2.873729	      0.113192
C  	      1.182139	     -1.264062	      0.923303
C  	      2.329454	     -0.588078	      0.557288
C  	      2.705553	     -0.342424	     -0.804142
C  	      0.956206	     -1.576423	      2.413938
C  	      4.091734	      0.291394	     -0.771012
C  	      3.954820	      1.149779	      0.541136
C  	      3.489598	     -0.073671	      1.395766
H  	      3.249169	      0.152315	      2.441566
C  	      4.648917	     -1.085916	      1.197416
H  	      5.495161	     -0.867376	      1.864894
H  	      4.315010	     -2.108824	      1.414686
C  	      5.025680	     -0.873071	     -0.295810
H  	      4.878031	     -1.772908	     -0.911724
H  	      6.076105	     -0.570456	     -0.419342
C  	      4.504832	      0.974521	     -2.056679
H  	      4.528558	      0.253378	     -2.888561
H  	      5.507649	      1.418479	     -1.962795
H  	      3.795916	      1.771146	     -2.328847
C  	      2.896863	      2.253854	      0.429506
H  	      2.668211	      2.670275	      1.423399
H  	      1.954483	      1.904517	     -0.009453
H  	      3.263800	      3.081313	     -0.197747
C  	      5.264803	      1.784285	      1.016954
H  	      6.100755	      1.077678	      1.098824
H  	      5.117230	      2.241654	      2.008646
F  	     -2.051780	      3.695340	     -0.086684
F  	      0.076992	      3.561601	     -0.435207
F  	     -0.754318	      2.848161	      1.432683
F  	     -0.050572	     -2.434165	      2.592103
F  	      2.052155	     -2.115575	      2.983171
F  	      0.663667	     -0.452317	      3.093883
H  	      0.361411	     -1.755681	     -4.024444
H  	      1.288169	     -0.559775	     -3.739794
H  	     -1.268375	     -2.911316	     -2.036398
H  	      0.016206	     -3.323946	     -1.320392
H  	      5.573938	      2.587313	      0.329714
H  	     -5.154133	      0.444420	      2.919517

l-1_ID4
Gibbs energy -3365.23481500 h, no imaginary frequency
Symbol	       X	             Y	             Z
Co 	      0.107921	     -1.735848	     -0.713067
O  	     -0.109309	     -2.999860	     -2.432892
O  	      0.161070	     -3.768928	      0.283729
O  	     -0.399088	     -1.142043	      1.176656
O  	     -1.905228	     -1.471119	     -1.288694
O  	      2.052853	     -2.045704	     -0.247078
O  	      0.464747	      0.146469	     -1.420643
C  	     -1.344638	     -0.322593	      1.415988
C  	     -2.409244	     -0.022663	      0.586113
C  	     -2.605802	     -0.615600	     -0.702100
C  	     -1.281210	      0.371269	      2.789896
C  	     -3.851161	      0.029063	     -1.295902
C  	     -4.709294	      0.236557	      0.003184
C  	     -3.552058	      0.967955	      0.757098
H  	     -3.777393	      1.257774	      1.790072
C  	     -3.227354	      2.148535	     -0.194205
H  	     -2.219955	      2.536513	      0.002007
H  	     -3.933598	      2.981466	     -0.064741
C  	     -3.358348	      1.490691	     -1.595897
H  	     -2.407305	      1.473300	     -2.147723
H  	     -4.096399	      2.003116	     -2.230633
C  	     -5.136021	     -1.075073	      0.672749
H  	     -5.618719	     -0.867849	      1.641079
H  	     -4.289884	     -1.749985	      0.863653
H  	     -5.866484	     -1.612947	      0.047880
C  	     -5.963626	      1.090223	     -0.207417
H  	     -6.722944	      0.525278	     -0.770727
H  	     -5.777361	      2.024588	     -0.751903
H  	     -6.406603	      1.354454	      0.766453
C  	     -4.453870	     -0.702571	     -2.475113
H  	     -3.719133	     -0.788449	     -3.290511
H  	     -5.333725	     -0.167755	     -2.864201
H  	     -4.763187	     -1.722669	     -2.199904
C  	      2.866693	     -1.083193	     -0.049595
C  	      2.659477	      0.244771	     -0.369194
C  	      1.495294	      0.748922	     -1.040378
C  	      4.191965	     -1.478448	      0.623583
C  	      1.724345	      2.241604	     -1.246829
C  	      2.503087	      2.576143	      0.079467
C  	      3.562930	      1.452395	     -0.160432
H  	      4.314852	      1.343664	      0.629843
C  	      4.133654	      1.814933	     -1.555524
H  	      4.894297	      2.606372	     -1.486178
H  	      4.607994	      0.941605	     -2.022105
C  	      2.868429	      2.299490	     -2.316100
H  	      2.621464	      1.674355	     -3.186982
H  	      2.973801	      3.331238	     -2.683009
C  	      0.482172	      3.033217	     -1.591846
H  	      0.034014	      2.661209	     -2.525724
H  	      0.719052	      4.099805	     -1.724752
H  	     -0.280414	      2.943093	     -0.804935
C  	      1.672793	      2.359762	      1.350704
H  	      2.288456	      2.552904	      2.243397
H  	      1.288414	      1.335590	      1.438906
H  	      0.815121	      3.048671	      1.386595
C  	      3.078493	      3.994594	      0.120413
H  	      2.272269	      4.730317	      0.268041
H  	      3.623761	      4.279848	     -0.788458
H  	      3.772223	      4.093288	      0.970874
F  	     -1.357910	      1.713184	      2.661846
F  	     -0.150675	      0.091967	      3.434958
F  	     -2.309986	     -0.010177	      3.572138
F  	      4.273688	     -2.795966	      0.812999
F  	      5.249432	     -1.103089	     -0.121104
F  	      4.318689	     -0.883762	      1.825240
H  	     -1.025524	     -2.727957	     -2.622738
H  	     -0.191019	     -3.865738	     -2.001146
H  	     -0.236851	     -3.481942	      1.121502
H  	      1.117183	     -3.605079	      0.413571

l-3_ID1
Gibbs energy -3477.81007200 h, no imaginary frequency
Symbol	       X	             Y	             Z
Co 	      0.000357	      1.506205	      0.000574
O  	      1.765253	      1.485971	     -1.051846
O  	      0.689970	     -0.022909	      1.251641
O  	     -1.760477	      1.529721	      1.053478
O  	     -0.746412	      0.046984	     -1.291866
C  	      2.647157	      0.581279	     -0.956553
C  	      2.682039	     -0.475786	     -0.060236
C  	      1.724142	     -0.673542	      0.985544
C  	      3.805735	      0.697173	     -1.966811
C  	      2.227537	     -1.851551	      1.820372
C  	      2.878195	     -2.730256	      0.692065
C  	      3.743285	     -1.547804	      0.150626
H  	      4.357881	     -1.786572	     -0.725654
C  	      4.545116	     -1.116189	      1.407533
H  	      4.913097	     -0.087160	      1.300663
H  	      5.417626	     -1.764888	      1.574374
C  	      3.492162	     -1.269047	      2.540396
H  	      3.254296	     -0.318182	      3.040276
H  	      3.823546	     -1.968529	      3.321923
C  	      1.866207	     -3.258351	     -0.331509
H  	      2.391624	     -3.736003	     -1.174030
H  	      1.227200	     -2.465269	     -0.742310
H  	      1.211669	     -4.017220	      0.124020
C  	      3.689256	     -3.920971	      1.212478
H  	      3.017515	     -4.691812	      1.621770
H  	      4.408659	     -3.658747	      1.998532
H  	      4.252476	     -4.380531	      0.384052
C  	      1.198856	     -2.474420	      2.739298
H  	      0.795793	     -1.720480	      3.432921
H  	      1.643679	     -3.285913	      3.335162
H  	      0.351379	     -2.887289	      2.172568
C  	     -2.660765	      0.642964	      0.954648
C  	     -2.725867	     -0.397936	      0.041339
C  	     -1.786410	     -0.592672	     -1.021228
C  	     -3.803790	      0.762277	      1.981913
C  	     -2.317284	     -1.750542	     -1.866526
C  	     -2.964678	     -2.637123	     -0.742432
C  	     -3.805556	     -1.450997	     -0.171456
H  	     -4.411570	     -1.694374	      0.709509
C  	     -4.618019	     -0.989117	     -1.410695
H  	     -5.502294	     -1.622433	     -1.574553
H  	     -4.969139	      0.043507	     -1.283739
C  	     -3.583402	     -1.140692	     -2.560600
H  	     -3.339422	     -0.186337	     -3.050733
H  	     -3.935579	     -1.824590	     -3.346813
C  	     -1.308213	     -2.373200	     -2.807130
H  	     -0.907218	     -1.615463	     -3.497808
H  	     -1.769486	     -3.173380	     -3.405768
H  	     -0.456973	     -2.800982	     -2.256986
C  	     -1.945739	     -3.194063	      0.258559
H  	     -2.465533	     -3.676124	      1.102067
H  	     -1.289291	     -2.416139	      0.670711
H  	     -1.309099	     -3.955660	     -0.217271
C  	     -3.799427	     -3.808583	     -1.269094
H  	     -3.144261	     -4.582539	     -1.698863
H  	     -4.525582	     -3.524645	     -2.041249
H  	     -4.357762	     -4.272477	     -0.439802
F  	      5.002605	      0.765604	     -1.346398
F  	      3.692658	      1.783919	     -2.734077
F  	      3.850212	     -0.372941	     -2.788025
F  	     -3.662637	      1.835015	      2.764056
F  	     -5.007571	      0.859408	      1.379112
F  	     -3.854504	     -0.319682	      2.787205
N  	     -0.673073	      3.095945	     -1.270605
N  	      0.793517	      2.979137	      1.348084
C  	     -1.068154	      3.927747	     -1.966961
C  	      1.328273	      3.696141	      2.078239
C  	     -1.565204	      4.977605	     -2.839611
H  	     -2.598936	      4.752927	     -3.140658
H  	     -0.934814	      5.042799	     -3.738366
H  	     -1.543830	      5.942279	     -2.311930
C  	      1.999747	      4.597223	      2.998965
H  	      1.357001	      4.785137	      3.871337
H  	      2.216191	      5.551238	      2.496641
H  	      2.943679	      4.145321	      3.337255

l-3_ID2
Gibbs energy -3477.80950100 h, no imaginary frequency
Symbol	       X	             Y	             Z
Co 	      0.042768	      1.556472	      0.153151
O  	     -0.392010	      0.272931	     -1.420285
O  	     -1.872181	      1.320964	      0.911305
O  	      1.976619	      1.714604	     -0.519274
O  	      0.546813	     -0.131285	      1.279954
C  	     -1.350684	     -0.554587	     -1.390866
C  	     -2.399757	     -0.596297	     -0.485977
C  	     -2.574543	      0.338155	      0.583564
C  	     -1.345472	     -1.603779	     -2.520571
C  	     -3.825449	     -0.097518	      1.342893
C  	     -4.697946	     -0.649124	      0.159691
C  	     -3.556308	     -1.579523	     -0.361700
H  	     -3.799108	     -2.147885	     -1.267332
C  	     -3.238681	     -2.449200	      0.883050
H  	     -2.235301	     -2.887788	      0.804097
H  	     -3.954645	     -3.276427	      0.995138
C  	     -3.359249	     -1.422321	      2.043320
H  	     -2.410698	     -1.267586	      2.578492
H  	     -4.107056	     -1.725979	      2.790586
C  	     -5.112702	      0.427240	     -0.850729
H  	     -5.603753	     -0.038529	     -1.720263
H  	     -4.259893	      1.011799	     -1.223715
H  	     -5.833505	      1.127163	     -0.399450
C  	     -5.962475	     -1.390035	      0.605813
H  	     -6.707372	     -0.677343	      0.993340
H  	     -5.784558	     -2.138524	      1.388279
H  	     -6.417175	     -1.907229	     -0.254677
C  	     -4.410147	      0.947678	      2.267764
H  	     -3.665963	      1.256544	      3.017963
H  	     -5.290551	      0.555768	      2.799546
H  	     -4.713736	      1.849192	      1.713548
C  	      2.808707	      0.757279	     -0.490216
C  	      2.675040	     -0.458307	      0.160859
C  	      1.569239	     -0.795750	      1.005784
C  	      4.103555	      0.998753	     -1.289540
C  	      1.874483	     -2.169660	      1.597855
C  	      2.621268	     -2.829966	      0.381890
C  	      3.629314	     -1.642798	      0.247742
H  	      4.346545	     -1.734410	     -0.576884
C  	      4.268829	     -1.585438	      1.660076
H  	      5.064555	     -2.336294	      1.773961
H  	      4.714208	     -0.599300	      1.847441
C  	      3.057985	     -1.889626	      2.585851
H  	      2.812862	     -1.058278	      3.263594
H  	      3.229606	     -2.775964	      3.214223
C  	      0.684059	     -2.879683	      2.205030
H  	      0.252436	     -2.281154	      3.022031
H  	      0.975298	     -3.858674	      2.614877
H  	     -0.109849	     -3.038859	      1.460215
C  	      1.736408	     -3.010237	     -0.856719
H  	      2.330119	     -3.419428	     -1.689977
H  	      1.295228	     -2.066058	     -1.198868
H  	      0.916125	     -3.716497	     -0.656963
C  	      3.267371	     -4.180263	      0.706168
H  	      2.494058	     -4.957639	      0.810277
H  	      3.860565	     -4.179183	      1.629504
H  	      3.932101	     -4.484799	     -0.118439
F  	     -1.402039	     -2.862402	     -2.034431
F  	     -0.246527	     -1.519687	     -3.276374
F  	     -2.408610	     -1.451866	     -3.337505
F  	      4.158855	      2.235497	     -1.789986
F  	      5.203064	      0.823618	     -0.527172
F  	      4.204157	      0.139725	     -2.326296
N  	      0.458884	      2.865827	      1.799113
N  	     -0.512025	      3.263542	     -1.005347
C  	      0.751385	      3.517305	      2.706312
C  	     -0.870420	      4.154529	     -1.646303
C  	     -1.318003	      5.276076	     -2.453591
H  	     -1.759446	      4.907017	     -3.390843
H  	     -0.463985	      5.927753	     -2.689204
H  	     -2.072719	      5.855116	     -1.901791
C  	      1.121129	      4.336079	      3.847848
H  	      0.322325	      5.061732	      4.059513
H  	      2.052867	      4.878377	      3.630403
H  	      1.272003	      3.697462	      4.730417

l-3_ID3
Gibbs energy -3477.81232600 h, no imaginary frequency
Symbol	       X	             Y	             Z
Co 	      0.000091	      1.659006	      0.001368
O  	      0.202596	      0.188804	      1.450156
O  	      2.039145	      1.545331	     -0.387409
O  	     -0.199981	      0.187091	     -1.446610
O  	     -2.038758	      1.542196	      0.390564
C  	      1.099682	     -0.702445	      1.376396
C  	      2.259114	     -0.657147	      0.619376
C  	      2.632030	      0.458479	     -0.196747
C  	      0.845021	     -1.960491	      2.227655
C  	      3.934921	      0.070584	     -0.891212
C  	      4.608620	     -0.805164	      0.223783
C  	      3.343114	     -1.703811	      0.403457
H  	      3.425739	     -2.472381	      1.181071
C  	      3.117372	     -2.253395	     -1.029986
H  	      2.083585	     -2.596558	     -1.159123
H  	      3.780788	     -3.103887	     -1.245709
C  	      3.455429	     -1.020804	     -1.913695
H  	      2.587553	     -0.665001	     -2.485830
H  	      4.262144	     -1.230882	     -2.631584
C  	      4.977334	     -0.015521	      1.485283
H  	      5.321764	     -0.701998	      2.275478
H  	      4.131728	      0.559062	      1.888831
H  	      5.798861	      0.688251	      1.277721
C  	      5.858058	     -1.559372	     -0.242354
H  	      6.697130	     -0.860045	     -0.383919
H  	      5.720417	     -2.103782	     -1.185083
H  	      6.163431	     -2.289919	      0.524367
C  	      4.708643	      1.221910	     -1.495534
H  	      4.087264	      1.758481	     -2.229017
H  	      5.613185	      0.862878	     -2.009881
H  	      5.013739	      1.948666	     -0.726767
C  	     -1.096961	     -0.704300	     -1.373990
C  	     -2.256521	     -0.660123	     -0.617058
C  	     -2.630730	      0.455006	      0.199142
C  	     -0.842451	     -1.961139	     -2.227131
C  	     -3.934224	      0.066301	      0.892095
C  	     -4.606409	     -0.809079	     -0.224130
C  	     -3.340423	     -1.707238	     -0.402811
H  	     -3.421963	     -2.475484	     -1.180848
C  	     -3.116071	     -2.257447	      1.030618
H  	     -3.779341	     -3.108349	      1.245174
H  	     -2.082254	     -2.600184	      1.160754
C  	     -3.455696	     -1.025458	      1.914569
H  	     -2.588749	     -0.669685	      2.488128
H  	     -4.263269	     -1.236220	      2.631288
C  	     -4.708989	      1.217111	      1.496057
H  	     -4.088703	      1.753428	      2.230651
H  	     -5.614110	      0.857578	      2.009035
H  	     -5.013257	      1.944227	      0.727304
C  	     -4.973896	     -0.018881	     -1.485643
H  	     -5.317082	     -0.705055	     -2.276641
H  	     -4.128033	      0.556267	     -1.887841
H  	     -5.795959	      0.684443	     -1.278686
C  	     -5.856160	     -1.563915	      0.240148
H  	     -6.695565	     -0.864904	      0.381303
H  	     -5.719400	     -2.108971	      1.182633
H  	     -6.160525	     -2.293995	     -0.527418
F  	      0.716320	     -3.055164	      1.445956
F  	     -0.273124	     -1.859211	      2.953694
F  	      1.856126	     -2.205698	      3.085740
F  	      0.275672	     -1.858949	     -2.953074
F  	     -0.713950	     -3.057055	     -1.447158
F  	     -1.853625	     -2.204801	     -3.085592
N  	      0.173219	      3.192629	      1.474250
N  	     -0.177834	      3.188487	     -1.476123
C  	      0.296795	      3.985827	      2.303996
C  	     -0.306382	      3.977067	     -2.309514
C  	      0.452189	      4.981839	      3.349991
H  	      1.407225	      4.825175	      3.872494
H  	     -0.372517	      4.894668	      4.072354
H  	      0.442764	      5.989611	      2.909938
C  	     -0.468238	      4.967988	     -3.359328
H  	      0.298830	      5.749233	     -3.254679
H  	     -1.464321	      5.428397	     -3.286366
H  	     -0.363068	      4.488182	     -4.343281

l-3_ID4
Gibbs energy -3477.80840500 h, no imaginary frequency
Symbol	       X	             Y	             Z
O  	      0.304161	      0.426014	     -1.378747
O  	      1.989867	      1.306472	      0.864009
C  	      1.230778	     -0.429682	     -1.474151
C  	      2.332380	     -0.568658	     -0.643343
C  	      2.636375	      0.324850	      0.431400
C  	      1.098402	     -1.404502	     -2.661662
C  	      3.992085	     -0.108025	      0.989368
C  	      3.878403	     -1.664387	      0.798570
C  	      3.505233	     -1.536589	     -0.713200
H  	      3.306144	     -2.486090	     -1.224195
C  	      4.700453	     -0.729403	     -1.287476
H  	      5.565109	     -1.377219	     -1.493862
H  	      4.421915	     -0.239129	     -2.229847
C  	      5.000372	      0.280370	     -0.144447
H  	      4.864043	      1.329199	     -0.447639
H  	      6.029951	      0.185842	      0.231201
C  	      4.319016	      0.441083	      2.361282
H  	      4.337140	      1.541713	      2.342923
H  	      5.304505	      0.088710	      2.702260
H  	      3.565719	      0.135956	      3.103801
C  	      2.768021	     -2.298412	      1.645875
H  	      2.608419	     -3.346098	      1.343704
H  	      1.809343	     -1.771207	      1.548939
H  	      3.046612	     -2.301259	      2.711624
C  	      5.178108	     -2.429244	      1.065188
H  	      5.410987	     -2.426725	      2.141639
H  	      6.049486	     -2.021135	      0.537487
H  	      5.062584	     -3.480647	      0.755386
F  	     -0.013563	     -1.181101	     -3.368703
F  	      2.144125	     -1.300134	     -3.506385
F  	      1.055576	     -2.691926	     -2.250911
O  	     -1.926509	      1.808530	     -0.331722
O  	     -0.489301	     -0.128982	      1.359770
C  	     -2.661822	      0.789101	     -0.505296
C  	     -2.508227	     -0.466210	      0.059366
C  	     -1.471122	     -0.808390	      0.984993
C  	     -3.882926	      1.012808	     -1.416755
C  	     -1.732010	     -2.249540	      1.424556
C  	     -3.300587	     -2.298588	      1.357107
C  	     -3.342899	     -1.727970	     -0.095522
H  	     -4.347990	     -1.589793	     -0.510575
C  	     -2.451166	     -2.730613	     -0.880713
H  	     -3.013092	     -3.628040	     -1.178449
H  	     -2.055376	     -2.265790	     -1.793449
C  	     -1.334842	     -3.066108	      0.148704
H  	     -0.329822	     -2.793143	     -0.199403
H  	     -1.308064	     -4.137796	      0.395372
C  	     -1.045139	     -2.674393	      2.704679
H  	      0.047126	     -2.601349	      2.603361
H  	     -1.293544	     -3.717783	      2.952755
H  	     -1.344532	     -2.037870	      3.551557
C  	     -3.985275	     -1.393818	      2.388623
H  	     -5.068592	     -1.345781	      2.192621
H  	     -3.598953	     -0.365052	      2.374865
H  	     -3.850218	     -1.793935	      3.405891
C  	     -3.894545	     -3.703988	      1.495994
H  	     -3.772601	     -4.073876	      2.526277
H  	     -3.443720	     -4.444275	      0.823107
H  	     -4.975347	     -3.674574	      1.282526
F  	     -3.884925	      2.232532	     -1.960403
F  	     -3.913280	      0.121107	     -2.429271
F  	     -5.040879	      0.873453	     -0.735879
Co 	      0.016951	      1.612762	      0.298165
N  	      0.537290	      3.388146	     -0.779518
N  	     -0.263443	      2.812728	      2.039042
C  	      0.877420	      4.307764	     -1.389094
C  	     -0.450665	      3.415435	      3.005763
C  	      1.304806	      5.464872	     -2.156450
H  	      2.339085	      5.320167	     -2.501326
H  	      0.648535	      5.596258	     -3.029113
H  	      1.256883	      6.366667	     -1.528798
C  	     -0.686405	      4.172771	      4.222612
H  	      0.157510	      4.036653	      4.914521
H  	     -0.789103	      5.241211	      3.983334
H  	     -1.609324	      3.820651	      4.706070

Zntfc2acn2_cis
SCF energy -3873.85693381 h
Symbol	       X	             Y	             Z
Zn 	      0.000356	      1.492595	      0.000569
O  	      1.765252	      1.472358	     -1.051851
O  	      0.689967	     -0.036520	      1.251636
O  	     -1.760478	      1.516113	      1.053473
O  	     -0.746415	      0.033375	     -1.291871
C  	      2.647155	      0.567665	     -0.956558
C  	      2.682035	     -0.489400	     -0.060241
C  	      1.724138	     -0.687155	      0.985539
C  	      3.805733	      0.683557	     -1.966816
C  	      2.227531	     -1.865165	      1.820367
C  	      2.878188	     -2.743871	      0.692060
C  	      3.743279	     -1.561420	      0.150621
H  	      4.357875	     -1.800189	     -0.725659
C  	      4.545111	     -1.129806	      1.407528
H  	      4.913094	     -0.100778	      1.300658
H  	      5.417620	     -1.778507	      1.574369
C  	      3.492157	     -1.282663	      2.540391
H  	      3.254292	     -0.331797	      3.040271
H  	      3.823540	     -1.982145	      3.321918
C  	      1.866199	     -3.271964	     -0.331514
H  	      2.391615	     -3.749617	     -1.174035
H  	      1.227193	     -2.478881	     -0.742315
H  	      1.211660	     -4.030832	      0.124015
C  	      3.689247	     -3.934587	      1.212473
H  	      3.017505	     -4.705427	      1.621765
H  	      4.408650	     -3.672364	      1.998527
H  	      4.252466	     -4.394148	      0.384047
C  	      1.198849	     -2.488032	      2.739293
H  	      0.795787	     -1.734092	      3.432916
H  	      1.643671	     -3.299526	      3.335157
H  	      0.351371	     -2.900900	      2.172563
C  	     -2.660767	      0.629358	      0.954643
C  	     -2.725871	     -0.411542	      0.041334
C  	     -1.786414	     -0.606280	     -1.021233
C  	     -3.803792	      0.748672	      1.981908
C  	     -2.317290	     -1.764149	     -1.866531
C  	     -2.964685	     -2.650729	     -0.742437
C  	     -3.805561	     -1.464602	     -0.171461
H  	     -4.411576	     -1.707978	      0.709504
C  	     -4.618024	     -1.002720	     -1.410700
H  	     -5.502300	     -1.636035	     -1.574558
H  	     -4.969142	      0.029904	     -1.283744
C  	     -3.583407	     -1.154297	     -2.560605
H  	     -3.339426	     -0.199942	     -3.050738
H  	     -3.935585	     -1.838194	     -3.346818
C  	     -1.308220	     -2.386808	     -2.807135
H  	     -0.907224	     -1.629072	     -3.497813
H  	     -1.769494	     -3.186988	     -3.405773
H  	     -0.456981	     -2.814592	     -2.256991
C  	     -1.945747	     -3.207670	      0.258554
H  	     -2.465542	     -3.689731	      1.102062
H  	     -1.289298	     -2.429747	      0.670706
H  	     -1.309108	     -3.969268	     -0.217276
C  	     -3.799436	     -3.822188	     -1.269099
H  	     -3.144271	     -4.596145	     -1.698868
H  	     -4.525591	     -3.538248	     -2.041254
H  	     -4.357772	     -4.286081	     -0.439807
F  	      5.002603	      0.751986	     -1.346403
F  	      3.692657	      1.770303	     -2.734082
F  	      3.850208	     -0.386557	     -2.788030
F  	     -3.662637	      1.821410	      2.764051
F  	     -5.007573	      0.845805	      1.379107
F  	     -3.854508	     -0.333286	      2.787200
N  	     -0.673072	      3.082336	     -1.270610
N  	      0.793518	      2.965525	      1.348079
C  	     -1.068151	      3.914138	     -1.966966
C  	      1.328275	      3.682529	      2.078234
C  	     -1.565200	      4.963997	     -2.839616
H  	     -2.598932	      4.739321	     -3.140663
H  	     -0.934810	      5.029190	     -3.738371
H  	     -1.543824	      5.928671	     -2.311935
C  	      1.999751	      4.583610	      2.998960
H  	      1.357005	      4.771525	      3.871332
H  	      2.216196	      5.537624	      2.496636
H  	      2.943682	      4.131706	      3.337250

Zntfc2acn2_trans
SCF energy -3873.85330425 h
Symbol	       X	             Y	             Z
Zn 	      0.008352	      0.016316	      0.056815
F  	     -4.113193	      2.741719	     -1.737107
F  	     -2.393862	      3.778930	     -0.934001
F  	     -3.954720	      3.089841	      0.399047
F  	      2.441578	     -3.711548	      1.199714
F  	      3.856396	     -3.129510	     -0.333209
F  	      4.246303	     -2.653535	      1.747068
O  	     -1.251855	      1.482489	     -0.639266
O  	     -1.545265	     -1.359067	      0.011521
O  	      1.274414	     -1.454428	      0.737142
O  	      1.559017	      1.393185	      0.131976
N  	      0.697717	     -0.622242	     -1.900990
N  	     -0.718813	      0.611132	      2.011155
C  	      3.269278	     -2.736391	      0.823063
C  	      3.942673	      1.989425	      0.053583
C  	     -6.415136	     -1.831252	     -0.553008
H  	     -7.136107	     -1.209085	     -1.107639
H  	     -6.748590	     -1.875813	      0.491430
H  	     -6.475906	     -2.851407	     -0.963438
C  	     -5.000573	     -1.269775	     -0.728882
C  	     -3.925685	     -1.926047	      0.210148
C  	     -2.740994	     -0.993438	     -0.032137
C  	     -3.284315	      0.313315	     -0.248495
C  	     -2.513342	      1.431445	     -0.524012
C  	     -3.242635	      2.775305	     -0.708569
C  	     -4.792505	      0.153623	     -0.115961
H  	     -5.396547	      0.947656	     -0.570188
C  	     -5.071614	     -0.089887	      1.390806
H  	     -6.149830	     -0.097828	      1.606440
H  	     -4.621433	      0.701127	      2.004170
C  	     -4.428591	     -1.484619	      1.628688
H  	     -5.157298	     -2.218001	      2.003797
H  	     -3.597017	     -1.462129	      2.348740
C  	     -4.643172	     -1.350521	     -2.218049
H  	     -3.631247	     -0.980320	     -2.434659
H  	     -5.352224	     -0.751022	     -2.811246
H  	     -4.708024	     -2.389964	     -2.576266
C  	     -3.620119	     -3.401101	      0.065902
H  	     -2.828018	     -3.701017	      0.769101
H  	     -3.268526	     -3.640078	     -0.949570
H  	     -4.512485	     -4.010381	      0.275422
C  	      2.536092	     -1.396310	      0.628264
C  	      2.756755	      1.031745	      0.161504
C  	      4.956704	      1.079676	     -0.730735
C  	      4.813904	     -0.102170	      0.281423
H  	      5.392850	     -0.999830	      0.032080
C  	      3.302400	     -0.281173	      0.325316
C  	      1.365909	     -1.245876	     -2.607866
C  	      2.241170	     -2.049372	     -3.442391
H  	      1.655292	     -2.798569	     -3.994168
H  	      2.968198	     -2.560716	     -2.793766
H  	      2.778079	     -1.408754	     -4.156385
C  	      3.608570	      3.359789	     -0.494961
H  	      4.507174	      3.993306	     -0.546105
H  	      2.871203	      3.864122	      0.148401
H  	      3.174095	      3.292131	     -1.504182
C  	      4.556576	      1.976334	      1.495471
H  	      5.301608	      2.780712	      1.582901
H  	      3.782848	      2.171240	      2.252880
C  	      5.199347	      0.566753	      1.628764
H  	      6.290317	      0.628136	      1.753557
H  	      4.801131	     -0.006523	      2.476313
C  	      4.489194	      0.733765	     -2.149362
H  	      5.124056	     -0.056538	     -2.580705
H  	      4.560960	      1.613955	     -2.806999
H  	      3.449596	      0.383511	     -2.175144
C  	      6.373340	      1.654301	     -0.829930
H  	      7.054667	      0.895697	     -1.248255
H  	      6.789215	      1.980315	      0.131581
H  	      6.388626	      2.521142	     -1.508910
C  	     -2.382541	      1.829987	      3.605741
H  	     -3.052559	      1.104288	      4.088533
H  	     -2.978560	      2.529020	      3.000266
H  	     -1.838858	      2.391378	      4.379236
C  	     -1.446116	      1.140476	      2.736120

Zn-monomer_DFTgeo
Gibbs energy -3874.32141500 h, no imaginary frequency
Symbol	       X	             Y	             Z
Zn 	     -0.010034	      0.021124	     -0.077172
F  	      4.147997	      2.697302	      1.742165
F  	      2.439797	      3.766761	      0.959791
F  	      3.989628	      3.080639	     -0.389822
F  	     -2.492243	     -3.682011	     -1.267925
F  	     -3.911768	     -3.130131	      0.273722
F  	     -4.275742	     -2.578440	     -1.794023
O  	      1.270962	      1.500925	      0.545054
O  	      1.538518	     -1.339053	     -0.121160
O  	     -1.297851	     -1.465762	     -0.669231
O  	     -1.553666	      1.385654	     -0.065734
N  	     -0.595061	     -0.656544	      2.022547
N  	      0.644285	      0.656832	     -2.167328
C  	     -3.304492	     -2.709839	     -0.872718
C  	     -3.924635	      2.009094	      0.015821
C  	      6.367057	     -1.933463	      0.606859
H  	      7.079895	     -1.351595	      1.213609
H  	      6.743013	     -1.946669	     -0.424202
H  	      6.389147	     -2.970023	      0.978634
C  	      4.958795	     -1.346104	      0.747211
C  	      3.909211	     -1.942311	     -0.257573
C  	      2.733651	     -0.992755	     -0.020261
C  	      3.295902	      0.292333	      0.259230
C  	      2.530452	      1.424528	      0.506551
C  	      3.271533	      2.759837	      0.724212
C  	      4.804988	      0.103975	      0.181815
H  	      5.404828	      0.868720	      0.688839
C  	      5.143528	     -0.092198	     -1.319930
H  	      6.229674	     -0.119593	     -1.492522
H  	      4.740898	      0.734846	     -1.918810
C  	      4.476269	     -1.460230	     -1.636985
H  	      5.200568	     -2.195686	     -2.017883
H  	      3.670278	     -1.389878	     -2.383225
C  	      4.539233	     -1.473428	      2.216730
H  	      3.527089	     -1.089587	      2.404658
H  	      5.234049	     -0.910756	      2.860743
H  	      4.569194	     -2.526465	      2.538618
C  	      3.558856	     -3.412283	     -0.182634
H  	      2.763260	     -3.651614	     -0.904264
H  	      3.181316	     -3.681280	      0.815626
H  	      4.434233	     -4.042024	     -0.405424
C  	     -2.557393	     -1.384874	     -0.619823
C  	     -2.751352	      1.035542	     -0.113856
C  	     -4.961812	      1.091515	      0.757767
C  	     -4.825642	     -0.059843	     -0.290838
H  	     -5.421945	     -0.956323	     -0.079711
C  	     -3.316862	     -0.262089	     -0.319110
C  	     -1.343475	     -1.357414	      2.555290
C  	     -2.311147	     -2.252769	      3.168210
H  	     -1.795082	     -3.059450	      3.709272
H  	     -2.938123	     -2.688607	      2.375732
H  	     -2.952234	     -1.697115	      3.867806
C  	     -3.565401	      3.352895	      0.611539
H  	     -4.443484	      4.015751	      0.657008
H  	     -2.789734	      3.844105	      0.004932
H  	     -3.156110	      3.243375	      1.627317
C  	     -4.521849	      2.052989	     -1.432012
H  	     -5.252827	      2.872331	     -1.503998
H  	     -3.734752	      2.259575	     -2.172136
C  	     -5.184850	      0.658646	     -1.619533
H  	     -6.273291	      0.740607	     -1.757753
H  	     -4.784697	      0.105285	     -2.478948
C  	     -4.515585	      0.692625	      2.169947
H  	     -5.168233	     -0.101878	      2.567624
H  	     -4.582641	      1.551414	      2.856030
H  	     -3.480103	      0.329022	      2.193240
C  	     -6.370159	      1.685578	      0.861831
H  	     -7.070012	      0.928236	      1.251584
H  	     -6.771058	      2.044837	     -0.094177
H  	     -6.379281	      2.534089	      1.564043
C  	      2.512905	      2.059398	     -3.328630
H  	      3.231431	      1.400706	     -3.837717
H  	      3.041987	      2.630582	     -2.551098
H  	      2.077236	      2.755108	     -4.060824
C  	      1.467146	      1.267816	     -2.700877

Zn-monomer_molA
SCF energy -3874.64133217 h, 1 imaginary frequency
Symbol	       X	             Y	             Z
Zn 	      0.017297	     -0.001233	      0.311869
F  	     -2.272445	     -3.924421	      0.023539
F  	     -3.753051	     -3.037947	     -1.282793
F  	     -4.100482	     -3.121751	      0.858005
F  	      2.284181	      3.957181	     -0.009475
F  	      4.137737	      3.164733	      0.774689
F  	      3.739185	      3.087257	     -1.355826
O  	     -1.243413	     -1.569491	      0.336063
O  	     -1.590688	      1.289259	      0.331129
O  	      1.270489	      1.596086	      0.310992
O  	      1.631380	     -1.273089	      0.187749
N  	      0.164894	      0.059958	      2.484153
N  	     -0.142475	     -0.061566	     -1.874878
C  	      3.170115	      2.960710	     -0.142577
C  	      3.974802	     -1.824202	     -0.314132
C  	     -6.529253	      1.557035	      0.206965
H  	     -7.293147	      0.809696	      0.479398
H  	     -6.701908	      1.840999	     -0.839273
H  	     -6.714044	      2.451832	      0.822227
C  	     -5.125586	      0.995189	      0.480841
C  	     -3.949747	      1.862847	     -0.112066
C  	     -2.767739	      0.913502	      0.104403
C  	     -3.274372	     -0.412311	     -0.055569
C  	     -2.479790	     -1.545778	      0.079134
C  	     -3.148816	     -2.929039	     -0.078837
C  	     -4.752273	     -0.254919	     -0.384570
H  	     -5.373718	     -1.143884	     -0.216693
C  	     -4.814375	      0.312625	     -1.831131
H  	     -5.846526	      0.348545	     -2.212068
H  	     -4.236442	     -0.320316	     -2.520082
C  	     -4.215467	      1.738335	     -1.656499
H  	     -4.908849	      2.529694	     -1.975781
H  	     -3.284124	      1.893962	     -2.222897
C  	     -5.014862	      0.748881	      1.994267
H  	     -4.037074	      0.344583	      2.292490
H  	     -5.783218	      0.028673	      2.320528
H  	     -5.182886	      1.682863	      2.554655
C  	     -3.754047	      3.279766	      0.388825
H  	     -2.911624	      3.770361	     -0.121825
H  	     -3.547207	      3.302469	      1.470264
H  	     -4.650040	      3.888075	      0.202626
C  	      2.505405	      1.575484	      0.027599
C  	      2.797537	     -0.883733	     -0.031405
C  	      5.157599	     -0.992192	      0.313130
C  	      4.779750	      0.304080	     -0.483410
H  	      5.408469	      1.179609	     -0.275532
C  	      3.305643	      0.451587	     -0.129363
C  	      0.270668	     -0.080246	      3.624981
C  	      0.405386	     -0.264424	      5.063101
H  	     -0.477403	      0.140236	      5.580444
H  	      1.304135	      0.254428	      5.428794
H  	      0.492934	     -1.336681	      5.294417
C  	      3.755909	     -3.262281	      0.115917
H  	      4.613657	     -3.897546	     -0.156677
H  	      2.855133	     -3.672983	     -0.365761
H  	      3.600721	     -3.339757	      1.203426
C  	      4.225790	     -1.619634	     -1.850740
H  	      4.922671	     -2.388780	     -2.218346
H  	      3.290778	     -1.742211	     -2.418745
C  	      4.824600	     -0.186974	     -1.958002
H  	      5.852484	     -0.203655	     -2.351723
H  	      4.238078	      0.477451	     -2.607503
C  	      5.060396	     -0.826188	      1.838312
H  	      5.832646	     -0.125135	      2.195566
H  	      5.231253	     -1.788675	      2.347546
H  	      4.085344	     -0.437855	      2.164967
C  	      6.557890	     -1.541671	     -0.001395
H  	      7.327022	     -0.819121	      0.318642
H  	      6.727886	     -1.752798	     -1.065328
H  	      6.740203	     -2.476975	      0.552264
C  	     -0.704025	     -0.098870	     -4.423576
H  	     -0.238505	      0.761943	     -4.926487
H  	     -1.794651	     -0.044835	     -4.559798
H  	     -0.332257	     -1.027692	     -4.881306
C  	     -0.385121	     -0.078561	     -3.003229

Zn-monomer_molB
SCF energy -3874.64061574 h, 2 imaginary frequencies
Symbol	       X	             Y	             Z
Zn 	      0.016666	     -0.052173	     -0.191919
F  	      2.368448	     -3.913842	      0.487563
F  	      3.711933	     -2.798699	      1.768794
F  	      4.246266	     -3.183380	     -0.297608
F  	     -3.958856	      2.994830	     -1.415147
F  	     -3.828976	      3.308991	      0.727877
F  	     -2.209949	      3.940504	     -0.561012
O  	      1.600106	      1.198732	     -0.585391
O  	      1.269863	     -1.638175	     -0.070128
O  	     -1.195944	      1.568579	     -0.353497
O  	     -1.605014	     -1.247036	      0.271036
N  	     -0.327570	     -0.479047	     -2.281003
N  	      0.379368	      0.383161	      1.919717
C  	     -3.998079	     -1.696793	      0.614377
C  	     -3.107814	      2.977750	     -0.367164
C  	      6.528094	      1.547689	     -0.616204
H  	      7.298994	      0.785217	     -0.818141
H  	      6.727462	      1.959790	      0.381873
H  	      6.678416	      2.362040	     -1.343487
C  	      5.127715	      0.934054	     -0.775150
C  	      3.949822	      1.859553	     -0.291489
C  	      2.777638	      0.868783	     -0.340228
C  	      3.309602	     -0.416237	      0.004896
C  	      2.518480	     -1.556260	      0.115533
C  	      3.205750	     -2.881037	      0.519424
C  	      4.791901	     -0.183765	      0.272106
H  	      5.430858	     -1.074812	      0.225469
C  	      4.869782	      0.592570	      1.618270
H  	      5.907320	      0.697207	      1.972051
H  	      4.310239	      0.063233	      2.402408
C  	      4.247229	      1.970055	      1.245743
H  	      3.326108	      2.196936	      1.804625
H  	      4.943468	      2.802777	      1.431687
C  	      4.990313	      0.475121	     -2.235068
H  	      4.010163	      0.029534	     -2.453737
H  	      5.760253	     -0.277772	     -2.472076
H  	      5.138559	      1.321728	     -2.924997
C  	      3.691130	      3.179384	     -0.992874
H  	      2.790969	      3.660198	     -0.579791
H  	      3.509377	      3.035202	     -2.069282
H  	      4.540061	      3.871784	     -0.874404
C  	     -2.448663	      1.589719	     -0.201729
C  	     -2.781234	     -0.820505	      0.286933
C  	     -5.096346	     -1.001569	     -0.278365
C  	     -4.781212	      0.420939	      0.297494
H  	     -5.369571	      1.240305	     -0.135589
C  	     -3.275330	      0.504348	      0.078648
C  	     -0.577885	     -1.090674	     -3.228685
C  	     -0.892681	     -1.894408	     -4.396404
H  	     -0.085256	     -2.619754	     -4.587870
H  	     -1.035902	     -1.248824	     -5.275312
H  	     -1.804366	     -2.478370	     -4.211368
C  	     -3.760539	     -3.189717	      0.484597
H  	     -4.645964	     -3.764109	      0.801633
H  	     -2.908201	     -3.495829	      1.111032
H  	     -3.518357	     -3.474366	     -0.550343
C  	     -4.406413	     -1.210019	      2.050342
H  	     -5.150890	     -1.898664	      2.479769
H  	     -3.538278	     -1.221096	      2.727626
C  	     -4.989532	      0.215381	      1.824564
H  	     -4.464139	      0.992426	      2.397085
H  	     -6.053608	      0.268110	      2.103319
C  	     -6.529274	     -1.491171	     -0.014218
H  	     -7.252050	     -0.839343	     -0.533039
H  	     -6.808473	     -1.511936	      1.047639
H  	     -6.667480	     -2.510014	     -0.411032
C  	     -4.845073	     -1.114409	     -1.790726
H  	     -5.574847	     -0.497082	     -2.340745
H  	     -4.964118	     -2.152013	     -2.138956
H  	     -3.841427	     -0.774118	     -2.081327
C  	      0.556868	      0.988960	      4.452301
H  	      0.102256	      0.194695	      5.063088
H  	      0.032418	      1.936098	      4.648942
H  	      1.613423	      1.099056	      4.738974
C  	      0.457106	      0.649882	      3.039821

Zn-dimer_cell
SCF energy -7749.29920104 h, 3 imaginary frequencies
Symbol	       X	             Y	             Z
Zn 	      4.156576	     -0.479001	      0.378899
F  	      7.003838	     -4.017140	      0.682427
F  	      5.783987	     -5.195819	     -0.661900
F  	      5.517448	     -5.369438	      1.485089
F  	      1.330965	      2.999004	     -0.532396
F  	      2.651011	      4.514625	      0.266932
F  	      2.798353	      3.936851	     -1.818350
O  	      5.156459	     -2.203692	      0.652911
O  	      2.380229	     -1.523121	      0.302979
O  	      3.124643	      1.250853	      0.124658
O  	      5.928578	      0.568549	      0.348285
N  	      3.929981	     -0.137438	      2.518136
N  	      4.379490	     -0.832829	     -1.774272
C  	      2.587048	      3.463650	     -0.576071
C  	      7.332477	      2.517751	     -0.177943
C  	      0.371894	     -6.044252	      0.352960
H  	      0.763070	     -6.996318	      0.748965
H  	      0.153796	     -6.193297	     -0.712535
H  	     -0.587652	     -5.850381	      0.857977
C  	      1.368865	     -4.909920	      0.636331
C  	      1.046803	     -3.558372	     -0.109700
C  	      2.329585	     -2.771740	      0.174789
C  	      3.394108	     -3.723566	      0.204200
C  	      4.717026	     -3.369759	      0.447022
C  	      5.776349	     -4.493028	      0.492439
C  	      2.751360	     -5.073902	     -0.079829
H  	      3.335779	     -5.951705	      0.224395
C  	      2.350926	     -5.053812	     -1.582411
H  	      1.986194	     -6.035578	     -1.921298
H  	      3.216218	     -4.795260	     -2.209861
C  	      1.225346	     -3.979604	     -1.613718
H  	      0.275061	     -4.374795	     -2.000695
H  	      1.473361	     -3.104067	     -2.233679
C  	      1.481880	     -4.766150	      2.162745
H  	      2.177331	     -3.971497	      2.468259
H  	      1.840764	     -5.708320	      2.608880
H  	      0.497630	     -4.547481	      2.607743
C  	     -0.248815	     -2.835814	      0.200356
H  	     -0.349187	     -1.920772	     -0.402759
H  	     -0.306554	     -2.544151	      1.260736
H  	     -1.115657	     -3.473085	     -0.023207
C  	      3.616086	      2.371274	     -0.205336
C  	      6.008430	      1.774344	      0.033276
C  	      6.920833	      3.965728	      0.289620
C  	      5.664346	      4.002133	     -0.647121
H  	      5.056463	      4.913552	     -0.577520
C  	      4.962071	      2.709223	     -0.253045
C  	      3.981002	      0.007718	      3.662103
C  	      4.052716	      0.189374	      5.105058
H  	      3.307377	     -0.448080	      5.603891
H  	      3.856462	      1.240464	      5.364639
H  	      5.055156	     -0.086445	      5.465537
C  	      8.543914	      1.844311	      0.438357
H  	      9.469096	      2.397917	      0.212168
H  	      8.651005	      0.819603	      0.050296
H  	      8.448542	      1.763711	      1.532509
C  	      7.390395	      2.694631	     -1.737226
H  	      8.391782	      3.043206	     -2.034015
H  	      7.226756	      1.732020	     -2.245661
C  	      6.287245	      3.747517	     -2.048810
H  	      6.712054	      4.667009	     -2.479986
H  	      5.526945	      3.379617	     -2.751600
C  	      6.575504	      4.061530	      1.784530
H  	      6.165682	      5.058001	      2.018125
H  	      7.477507	      3.925312	      2.403180
H  	      5.831754	      3.316295	      2.100272
C  	      7.965471	      5.051845	     -0.011631
H  	      7.538403	      6.049838	      0.182502
H  	      8.331479	      5.046794	     -1.046735
H  	      8.841811	      4.939594	      0.647153
C  	      4.473949	     -1.583586	     -4.272277
H  	      3.894043	     -0.889203	     -4.898511
H  	      4.046323	     -2.592957	     -4.368440
H  	      5.516016	     -1.601845	     -4.624701
C  	      4.423894	     -1.159811	     -2.880369
Zn 	     -4.014264	      0.474982	      0.028914
F  	     -2.086705	     -2.041687	     -3.265924
F  	     -3.957088	     -3.116825	     -3.455115
F  	     -2.336849	     -3.948867	     -2.277813
F  	     -4.752383	      4.464207	      3.211139
F  	     -6.472238	      4.644836	      1.900570
F  	     -6.267064	      2.918961	      3.189195
O  	     -4.860807	     -1.050845	      1.116151
O  	     -3.155996	     -0.863574	     -1.225033
O  	     -4.875651	      1.770660	      1.333751
O  	     -3.249822	      2.051981	     -1.066791
N  	     -2.220437	      0.505149	      1.231524
N  	     -5.832336	      0.430193	     -1.184626
C  	     -2.818947	      4.430011	     -1.520932
C  	     -5.587102	      3.761680	      2.415818
C  	     -5.805116	     -5.899931	      1.165402
H  	     -5.237377	     -6.744665	      0.740517
H  	     -6.814855	     -5.933590	      0.735253
H  	     -5.902971	     -6.085879	      2.247323
C  	     -5.056213	     -4.582052	      0.910039
C  	     -5.874589	     -3.285529	      1.266686
C  	     -4.967466	     -2.206113	      0.658406
C  	     -4.371617	     -2.779984	     -0.511641
C  	     -3.524088	     -2.066858	     -1.354796
C  	     -2.963204	     -2.789296	     -2.601390
C  	     -4.936789	     -4.192313	     -0.604186
H  	     -4.365748	     -4.895339	     -1.223920
C  	     -6.427247	     -4.032761	     -1.021320
H  	     -6.896655	     -5.003327	     -1.245487
H  	     -6.511125	     -3.413890	     -1.925821
C  	     -7.056575	     -3.366238	      0.237054
H  	     -7.468751	     -2.364648	      0.039018
H  	     -7.876887	     -3.968528	      0.657869
C  	     -3.703742	     -4.674698	      1.633294
H  	     -3.094067	     -3.767804	      1.519725
H  	     -3.118468	     -5.522461	      1.240127
H  	     -3.849936	     -4.851246	      2.711340
C  	     -6.289222	     -3.026734	      2.702625
H  	     -6.783993	     -2.046710	      2.785466
H  	     -5.418045	     -3.001305	      3.375589
H  	     -6.985720	     -3.799223	      3.066339
C  	     -4.812789	      3.030578	      1.295816
C  	     -3.391915	      3.247867	     -0.727173
C  	     -2.536255	      5.447289	     -0.349716
C  	     -3.988279	      5.316911	      0.223939
H  	     -4.184976	      5.900660	      1.132583
C  	     -4.115916	      3.805491	      0.371749
C  	     -1.096520	      0.581265	      1.488161
C  	      0.324811	      0.677671	      1.769540
H  	      0.853675	     -0.199044	      1.361623
H  	      0.494008	      0.749245	      2.853985
H  	      0.744205	      1.560630	      1.268630
C  	     -1.705185	      4.068524	     -2.485790
H  	     -1.387449	      4.943846	     -3.075089
H  	     -2.044341	      3.287284	     -3.183818
H  	     -0.824755	      3.670200	     -1.959520
C  	     -4.095123	      5.060876	     -2.183550
H  	     -3.794410	      5.803518	     -2.939157
H  	     -4.680907	      4.292429	     -2.711603
C  	     -4.865399	      5.711519	     -0.997757
H  	     -5.887961	      5.325590	     -0.883557
H  	     -4.940016	      6.804253	     -1.112236
C  	     -2.162400	      6.862887	     -0.817779
H  	     -2.161548	      7.555769	      0.040344
H  	     -2.839386	      7.279009	     -1.575709
H  	     -1.145950	      6.871871	     -1.243633
C  	     -1.449714	      4.988861	      0.636377
H  	     -1.401854	      5.681968	      1.492821
H  	     -0.456259	      4.984016	      0.161949
H  	     -1.636872	      3.983070	      1.037512
C  	     -8.034666	      0.637112	     -2.570054
H  	     -7.826368	      1.109729	     -3.541566
H  	     -8.765637	      1.251093	     -2.022794
H  	     -8.464431	     -0.360806	     -2.743475
C  	     -6.805406	      0.522377	     -1.798184

Zn-tetramer_2cells_a
SCF energy -15498.60216300 h, 12 imaginary frequencies
Symbol	       X	             Y	             Z
Zn 	     -6.537198	      1.179953	     -0.787006
F  	     -9.213173	     -0.469301	     -4.078901
F  	     -7.538628	     -1.451455	     -5.035880
F  	     -8.153973	      0.527380	     -5.681103
F  	     -3.636121	      2.265643	      2.576933
F  	     -5.188665	      2.798616	      3.985514
F  	     -4.477640	      0.752525	      3.876464
O  	     -7.536897	      0.641336	     -2.448301
O  	     -4.864353	      1.577625	     -1.924900
O  	     -5.516166	      1.749986	      0.872655
O  	     -8.163342	      0.689852	      0.376499
N  	     -7.193757	      3.252165	     -0.925623
N  	     -5.871489	     -0.905984	     -0.657664
C  	     -4.775810	      1.836064	      3.135610
C  	     -9.266973	      0.106427	      2.495190
C  	     -2.977315	      1.395816	     -6.494645
H  	     -3.480205	      1.379943	     -7.476031
H  	     -2.347548	      0.498962	     -6.430564
H  	     -2.304313	      2.267806	     -6.486848
C  	     -4.021112	      1.510626	     -5.372921
C  	     -3.446982	      1.277371	     -3.922771
C  	     -4.747724	      1.202158	     -3.117993
C  	     -5.720033	      0.594200	     -3.969492
C  	     -7.034288	      0.368552	     -3.574434
C  	     -8.005683	     -0.261709	     -4.596748
C  	     -4.997096	      0.290470	     -5.274219
H  	     -5.641211	      0.133348	     -6.148899
C  	     -4.027408	     -0.885806	     -4.966671
H  	     -3.542577	     -1.265029	     -5.879287
H  	     -4.571747	     -1.727807	     -4.515005
C  	     -3.000267	     -0.227895	     -4.000250
H  	     -1.968742	     -0.286945	     -4.376332
H  	     -2.991633	     -0.680845	     -2.996662
C  	     -4.739635	      2.858381	     -5.548035
H  	     -5.511100	      3.035314	     -4.785147
H  	     -5.233230	      2.902132	     -6.532904
H  	     -4.020285	      3.692224	     -5.505248
C  	     -2.395903	      2.227264	     -3.384453
H  	     -2.075598	      1.935602	     -2.372790
H  	     -2.773169	      3.260457	     -3.329760
H  	     -1.502759	      2.226378	     -4.024612
C  	     -5.850554	      1.514541	      2.072032
C  	     -8.128125	      0.649293	      1.624163
C  	     -9.099529	      1.011452	      3.774876
C  	     -7.573362	      0.680554	      3.912314
H  	     -7.058359	      1.187554	      4.738616
C  	     -7.069043	      1.017380	      2.515063
C  	     -7.701975	      4.283942	     -1.023014
C  	     -8.349737	      5.582159	     -1.146693
H  	     -7.857903	      6.176928	     -1.930951
H  	     -8.290056	      6.127558	     -0.192928
H  	     -9.408290	      5.445902	     -1.414662
C  	    -10.613644	      0.026118	      1.801681
H  	    -11.378373	     -0.415546	      2.460259
H  	    -10.540487	     -0.592646	      0.894011
H  	    -10.964408	      1.020395	      1.483655
C  	     -8.696606	     -1.265797	      3.002820
H  	     -9.499687	     -1.846591	      3.482508
H  	     -8.328135	     -1.871061	      2.160330
C  	     -7.577150	     -0.871243	      4.009666
H  	     -7.806872	     -1.215813	      5.029637
H  	     -6.593209	     -1.280433	      3.741816
C  	     -9.384960	      2.501076	      3.524410
H  	     -9.118453	      3.095240	      4.414028
H  	    -10.457297	      2.665882	      3.329923
H  	     -8.819448	      2.907822	      2.674124
C  	     -9.951520	      0.575744	      4.977348
H  	     -9.653399	      1.141892	      5.875494
H  	     -9.871007	     -0.491936	      5.220253
H  	    -11.016832	      0.791824	      4.795717
C  	     -4.943725	     -3.337892	     -0.851734
H  	     -4.171693	     -3.510752	     -0.087068
H  	     -4.498340	     -3.482280	     -1.847589
H  	     -5.756202	     -4.066918	     -0.714473
C  	     -5.463124	     -1.982604	     -0.738092
Zn 	      1.071272	      4.305069	     -0.412788
F  	      0.666906	      0.042636	     -2.016911
F  	      2.471810	      0.369485	     -3.168232
F  	      0.527925	      0.592066	     -4.103794
F  	      0.409494	      8.329796	      2.741402
F  	      2.507907	      7.881047	      3.062968
F  	      1.828590	      8.559503	      1.124020
O  	      1.432895	      5.263889	     -2.195134
O  	      0.808934	      2.547950	     -1.384615
O  	      1.316678	      6.099101	      0.506231
O  	      0.787461	      3.382605	      1.414883
N  	     -1.054704	      4.675941	     -0.500393
N  	      3.224058	      3.930726	     -0.338974
C  	      0.539465	      3.343690	      3.861086
C  	      1.503768	      7.792145	      2.161463
C  	      2.350796	      4.587980	     -7.002252
H  	      2.014202	      3.795295	     -7.691297
H  	      3.448661	      4.595341	     -7.021553
H  	      2.008801	      5.548611	     -7.420504
C  	      1.747708	      4.361975	     -5.606517
C  	      2.334422	      5.294458	     -4.482172
C  	      1.731733	      4.637924	     -3.231675
C  	      1.664511	      3.231267	     -3.496571
C  	      1.216208	      2.306474	     -2.557662
C  	      1.213975	      0.811945	     -2.953744
C  	      2.240460	      3.053584	     -4.896345
H  	      1.976943	      2.117913	     -5.405742
C  	      3.770401	      3.305622	     -4.767213
H  	      4.304858	      3.072406	     -5.701322
H  	      4.201067	      2.672233	     -3.978857
C  	      3.831424	      4.825043	     -4.433376
H  	      4.273314	      5.036731	     -3.447369
H  	      4.422709	      5.387788	     -5.172600
C  	      0.220208	      4.452154	     -5.746053
H  	     -0.306134	      4.314814	     -4.791506
H  	     -0.145134	      3.680129	     -6.443427
H  	     -0.076106	      5.430511	     -6.158048
C  	      2.133679	      6.795584	     -4.566678
H  	      2.538754	      7.285228	     -3.667638
H  	      1.065332	      7.057343	     -4.617590
H  	      2.637071	      7.220296	     -5.450054
C  	      1.255392	      6.324286	      1.746522
C  	      0.763232	      4.010567	      2.496896
C  	     -0.204961	      4.504817	      4.625371
C  	      0.896338	      5.559129	      4.264789
H  	      0.702850	      6.577717	      4.625521
C  	      0.976608	      5.400732	      2.751185
C  	     -2.188098	      4.478531	     -0.394120
C  	     -3.604082	      4.190452	     -0.250421
H  	     -4.123546	      4.347775	     -1.209655
H  	     -4.045695	      4.830960	      0.527074
H  	     -3.743749	      3.133739	      0.014664
C  	     -0.087606	      1.963447	      3.799449
H  	     -0.155125	      1.510273	      4.801692
H  	      0.514986	      1.299330	      3.160358
H  	     -1.099154	      1.995419	      3.367560
C  	      1.964657	      3.398672	      4.517960
H  	      1.981496	      2.774158	      5.424924
H  	      2.724655	      2.986129	      3.836267
C  	      2.184130	      4.905433	      4.840869
H  	      3.080476	      5.323489	      4.361902
H  	      2.281986	      5.078981	      5.923942
C  	     -0.380674	      4.259213	      6.132544
H  	     -0.736420	      5.179469	      6.625529
H  	      0.537319	      3.944629	      6.646597
H  	     -1.140621	      3.481004	      6.309953
C  	     -1.588970	      4.851648	      4.053240
H  	     -1.987092	      5.751922	      4.550421
H  	     -2.308148	      4.036116	      4.225712
H  	     -1.563512	      5.056303	      2.973879
C  	      5.793951	      3.611461	     -0.013078
H  	      5.986166	      2.770213	      0.669524
H  	      6.234359	      4.525572	      0.412629
H  	      6.272141	      3.401664	     -0.981547
C  	      4.360092	      3.788564	     -0.193940
Zn 	     -1.365254	     -3.998780	      0.333428
F  	     -4.041229	     -5.648035	     -2.958467
F  	     -2.366684	     -6.630188	     -3.915446
F  	     -2.982029	     -4.651354	     -4.560669
F  	      1.535823	     -2.913090	      3.697367
F  	     -0.016721	     -2.380117	      5.105949
F  	      0.694304	     -4.426209	      4.996898
O  	     -2.364953	     -4.537397	     -1.327867
O  	      0.307591	     -3.601109	     -0.804466
O  	     -0.344222	     -3.428747	      1.993089
O  	     -2.991398	     -4.488881	      1.496933
N  	     -2.021813	     -1.926569	      0.194811
N  	     -0.699545	     -6.084717	      0.462770
C  	      0.396134	     -3.342670	      4.256044
C  	     -4.095029	     -5.072306	      3.615624
C  	      2.194629	     -3.782917	     -5.374211
H  	      1.691739	     -3.798790	     -6.355597
H  	      2.824396	     -4.679772	     -5.310130
H  	      2.867631	     -2.910928	     -5.366414
C  	      1.150832	     -3.668108	     -4.252487
C  	      1.724962	     -3.901363	     -2.802337
C  	      0.424220	     -3.976575	     -1.997559
C  	     -0.548089	     -4.584534	     -2.849058
C  	     -1.862344	     -4.810181	     -2.454000
C  	     -2.833739	     -5.440443	     -3.476314
C  	      0.174848	     -4.888264	     -4.153785
H  	     -0.469267	     -5.045385	     -5.028465
C  	      1.144536	     -6.064539	     -3.846237
H  	      1.629367	     -6.443763	     -4.758853
H  	      0.600197	     -6.906541	     -3.394571
C  	      2.171677	     -5.406629	     -2.879816
H  	      3.203202	     -5.465678	     -3.255898
H  	      2.180311	     -5.859579	     -1.876228
C  	      0.432309	     -2.320352	     -4.427601
H  	     -0.339156	     -2.143420	     -3.664713
H  	     -0.061286	     -2.276601	     -5.412470
H  	      1.151659	     -1.486510	     -4.384814
C  	      2.776041	     -2.951470	     -2.264019
H  	      3.096346	     -3.243132	     -1.252356
H  	      2.398775	     -1.918277	     -2.209326
H  	      3.669185	     -2.952356	     -2.904178
C  	     -0.678610	     -3.664192	      3.192466
C  	     -2.956181	     -4.529441	      2.744597
C  	     -3.927585	     -4.167281	      4.895310
C  	     -2.401418	     -4.498180	      5.032748
H  	     -1.886415	     -3.991180	      5.859050
C  	     -1.897099	     -4.161353	      3.635497
C  	     -2.530031	     -0.894792	      0.097420
C  	     -3.177793	      0.403426	     -0.026259
H  	     -2.685959	      0.998194	     -0.810517
H  	     -3.118112	      0.948824	      0.927506
H  	     -4.236346	      0.267168	     -0.294228
C  	     -5.441700	     -5.152615	      2.922115
H  	     -6.206429	     -5.594279	      3.580693
H  	     -5.368543	     -5.771379	      2.014445
H  	     -5.792464	     -4.158339	      2.604089
C  	     -3.524662	     -6.444530	      4.123254
H  	     -4.327743	     -7.025324	      4.602942
H  	     -3.156191	     -7.049795	      3.280764
C  	     -2.405206	     -6.049977	      5.130101
H  	     -2.634928	     -6.394547	      6.150071
H  	     -1.421265	     -6.459167	      4.862250
C  	     -4.213016	     -2.677657	      4.644844
H  	     -3.946509	     -2.083494	      5.534463
H  	     -5.285353	     -2.512851	      4.450357
H  	     -3.647504	     -2.270912	      3.794558
C  	     -4.779576	     -4.602989	      6.097783
H  	     -4.481455	     -4.036842	      6.995928
H  	     -4.699063	     -5.670670	      6.340687
H  	     -5.844888	     -4.386910	      5.916151
C  	      0.228219	     -8.516626	      0.268700
H  	      1.000251	     -8.689486	      1.033366
H  	      0.673604	     -8.661014	     -0.727154
H  	     -0.584258	     -9.245652	      0.405961
C  	     -0.291180	     -7.161337	      0.382342
Zn 	      6.243216	     -0.873665	      0.707646
F  	      5.838850	     -5.136098	     -0.896477
F  	      7.643754	     -4.809248	     -2.047798
F  	      5.699869	     -4.586668	     -2.983360
F  	      5.581438	      3.151062	      3.861836
F  	      7.679851	      2.702313	      4.183402
F  	      7.000534	      3.380769	      2.244454
O  	      6.604839	      0.085156	     -1.074700
O  	      5.980878	     -2.630784	     -0.264181
O  	      6.488622	      0.920367	      1.626665
O  	      5.959405	     -1.796128	      2.535317
N  	      4.117240	     -0.502792	      0.620041
N  	      8.396002	     -1.248008	      0.781460
C  	      5.711409	     -1.835043	      4.981520
C  	      6.675712	      2.613412	      3.281897
C  	      7.522740	     -0.590753	     -5.881818
H  	      7.186146	     -1.383439	     -6.570862
H  	      8.620605	     -0.583392	     -5.901119
H  	      7.180745	      0.369877	     -6.300070
C  	      6.919652	     -0.816759	     -4.486083
C  	      7.506366	      0.115724	     -3.361738
C  	      6.903677	     -0.540810	     -2.111241
C  	      6.836455	     -1.947467	     -2.376137
C  	      6.388152	     -2.872259	     -1.437228
C  	      6.385919	     -4.366789	     -1.833310
C  	      7.412404	     -2.125150	     -3.775911
H  	      7.148887	     -3.060821	     -4.285307
C  	      8.942345	     -1.873112	     -3.646779
H  	      9.476802	     -2.106327	     -4.580888
H  	      9.373011	     -2.506500	     -2.858423
C  	      9.003368	     -0.353690	     -3.312942
H  	      9.445258	     -0.142003	     -2.326935
H  	      9.594653	      0.209054	     -4.052166
C  	      5.392152	     -0.726580	     -4.625619
H  	      4.865810	     -0.863919	     -3.671072
H  	      5.026810	     -1.498605	     -5.322993
H  	      5.095838	      0.251777	     -5.037614
C  	      7.305623	      1.616850	     -3.446244
H  	      7.710698	      2.106495	     -2.547204
H  	      6.237276	      1.878609	     -3.497156
H  	      7.809015	      2.041562	     -4.329620
C  	      6.427336	      1.145552	      2.866956
C  	      5.935176	     -1.168166	      3.617330
C  	      4.966983	     -0.673917	      5.745805
C  	      6.068282	      0.380395	      5.385223
H  	      5.874794	      1.398984	      5.745955
C  	      6.148552	      0.221998	      3.871619
C  	      2.983846	     -0.700203	      0.726314
C  	      1.567862	     -0.988281	      0.870013
H  	      1.048398	     -0.830959	     -0.089221
H  	      1.126249	     -0.347774	      1.647508
H  	      1.428195	     -2.044995	      1.135098
C  	      5.084338	     -3.215287	      4.919883
H  	      5.016819	     -3.668461	      5.922126
H  	      5.686930	     -3.879404	      4.280792
H  	      4.072790	     -3.183314	      4.487994
C  	      7.136601	     -1.780061	      5.638394
H  	      7.153440	     -2.404576	      6.545359
H  	      7.896599	     -2.192605	      4.956701
C  	      7.356074	     -0.273300	      5.961304
H  	      8.252420	      0.144755	      5.482336
H  	      7.453930	     -0.099752	      7.044376
C  	      4.791270	     -0.919520	      7.252978
H  	      4.435524	      0.000736	      7.745963
H  	      5.709263	     -1.234104	      7.767031
H  	      4.031323	     -1.697729	      7.430387
C  	      3.582974	     -0.327085	      5.173675
H  	      3.184852	      0.573189	      5.670855
H  	      2.863796	     -1.142617	      5.346146
H  	      3.608432	     -0.122430	      4.094313
C  	     10.965895	     -1.567273	      1.107356
H  	     11.158110	     -2.408521	      1.789958
H  	     11.406303	     -0.653162	      1.533063
H  	     11.444085	     -1.777069	      0.138887
C  	      9.532036	     -1.390170	      0.926494

Zn-tetramer_2cells_b
SCF energy -15498.57160880 h, 11 imaginary frequencies
Symbol	       X	             Y	             Z
Zn 	     -5.466855	     -4.294100	      0.798525
F  	     -6.667543	     -8.678079	      0.559773
F  	     -8.045343	     -8.278378	     -1.060887
F  	     -8.756367	     -8.310903	      0.989456
F  	     -4.182506	      0.079350	      0.431983
F  	     -2.373936	     -0.168651	      1.592232
F  	     -2.337943	     -0.479285	     -0.553935
O  	     -6.302183	     -6.124846	      0.782738
O  	     -7.309144	     -3.452272	      0.412847
O  	     -4.649907	     -2.435836	      0.832775
O  	     -3.603614	     -5.123103	      1.080376
N  	     -5.746080	     -4.059810	      2.945922
N  	     -5.196738	     -4.531562	     -1.365254
C  	     -3.077016	     -0.667902	      0.555021
C  	     -1.145095	     -5.096187	      1.034736
C  	    -12.058197	     -4.446917	     -0.553555
H  	    -12.660479	     -5.343784	     -0.332274
H  	    -12.094681	     -4.282730	     -1.638327
H  	    -12.561043	     -3.590397	     -0.077193
C  	    -10.637925	     -4.618121	      0.007312
C  	     -9.611488	     -3.521406	     -0.473075
C  	     -8.300167	     -4.126259	      0.036589
C  	     -8.439558	     -5.544464	     -0.061276
C  	     -7.438746	     -6.430014	      0.324048
C  	     -7.719743	     -7.944422	      0.207489
C  	     -9.824568	     -5.785983	     -0.644922
H  	    -10.237966	     -6.790381	     -0.487358
C  	     -9.748114	     -5.345489	     -2.134444
H  	    -10.669568	     -5.595273	     -2.682257
H  	     -8.918605	     -5.854668	     -2.646231
C  	     -9.547110	     -3.806699	     -2.017740
H  	    -10.335965	     -3.237691	     -2.530429
H  	     -8.590165	     -3.457560	     -2.435869
C  	    -10.756521	     -4.731899	      1.535806
H  	     -9.784185	     -4.856766	      2.033273
H  	    -11.379558	     -5.600333	      1.806190
H  	    -11.242424	     -3.836837	      1.956890
C  	     -9.853768	     -2.072067	     -0.102228
H  	     -9.071378	     -1.418415	     -0.516428
H  	     -9.863862	     -1.929358	      0.989732
H  	    -10.817330	     -1.722432	     -0.498244
C  	     -3.414662	     -2.162323	      0.761144
C  	     -2.542979	     -4.466960	      1.016202
C  	     -0.331397	     -3.954774	      1.755526
C  	     -0.849775	     -2.848936	      0.772690
H  	     -0.496439	     -1.831759	      0.986188
C  	     -2.356299	     -3.055715	      0.860438
C  	     -5.825050	     -4.096097	      4.096945
C  	     -5.921421	     -4.148714	      5.548920
H  	     -6.955504	     -3.947645	      5.866718
H  	     -5.255556	     -3.397852	      6.000071
H  	     -5.627275	     -5.147271	      5.905552
C  	     -1.093689	     -6.512678	      1.574971
H  	     -0.074544	     -6.927173	      1.517743
H  	     -1.765364	     -7.167002	      0.998144
H  	     -1.426289	     -6.557633	      2.623821
C  	     -0.667428	     -4.932979	     -0.452239
H  	      0.247760	     -5.523254	     -0.614753
H  	     -1.423881	     -5.323146	     -1.150369
C  	     -0.415559	     -3.405447	     -0.612686
H  	      0.641936	     -3.187308	     -0.826980
H  	     -1.010829	     -2.952960	     -1.417773
C  	     -0.748002	     -3.722132	      3.216885
H  	     -0.244381	     -2.827715	      3.619298
H  	     -0.452325	     -4.576096	      3.847914
H  	     -1.830591	     -3.572253	      3.334258
C  	      1.192614	     -4.152237	      1.737677
H  	      1.695124	     -3.240224	      2.100779
H  	      1.603208	     -4.380782	      0.745391
H  	      1.484490	     -4.974217	      2.411266
C  	     -5.247601	     -4.870985	     -3.952687
H  	     -4.913693	     -3.954343	     -4.461370
H  	     -6.274880	     -5.102487	     -4.272180
H  	     -4.587797	     -5.703257	     -4.239998
C  	     -5.213462	     -4.680784	     -2.509637
Zn 	     -9.391993	      2.916306	      0.166876
F  	     -9.617636	      0.119733	     -3.443354
F  	    -11.517033	      1.027821	     -3.951466
F  	    -11.462794	     -0.851294	     -2.869990
F  	     -7.289637	      5.608775	      4.028939
F  	     -7.890459	      7.200248	      2.681904
F  	     -9.386397	      5.943941	      3.611964
O  	    -11.301609	      2.643651	      0.877980
O  	     -9.706266	      1.530043	     -1.275430
O  	     -9.123294	      4.276722	      1.650206
O  	     -7.487901	      3.285828	     -0.547260
N  	     -8.560615	      1.394509	      1.454485
N  	    -10.246849	      4.450079	     -1.135829
C  	     -5.269966	      4.346261	     -0.494153
C  	     -8.174520	      5.934046	      3.062451
C  	    -15.691497	      0.595115	     -0.091891
H  	    -15.946219	     -0.326166	     -0.642054
H  	    -16.220835	      1.425084	     -0.578546
H  	    -16.106391	      0.492033	      0.923887
C  	    -14.165203	      0.769047	     -0.036234
C  	    -13.685788	      2.160518	      0.522181
C  	    -12.187125	      2.092010	      0.194519
C  	    -12.066108	      1.356375	     -1.029143
C  	    -10.845604	      1.140040	     -1.662892
C  	    -10.847379	      0.351962	     -2.992995
C  	    -13.492305	      1.002619	     -1.433302
H  	    -13.595191	      0.174196	     -2.145677
C  	    -14.150813	      2.333933	     -1.897009
H  	    -15.146706	      2.167727	     -2.336527
H  	    -13.534007	      2.823337	     -2.663955
C  	    -14.235367	      3.144356	     -0.570434
H  	    -13.644789	      4.073427	     -0.589448
H  	    -15.269964	      3.431960	     -0.326380
C  	    -13.593806	     -0.432632	      0.732188
H  	    -12.500830	     -0.393854	      0.836842
H  	    -13.846923	     -1.372127	      0.213481
H  	    -14.028563	     -0.490766	      1.743232
C  	    -14.002408	      2.547989	      1.954070
H  	    -13.531639	      3.512174	      2.200809
H  	    -13.607508	      1.809042	      2.668608
H  	    -15.088476	      2.640389	      2.115805
C  	     -8.083779	      4.958901	      1.866900
C  	     -6.691779	      4.071124	      0.014186
C  	     -4.530377	      4.625765	      0.870232
C  	     -5.589035	      5.689190	      1.320547
H  	     -5.419316	      6.124629	      2.313901
C  	     -6.886244	      4.906236	      1.157548
C  	     -7.897552	      0.508475	      1.786543
C  	     -7.050487	     -0.608819	      2.164165
H  	     -7.356403	     -1.518324	      1.622005
H  	     -7.107283	     -0.779626	      3.249244
H  	     -6.011423	     -0.402781	      1.873955
C  	     -4.720140	      3.299166	     -1.444646
H  	     -3.728958	      3.589435	     -1.828793
H  	     -5.398365	      3.169705	     -2.302501
H  	     -4.626085	      2.317039	     -0.957276
C  	     -5.383548	      5.793814	     -1.091868
H  	     -4.474643	      6.032518	     -1.666063
H  	     -6.228110	      5.860265	     -1.795365
C  	     -5.548429	      6.714459	      0.152275
H  	     -6.469737	      7.312903	      0.127471
H  	     -4.705915	      7.416591	      0.252826
C  	     -3.103322	      5.175414	      0.715294
H  	     -2.722769	      5.517167	      1.692441
H  	     -3.019943	      6.018587	      0.016758
H  	     -2.422554	      4.384869	      0.360016
C  	     -4.453792	      3.409720	      1.807566
H  	     -4.045464	      3.713679	      2.785882
H  	     -3.787851	      2.633241	      1.400643
H  	     -5.435533	      2.950821	      1.989352
C  	    -11.092464	      6.453571	     -2.579150
H  	    -10.408505	      6.625600	     -3.423628
H  	    -11.138869	      7.365171	     -1.964649
H  	    -12.096858	      6.235797	     -2.972149
C  	    -10.619625	      5.335773	     -1.774776
Zn 	      9.391913	     -3.062285	      0.239698
F  	      8.191225	     -7.446264	      0.000946
F  	      6.813425	     -7.046563	     -1.619714
F  	      6.102401	     -7.079088	      0.430629
F  	     10.676262	      1.311165	     -0.126844
F  	     12.484832	      1.063164	      1.033405
F  	     12.520824	      0.752530	     -1.112762
O  	      8.556585	     -4.893032	      0.223911
O  	      7.549624	     -2.220457	     -0.145980
O  	     10.208861	     -1.204021	      0.273948
O  	     11.255154	     -3.891289	      0.521549
N  	      9.112687	     -2.827995	      2.387095
N  	      9.662030	     -3.299747	     -1.924081
C  	     11.781752	      0.563913	     -0.003806
C  	     13.713673	     -3.864372	      0.475909
C  	      2.800570	     -3.215102	     -1.112382
H  	      2.198289	     -4.111969	     -0.891101
H  	      2.764087	     -3.050915	     -2.197154
H  	      2.297725	     -2.358582	     -0.636020
C  	      4.220843	     -3.386306	     -0.551515
C  	      5.247279	     -2.289591	     -1.031902
C  	      6.558600	     -2.894444	     -0.522238
C  	      6.419210	     -4.312649	     -0.620103
C  	      7.420021	     -5.198199	     -0.234779
C  	      7.139024	     -6.712608	     -0.351338
C  	      5.034200	     -4.554168	     -1.203749
H  	      4.620802	     -5.558566	     -1.046185
C  	      5.110653	     -4.113674	     -2.693271
H  	      4.189199	     -4.363458	     -3.241084
H  	      5.940163	     -4.622853	     -3.205058
C  	      5.311658	     -2.574884	     -2.576567
H  	      4.522802	     -2.005876	     -3.089256
H  	      6.268603	     -2.225745	     -2.994696
C  	      4.102247	     -3.500084	      0.976979
H  	      5.074582	     -3.624951	      1.474446
H  	      3.479210	     -4.368518	      1.247363
H  	      3.616344	     -2.605022	      1.398064
C  	      5.005000	     -0.840252	     -0.661055
H  	      5.787390	     -0.186600	     -1.075254
H  	      4.994905	     -0.697543	      0.430905
H  	      4.041438	     -0.490617	     -1.057071
C  	     11.444106	     -0.930508	      0.202317
C  	     12.315789	     -3.235145	      0.457375
C  	     14.527371	     -2.722959	      1.196700
C  	     14.008992	     -1.617121	      0.213864
H  	     14.362328	     -0.599944	      0.427361
C  	     12.502469	     -1.823900	      0.301611
C  	      9.033717	     -2.864282	      3.538118
C  	      8.937347	     -2.916899	      4.990093
H  	      7.903264	     -2.715830	      5.307891
H  	      9.603212	     -2.166037	      5.441244
H  	      9.231493	     -3.915456	      5.346725
C  	     13.765079	     -5.280863	      1.016144
H  	     14.784224	     -5.695358	      0.958916
H  	     13.093404	     -5.935187	      0.439317
H  	     13.432479	     -5.325818	      2.064994
C  	     14.191340	     -3.701164	     -1.011066
H  	     15.106528	     -4.291440	     -1.173580
H  	     13.434886	     -4.091331	     -1.709196
C  	     14.443208	     -2.173632	     -1.171513
H  	     15.500703	     -1.955493	     -1.385806
H  	     13.847939	     -1.721145	     -1.976600
C  	     14.110766	     -2.490317	      2.658058
H  	     14.614387	     -1.595900	      3.060471
H  	     14.406443	     -3.344281	      3.289087
H  	     13.028177	     -2.340438	      2.775431
C  	     16.051381	     -2.920422	      1.178850
H  	     16.553892	     -2.008409	      1.541952
H  	     16.461976	     -3.148967	      0.186565
H  	     16.343258	     -3.742402	      1.852439
C  	      9.611167	     -3.639170	     -4.511514
H  	      9.945075	     -2.722528	     -5.020197
H  	      8.583888	     -3.870672	     -4.831007
H  	     10.270971	     -4.471442	     -4.798825
C  	      9.645306	     -3.448969	     -3.068463
Zn 	      5.466774	      4.148121	     -0.391951
F  	      5.241132	      1.351547	     -4.002181
F  	      3.341735	      2.259636	     -4.510293
F  	      3.395973	      0.380521	     -3.428817
F  	      7.569131	      6.840589	      3.470112
F  	      6.968309	      8.432063	      2.123077
F  	      5.472371	      7.175756	      3.053138
O  	      3.557158	      3.875466	      0.319153
O  	      5.152502	      2.761858	     -1.834257
O  	      5.735473	      5.508537	      1.091379
O  	      7.370867	      4.517643	     -1.106087
N  	      6.298153	      2.626324	      0.895658
N  	      4.611919	      5.681894	     -1.694656
C  	      9.588802	      5.578076	     -1.052980
C  	      6.684247	      7.165860	      2.503624
C  	     -0.832729	      1.826930	     -0.650717
H  	     -1.087451	      0.905648	     -1.200881
H  	     -1.362067	      2.656899	     -1.137373
H  	     -1.247624	      1.723848	      0.365060
C  	      0.693565	      2.000862	     -0.595061
C  	      1.172980	      3.392333	     -0.036646
C  	      2.671643	      3.323825	     -0.364308
C  	      2.792660	      2.588190	     -1.587970
C  	      4.013164	      2.371855	     -2.221719
C  	      4.011389	      1.583777	     -3.551822
C  	      1.366463	      2.234434	     -1.992129
H  	      1.263577	      1.406011	     -2.704504
C  	      0.707955	      3.565748	     -2.455836
H  	     -0.287939	      3.399542	     -2.895354
H  	      1.324760	      4.055152	     -3.222781
C  	      0.623400	      4.376171	     -1.129261
H  	      1.213978	      5.305242	     -1.148275
H  	     -0.411197	      4.663775	     -0.885207
C  	      1.264962	      0.799182	      0.173361
H  	      2.357937	      0.837961	      0.278015
H  	      1.011844	     -0.140312	     -0.345346
H  	      0.830204	      0.741049	      1.184405
C  	      0.856360	      3.779804	      1.395243
H  	      1.327129	      4.743989	      1.641983
H  	      1.251260	      3.040857	      2.109781
H  	     -0.229708	      3.872204	      1.556978
C  	      6.774989	      6.190716	      1.308073
C  	      8.166989	      5.302939	     -0.544641
C  	     10.328391	      5.857580	      0.311405
C  	      9.269733	      6.921005	      0.761721
H  	      9.439451	      7.356444	      1.755074
C  	      7.972524	      6.138051	      0.598722
C  	      6.961216	      1.740290	      1.227716
C  	      7.808280	      0.622996	      1.605338
H  	      7.502365	     -0.286509	      1.063178
H  	      7.751485	      0.452189	      2.690417
H  	      8.847344	      0.829034	      1.315128
C  	     10.138628	      4.530981	     -2.003473
H  	     11.129810	      4.821250	     -2.387620
H  	      9.460402	      4.401520	     -2.861328
H  	     10.232682	      3.548854	     -1.516103
C  	      9.475219	      7.025629	     -1.650695
H  	     10.384125	      7.264333	     -2.224890
H  	      8.630658	      7.092080	     -2.354192
C  	      9.310339	      7.946274	     -0.406551
H  	      8.389031	      8.544718	     -0.431356
H  	     10.152853	      8.648406	     -0.306001
C  	     11.755445	      6.407229	      0.156467
H  	     12.135999	      6.748981	      1.133614
H  	     11.838825	      7.250402	     -0.542069
H  	     12.436213	      5.616684	     -0.198811
C  	     10.404976	      4.641534	      1.248739
H  	     10.813304	      4.945494	      2.227055
H  	     11.070916	      3.865056	      0.841816
H  	      9.423235	      4.182636	      1.430525
C  	      3.766304	      7.685386	     -3.137977
H  	      4.450263	      7.857415	     -3.982455
H  	      3.719899	      8.596986	     -2.523476
H  	      2.761910	      7.467612	     -3.530976
C  	      4.239142	      6.567588	     -2.333603

Zn-tetramer_2cells_c
SCF energy -15498.59520810 h, 13 imaginary frequencies
Symbol	       X	             Y	             Z
Zn 	     -3.963024	      1.541508	      1.946848
F  	     -0.340887	     -0.665619	      3.597959
F  	     -0.691326	     -2.352090	      2.287410
F  	     -1.479496	     -2.362500	      4.309644
F  	     -7.375836	      3.631012	     -0.266237
F  	     -6.889426	      5.602824	      0.478020
F  	     -5.986444	      4.882096	     -1.357494
O  	     -2.583974	      0.339616	      2.785349
O  	     -5.216017	     -0.076236	      1.693334
O  	     -5.378279	      2.738845	      1.118984
O  	     -2.685168	      3.148611	      2.097169
N  	     -4.877427	      2.021235	      3.864678
N  	     -3.044126	      1.047503	      0.017440
C  	     -6.375092	      4.510604	     -0.123487
C  	     -1.889450	      5.399549	      1.507807
C  	     -5.603665	     -4.979886	      2.222938
H  	     -5.056594	     -5.673447	      2.883207
H  	     -5.454517	     -5.319158	      1.189638
H  	     -6.675791	     -5.089943	      2.450509
C  	     -5.143122	     -3.537046	      2.481922
C  	     -5.657036	     -2.489143	      1.421375
C  	     -4.827049	     -1.264325	      1.816596
C  	     -3.567738	     -1.755989	      2.277519
C  	     -2.551477	     -0.921807	      2.730962
C  	     -1.246600	     -1.572106	      3.241227
C  	     -3.638482	     -3.270462	      2.141249
H  	     -2.914473	     -3.835906	      2.741850
C  	     -3.585765	     -3.569520	      0.616032
H  	     -3.507210	     -4.648693	      0.413663
H  	     -2.709114	     -3.089148	      0.157775
C  	     -4.938901	     -2.986974	      0.114535
H  	     -5.564218	     -3.741080	     -0.384735
H  	     -4.820144	     -2.158505	     -0.600981
C  	     -5.515017	     -3.187739	      3.932116
H  	     -5.225099	     -2.165744	      4.214846
H  	     -5.014401	     -3.878079	      4.630924
H  	     -6.600586	     -3.290699	      4.091889
C  	     -7.147526	     -2.254228	      1.279580
H  	     -7.361448	     -1.513421	      0.494363
H  	     -7.590460	     -1.883489	      2.217220
H  	     -7.665839	     -3.183279	      1.004395
C  	     -5.198768	      3.915299	      0.683684
C  	     -2.909673	      4.256736	      1.566880
C  	     -2.855743	      6.643667	      1.563945
C  	     -3.741828	      6.114765	      0.383824
H  	     -4.602696	      6.745764	      0.127313
C  	     -4.075050	      4.709779	      0.868671
C  	     -5.198573	      2.305432	      4.936327
C  	     -5.596536	      2.665241	      6.289998
H  	     -6.207497	      1.863022	      6.730319
H  	     -6.182745	      3.596311	      6.277535
H  	     -4.702239	      2.814475	      6.913592
C  	     -0.750749	      5.286035	      2.503500
H  	     -0.026721	      6.107136	      2.380302
H  	     -0.217623	      4.332323	      2.368237
H  	     -1.118311	      5.302670	      3.541470
C  	     -1.456041	      5.404970	     -0.001541
H  	     -0.577737	      6.055888	     -0.132882
H  	     -1.154022	      4.396844	     -0.324606
C  	     -2.703427	      5.943708	     -0.760741
H  	     -2.491658	      6.898542	     -1.265998
H  	     -3.076895	      5.247346	     -1.524234
C  	     -3.618334	      6.779027	      2.891845
H  	     -4.373112	      7.579259	      2.818258
H  	     -2.931973	      7.050698	      3.710377
H  	     -4.142059	      5.856643	      3.180207
C  	     -2.172630	      7.991988	      1.286358
H  	     -2.932431	      8.782850	      1.171011
H  	     -1.549317	      8.000899	      0.382572
H  	     -1.528412	      8.281149	      2.132489
C  	     -2.017957	      0.100187	     -2.187591
H  	     -2.588946	      0.460593	     -3.056329
H  	     -2.055612	     -0.999403	     -2.167000
H  	     -0.970046	      0.420106	     -2.287466
C  	     -2.589153	      0.634011	     -0.959476
Zn 	    -11.565175	     -0.574676	     -0.403526
F  	     -8.092180	     -2.583375	     -2.596354
F  	     -9.388600	     -4.276876	     -2.972385
F  	     -7.986621	     -4.324624	     -1.318394
F  	    -14.400358	      3.213921	      1.644293
F  	    -15.647032	      2.607704	     -0.024966
F  	    -15.271582	      1.233559	      1.603548
O  	    -12.149057	     -2.167708	      0.757594
O  	    -10.008950	     -1.646224	     -1.132007
O  	    -13.124806	      0.461926	      0.381570
O  	    -11.069575	      1.034992	     -1.601531
N  	    -10.288591	      0.240913	      1.136000
N  	    -12.855967	     -1.413826	     -1.955865
C  	    -11.311768	      3.338591	     -2.427182
C  	    -14.708335	      2.170439	      0.844837
C  	    -11.468666	     -6.989558	      1.591028
H  	    -10.565754	     -7.614230	      1.486322
H  	    -12.251167	     -7.435720	      0.963043
H  	    -11.801393	     -7.072679	      2.638449
C  	    -11.140135	     -5.528652	      1.243622
C  	    -12.395073	     -4.584647	      1.134568
C  	    -11.748144	     -3.327594	      0.535139
C  	    -10.697240	     -3.777152	     -0.329079
C  	     -9.921350	     -2.907497	     -1.090302
C  	     -8.833161	     -3.515943	     -2.004711
C  	    -10.731692	     -5.298881	     -0.252845
H  	     -9.816386	     -5.813661	     -0.571628
C  	    -12.014670	     -5.738874	     -1.015270
H  	    -12.066624	     -6.832406	     -1.133947
H  	    -12.034499	     -5.300697	     -2.023123
C  	    -13.150254	     -5.205582	     -0.093392
H  	    -13.787696	     -4.451065	     -0.579870
H  	    -13.818418	     -6.011384	      0.248648
C  	    -10.089310	     -5.041554	      2.253257
H  	     -9.795301	     -3.994943	      2.094441
H  	     -9.178457	     -5.659034	      2.183305
H  	    -10.467706	     -5.133926	      3.284280
C  	    -13.255912	     -4.330381	      2.357320
H  	    -14.040029	     -3.593443	      2.124527
H  	    -12.664325	     -3.914101	      3.187508
H  	    -13.741622	     -5.255355	      2.707528
C  	    -13.459715	      1.645803	      0.100401
C  	    -11.675471	      2.128811	     -1.555423
C  	    -11.709447	      4.516549	     -1.457243
C  	    -13.143011	      3.936121	     -1.207344
H  	    -13.762448	      4.509303	     -0.505312
C  	    -12.817223	      2.508767	     -0.784200
C  	     -9.367625	      0.747753	      1.615355
C  	     -8.190885	      1.383890	      2.180389
H  	     -7.422595	      1.522119	      1.402349
H  	     -7.788372	      0.776231	      3.004103
H  	     -8.450323	      2.385372	      2.549369
C  	     -9.917499	      3.296597	     -3.024124
H  	     -9.744171	      4.153634	     -3.694842
H  	     -9.779084	      2.370911	     -3.604257
H  	     -9.141021	      3.307382	     -2.244545
C  	    -12.482470	      3.383958	     -3.472686
H  	    -12.235508	      4.092989	     -4.278398
H  	    -12.619060	      2.400340	     -3.948430
C  	    -13.720193	      3.843188	     -2.648115
H  	    -14.554503	      3.129068	     -2.687808
H  	    -14.104659	      4.813840	     -2.998488
C  	    -11.691918	      5.907613	     -2.111001
H  	    -12.153233	      6.647469	     -1.435529
H  	    -12.224943	      5.960334	     -3.069661
H  	    -10.655294	      6.235021	     -2.292159
C  	    -10.856226	      4.599200	     -0.181199
H  	    -11.274594	      5.356091	      0.503102
H  	     -9.822370	      4.899907	     -0.410925
H  	    -10.816898	      3.647131	      0.366131
C  	    -14.526737	     -2.179016	     -3.809375
H  	    -14.216592	     -1.778039	     -4.785908
H  	    -15.538021	     -1.812841	     -3.576986
H  	    -14.548310	     -3.277664	     -3.865236
C  	    -13.594017	     -1.751377	     -2.776327
Zn 	     11.580928	      0.669240	      0.824639
F  	     15.203065	     -1.537887	      2.475751
F  	     14.852625	     -3.224359	      1.165202
F  	     14.064456	     -3.234768	      3.187436
F  	      8.168115	      2.758744	     -1.388446
F  	      8.654526	      4.730555	     -0.644188
F  	      9.557508	      4.009828	     -2.479702
O  	     12.959978	     -0.532653	      1.663140
O  	     10.327935	     -0.948504	      0.571125
O  	     10.165673	      1.866576	     -0.003225
O  	     12.858783	      2.276342	      0.974960
N  	     10.666525	      1.148966	      2.742470
N  	     12.499826	      0.175234	     -1.104769
C  	      9.168860	      3.638336	     -1.245695
C  	     13.654502	      4.527280	      0.385599
C  	      9.940287	     -5.852154	      1.100730
H  	     10.487357	     -6.545716	      1.760999
H  	     10.089434	     -6.191426	      0.067429
H  	      8.868160	     -5.962212	      1.328301
C  	     10.400829	     -4.409314	      1.359714
C  	      9.886916	     -3.361411	      0.299166
C  	     10.716903	     -2.136594	      0.694387
C  	     11.976214	     -2.628257	      1.155311
C  	     12.992475	     -1.794076	      1.608754
C  	     14.297352	     -2.444374	      2.119019
C  	     11.905470	     -4.142730	      1.019041
H  	     12.629478	     -4.708174	      1.619641
C  	     11.958187	     -4.441788	     -0.506177
H  	     12.036741	     -5.520962	     -0.708546
H  	     12.834837	     -3.961416	     -0.964434
C  	     10.605051	     -3.859242	     -1.007673
H  	      9.979734	     -4.613349	     -1.506943
H  	     10.723808	     -3.030773	     -1.723189
C  	     10.028935	     -4.060007	      2.809908
H  	     10.318853	     -3.038012	      3.092638
H  	     10.529551	     -4.750347	      3.508715
H  	      8.943366	     -4.162968	      2.969681
C  	      8.396426	     -3.126497	      0.157371
H  	      8.182504	     -2.385690	     -0.627846
H  	      7.953491	     -2.755758	      1.095012
H  	      7.878112	     -4.055547	     -0.117813
C  	     10.345183	      3.043030	     -0.438525
C  	     12.634278	      3.384467	      0.444672
C  	     12.688208	      5.771398	      0.441736
C  	     11.802124	      5.242497	     -0.738384
H  	     10.941255	      5.873496	     -0.994895
C  	     11.468902	      3.837510	     -0.253538
C  	     10.345378	      1.433164	      3.814119
C  	      9.947416	      1.792972	      5.167789
H  	      9.336455	      0.990753	      5.608110
H  	      9.361206	      2.724042	      5.155327
H  	     10.841712	      1.942207	      5.791383
C  	     14.793202	      4.413767	      1.381292
H  	     15.517231	      5.234868	      1.258094
H  	     15.326329	      3.460055	      1.246029
H  	     14.425640	      4.430401	      2.419262
C  	     14.087910	      4.532702	     -1.123750
H  	     14.966214	      5.183620	     -1.255091
H  	     14.389930	      3.524576	     -1.446814
C  	     12.840524	      5.071439	     -1.882949
H  	     13.052293	      6.026274	     -2.388207
H  	     12.467056	      4.375078	     -2.646443
C  	     11.925618	      5.906758	      1.769636
H  	     11.170839	      6.706990	      1.696049
H  	     12.611979	      6.178429	      2.588169
H  	     11.401892	      4.984375	      2.057998
C  	     13.371322	      7.119720	      0.164149
H  	     12.611521	      7.910581	      0.048802
H  	     13.994635	      7.128630	     -0.739636
H  	     14.015540	      7.408881	      1.010281
C  	     13.525995	     -0.772081	     -3.309799
H  	     12.955006	     -0.411676	     -4.178538
H  	     13.488340	     -1.871672	     -3.289209
H  	     14.573905	     -0.452163	     -3.409675
C  	     12.954799	     -0.238258	     -2.081685
Zn 	      3.978776	     -1.446945	     -1.525734
F  	      7.451772	     -3.455644	     -3.718562
F  	      6.155352	     -5.149145	     -4.094593
F  	      7.557331	     -5.196893	     -2.440603
F  	      1.143594	      2.341652	      0.522084
F  	     -0.103080	      1.735436	     -1.147174
F  	      0.272369	      0.361291	      0.481340
O  	      3.394895	     -3.039977	     -0.364614
O  	      5.535002	     -2.518493	     -2.254215
O  	      2.419146	     -0.410342	     -0.740638
O  	      4.474376	      0.162724	     -2.723739
N  	      5.255361	     -0.631356	      0.013792
N  	      2.687984	     -2.286094	     -3.078073
C  	      4.232184	      2.466323	     -3.549391
C  	      0.835617	      1.298171	     -0.277372
C  	      4.075286	     -7.861827	      0.468820
H  	      4.978198	     -8.486498	      0.364114
H  	      3.292785	     -8.307989	     -0.159165
H  	      3.742559	     -7.944948	      1.516240
C  	      4.403817	     -6.400920	      0.121414
C  	      3.148878	     -5.456916	      0.012360
C  	      3.795808	     -4.199863	     -0.587070
C  	      4.846711	     -4.649421	     -1.451287
C  	      5.622601	     -3.779766	     -2.212511
C  	      6.710790	     -4.388211	     -3.126919
C  	      4.812260	     -6.171149	     -1.375053
H  	      5.727566	     -6.685929	     -1.693836
C  	      3.529282	     -6.611143	     -2.137479
H  	      3.477328	     -7.704674	     -2.256156
H  	      3.509452	     -6.172965	     -3.145331
C  	      2.393697	     -6.077851	     -1.215601
H  	      1.756256	     -5.323334	     -1.702079
H  	      1.725534	     -6.883652	     -0.873560
C  	      5.454641	     -5.913822	      1.131048
H  	      5.748651	     -4.867211	      0.972232
H  	      6.365494	     -6.531302	      1.061097
H  	      5.076246	     -6.006195	      2.162071
C  	      2.288040	     -5.202649	      1.235112
H  	      1.503923	     -4.465712	      1.002319
H  	      2.879627	     -4.786370	      2.065300
H  	      1.802330	     -6.127624	      1.585320
C  	      2.084237	      0.773535	     -1.021808
C  	      3.868481	      1.256543	     -2.677632
C  	      3.834504	      3.644281	     -2.579452
C  	      2.400940	      3.063853	     -2.329552
H  	      1.781504	      3.637034	     -1.627521
C  	      2.726729	      1.636498	     -1.906408
C  	      6.176327	     -0.124516	      0.493146
C  	      7.353066	      0.511622	      1.058181
H  	      8.121356	      0.649851	      0.280141
H  	      7.755580	     -0.096037	      1.881894
H  	      7.093628	      1.513103	      1.427161
C  	      5.626453	      2.424328	     -4.146333
H  	      5.799781	      3.281365	     -4.817050
H  	      5.764868	      1.498643	     -4.726466
H  	      6.402931	      2.435113	     -3.366754
C  	      3.061482	      2.511690	     -4.594894
H  	      3.308444	      3.220720	     -5.400607
H  	      2.924892	      1.528072	     -5.070639
C  	      1.823759	      2.970919	     -3.770323
H  	      0.989449	      2.256799	     -3.810016
H  	      1.439293	      3.941572	     -4.120696
C  	      3.852034	      5.035345	     -3.233209
H  	      3.390719	      5.775201	     -2.557737
H  	      3.319009	      5.088066	     -4.191870
H  	      4.888658	      5.362752	     -3.414367
C  	      4.687726	      3.726932	     -1.303407
H  	      4.269358	      4.483822	     -0.619106
H  	      5.721581	      4.027639	     -1.533134
H  	      4.727054	      2.774862	     -0.756077
C  	      1.017215	     -3.051285	     -4.931583
H  	      1.327360	     -2.650308	     -5.908117
H  	      0.005931	     -2.685110	     -4.699195
H  	      0.995642	     -4.149932	     -4.987445
C  	      1.949935	     -2.623645	     -3.898536
```

**10. References**

[1]	R. K. Harris, E. D. Becker, S. M. C. D. Menezes, R. Goodfellow, P. Granger, "NMR Nomenclature – Nuclear Spin Properties and Conventions For Chemical Shifts (IUPAC Recommendations 2001)", *Pure Appl. Chem.* **2018**, *73*, 1795-1818.

[2]	G. R. Fulmer, A. J. M. Miller, N. H. Sherden, H. E. Gottlieb, A. Nudelman, B. M. Stoltz, J. E. Bercaw, K. I. Goldberg, "NMR Chemical Shifts of Trace Impurities: Common Laboratory Solvents, Organics, and Gases in Deuterated Solvents Relevant to the Organometallic Chemist", *Organometallics* **2010**, *29*, 2176-2179.

[3]	E. König, in *Landolt-Börnstein, Zahlenwerte und Funktionen aus Naturwissenschaften und Technik*, Springer-Verlag, Berlin, Göttingen, Heidelberg, **1966**.

[4]	Bruker, *APEX4* V2021.10 0, Bruker AXS Inc., Madison, WI, USA, 2021.

[5]	Bruker, *SAINT* v8.30A, Bruker AXS Inc., Madison, WI, USA, 2012.

[6]	Bruker, *XPREP* V2014/2, Bruker AXS Inc., Madison, WI, USA, 2014.

[7]	Bruker, *SADABS* V2016/2, Bruker AXS Inc., Madison, WI, USA, 2016.

[8]	G. M. Sheldrick, *SHELXL* 2019/1, Bruker AXS Inc., Madison, WI, USA, 2019.

[9]	Gaussian 16 Rev. C.01. M. J. Frisch, G. W. Trucks, H. B. Schlegel, G. E. Scuseria, M. A. Robb, J. R. Cheeseman, G. Scalmani, V. Barone, G. A. Petersson, H. Nakatsuji, X. Li, M. Caricato, A. V. Marenich, J. Bloino, B. G. Janesko, R. Gomperts, B. Mennucci, H. P. Hratchian, J. V. Ortiz, A. F. Izmaylov, J. L. Sonnenberg, Williams, F. Ding, F. Lipparini, F. Egidi, J. Goings, B. Peng, A. Petrone, T. Henderson, D. Ranasinghe, V. G. Zakrzewski, J. Gao, N. Rega, G. Zheng, W. Liang, M. Hada, M. Ehara, K. Toyota, R. Fukuda, J. Hasegawa, M. Ishida, T. Nakajima, Y. Honda, O. Kitao, H. Nakai, T. Vreven, K. Throssell, J. A. Montgomery Jr., J. E. Peralta, F. Ogliaro, M. J. Bearpark, J. J. Heyd, E. N. Brothers, K. N. Kudin, V. N. Staroverov, T. A. Keith, R. Kobayashi, J. Normand, K. Raghavachari, A. P. Rendell, J. C. Burant, S. S. Iyengar, J. Tomasi, M. Cossi, J. M. Millam, M. Klene, C. Adamo, R. Cammi, J. W. Ochterski, R. L. Martin, K. Morokuma, O. Farkas, J. B. Foresman, D. J. Fox. Wallingford, CT, 2016.

[10]	SpecDis v 1.71, T. Bruhn, A. Schaumlöffel, Y. Hemberger, G. Pescitelli, Berlin, Germany, 2017; <https://specdis-software.jimdofree.com/>.

[11]	F. Tobias, T. A. Keiderling, "Role of Side Chains in β-Sheet Self-Assembly into Peptide Fibrils. IR and VCD Spectroscopic Studies of Glutamic Acid-Containing Peptides", *Langmuir* **2016**, *32*, 4653-4661.

[12]	N. V. Ilawe, R. Schweitzer-Stenner, D. DiGuiseppi, B. M. Wong, "Is a cross-β-sheet structure of low molecular weight peptides necessary for the formation of fibrils and peptide hydrogels?", *Phys. Chem. Chem. Phys.* **2018**, *20*, 18158-18168.

[13]	M. Krupová, P. Leszczenko, E. Sierka, S. Emma Hamplová, R. Pelc, V. Andrushchenko, "Vibrational Circular Dichroism Unravels Supramolecular Chirality and Hydration Polymorphism of Nucleoside Crystals", *Chem. Eur. J.* **2022**, *28*, e202201922.

[14]	H. Sato, T. Yajima, A. Yamagishi, "Molecular origin for helical winding of fibrils formed by perfluorinated gelators", *Chem. Commun.* **2011**, *47*, 3736-3738.

1. The use of cobalt(II) acetate hydrate as starting material proved less suitable, affording the same product in slightly lower purity.
2. Zinc nitrate and zinc acetate gave less satisfactory results.
